# Metallacyclobutadienes: Intramolecular Rearrangement from Kinetic to Thermodynamic Isomers

**DOI:** 10.1002/advs.202403940

**Published:** 2024-08-05

**Authors:** Yuanting Cai, Yuhui Hua, Zhengyu Lu, Jiangxi Chen, Dafa Chen, Haiping Xia

**Affiliations:** ^1^ College of Chemistry and Chemical Engineering Xiamen University Xiamen 361005 China; ^2^ Shenzhen Grubbs Institute and Department of Chemistry Southern University of Science and Technology Shenzhen 518005 China; ^3^ Department of Materials Science and Engineering College of Materials Xiamen University Xiamen 361005 China

**Keywords:** intramolecular rearrangement, kinetic isomer, metallacyclobutadiene, metallatetrahedrane, thermodynamic isomer

## Abstract

Metallacyclobutadienes (MCBDs) are key intermediates of alkyne metathesis reactions. There are in principle two isomerization pathway from kinetic to thermodynamic MCBDs, intermolecular and intramolecular. However, systems that simultaneously isolate two kinds of MCBD isomers have not been achieved, thus restricting the mechanistic studies of the isomerization. Here the reactivity of a metallapentalyne that contains an M≡C bond within the aromatic ring, with alkynes to afford a series of MCBD‐fused metallapentalenes is studied. In some cases, both kinetic and thermodynamic products are isolated in the same system, which has never been observed in previous MCBD reactions. Furthermore, the isomerization of MCBD‐fused metallapentalenes is investigated both experimentally and theoretically, indicating that it is an intramolecular process involving a metallatetrahedrane (MTd) intermediate. This research provides experimental evidence demonstrating that one MCBD can undergo intramolecular rearrangement to transform into another.

## Graphical abstract

A series of kinetic and thermodynamic metallacyclobutadiene‐fused metallapentalenes with different regioselectivities is isolated, and the studies of the conversion from the former to the latter indicate that it is an intramolecular process involving a metallatetrahedrane intermediate. This research provides experimental evidence that one metallacyclobutadiene can rearrange to another in an intramolecular way.

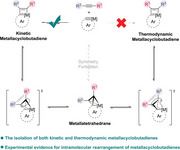

## 1 Introduction

**Figure 1:**
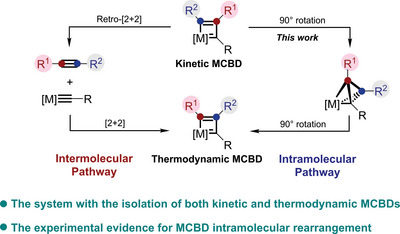
Conversion of kinetic MCBD to thermodynamic one.

Metallacyclobutadienes (MCBDs) are an important class of organometallic complexes and serve as key intermediates in alkyne metathesis reactions.^[^ ^1^ ^]^ For alkyne metathesis reactions of unsymmetric substrates, there are theoretically two kinds of MCBDs caused by different regioselectivities.^[^ ^2^ ^]^ One of the intermediates is a kinetic species with higher energy, and the other is a thermodynamic species with lower energy, in theory, under appropriate conditions, the kinetic MCBD can be converted into the thermodynamic one, through either intermolecular or intramolecular pathways (**Figure** **1**).^[^ ^3^ ^]^ The intermolecular pathway is a generally proposed process, involving a [2 + 2] cycloreversion followed by the [2 + 2] cycloaddition.^[^ ^2^, ^4^ ^]^ On the other hand, the relatively rarely considered intramolecular pathway, initially proposed by Schrock, Churchill, and their co‐workers in 1983,^[^ ^5^ ^]^ is regarded as going through a metallatetrahedrane (MTd) intermediate.^[^ ^6^ ^]^ Despite the assumption of the latter pathway for over four decades and the discovery of many MCBDs during this period,^[^ ^1^, ^2^, ^7^ ^]^ the experimental confirmation of intramolecular conversion between different MCBDs has remained elusive. This is probably because MCBDs are commonly unstable and systems that can simultaneously isolate two MCBD isomers with different regioselectivities are yet to be achieved.

Our research group has been focusing on the research of metallapentalynes in which the M≡C bond is involved in the aromatic rings.^[^ ^8^ ^]^ We found that metallapentalynes can undergo [2 + 2] cycloaddition reactions with terminal alkynes to afford MCBD derivatives.^[^ ^9^ ^]^ Herein, we isolated and identified a series of kinetic and thermodynamic MCBDs in the same system simultaneously for the first time, and explored the transformation from the former to the latter. Experimental studies indicate the conversion is an intramolecular process, and theoretical calculations suggest it undergoes an MTd intermediate. The results provide experimental evidence that one MCBD can intramolecularly isomerize to another.

## 2 Results and Discussion

We first synthesized the osmapentalyne complex **1** bearing a methyl ether group by the means of the reported method.^[^ ^8^, ^10^ ^]^ The single crystal X‐ray diffraction studies show that the bond length of Os1**–**C7 in complex **1** is 1.870(6) Å, which is comparable to the Os≡C bond distances in osmapentalynes (1.777–1.889 Å)^[^ ^8^, ^9^ ^]^ and confirms its triple bond nature. The bond angle of C6**–**C7**–**Os1 is 130.5(5)°, similar to the corresponding angles (127.86°–131.90°)^[^ ^8^, ^9^ ^]^ in osmapentalynes as well, but much smaller than the ideal bond angle toward an sp‐hybridized carbon (180°), revealing the high ring stain and possible high reactivity of complex **1**. The C7 signal in its ^13^C NMR spectrum is located at 330.5 ppm, in consistence with the X‐ray diffraction data.^[^ ^8^ ^]^

**Figure 2:**
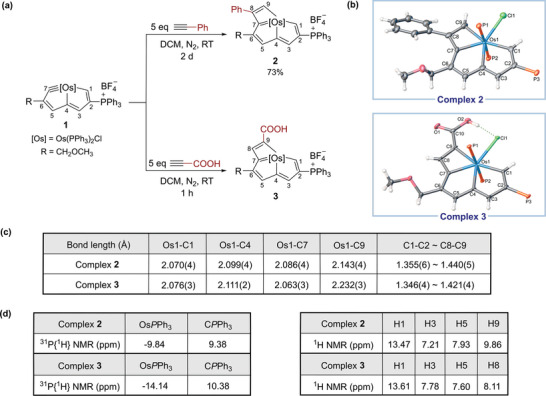
a) [2+2] cycloaddition reactions of complex 1 with terminal alkynes; b) The single crystal structures of the cations of 2 and 3 with 50% probability level, and the phenyl groups in PPh_3_ have been omitted for clarity; c) Selected bond lengths for 2 and 3; d) Selected ^31^P{^1^H} and ^1^H NMR data for 2 and 3.

Treatment of complex **1** with phenylacetylene at room temperature afforded complex **2** as a brown‐green solid in 2 days with a yield of 73% (**Figure** **2**a). The X‐ray diffraction reveals that complex **2** is an osmacyclobutadiene‐fused osmapentalene, and the phenyl group originating from phenylacetylene connects with its C_β_ (C8) (Figure 2b). When phenylacetylene was replaced by propionic acid, the brown solid **3** was isolated in 1 h with a yield of 81% (Figure 2a). Complex **3** is also an osmacyclobutadiene‐fused osmapentalene, while surprisingly, the carboxyl group bonds with its C_α_ (C9) (Figure 2b). The reversed regioselectivities might be caused by the intramolecular hydrogen bond in complex **3**, which is not found in complex **2**, between the carboxyl group and the chloride ligand. Some representative bond lengths of complexes **2** and **3** are shown in Figure 2c, and their Os─C1, Os─C4, and Os─C7 bond lengths are between 2.063(3) and 2.099(4) Å, which are typical for those in osmapentalenes (1.926–2.175 Å).^[^ ^9^ ^]^ Notably, the bond length of Os1**–**C9 in complex **2** (2.143(4) Å) is obviously shorter than that in complex **3** (2.232(3) Å), which may account for the steric effect of the carboxyl group. The C─C bonds within the fused tricyclic rings in these two complexes are intermediate between C─C single and double bonds (1.346(4) to 1.421(4) Å), demonstrating the delocalization of the fused rings. The sum of internal angles in the osmacyclobutadiene rings of complexes **2** and **3** are near 360° (359.98° for **2** and 359.97° for **3**), indicating the planarity of these two rings.

The NMR spectroscopy also supports the structures of complexes **2** and **3** (Figure 2d). The ^31^P{^1^H} signals of Os*P*Ph_3_ and C*P*Ph_3_ in complex **2** are located at −9.84 and 9.38 ppm, respectively, and those in complex **3** are at −14.14 and 10.38 ppm. The H1 atoms resonate at significant downfield positions at 13.47 and 13.61 ppm, respectively, and the chemical shifts of other protons within the fused rings are at 7.21–9.86 ppm. All these data are comparable to those of the previously reported osmacyclobutadiene‐fused osmapentalenes.^[^ ^9a,b^ ^]^

**Figure 3:**
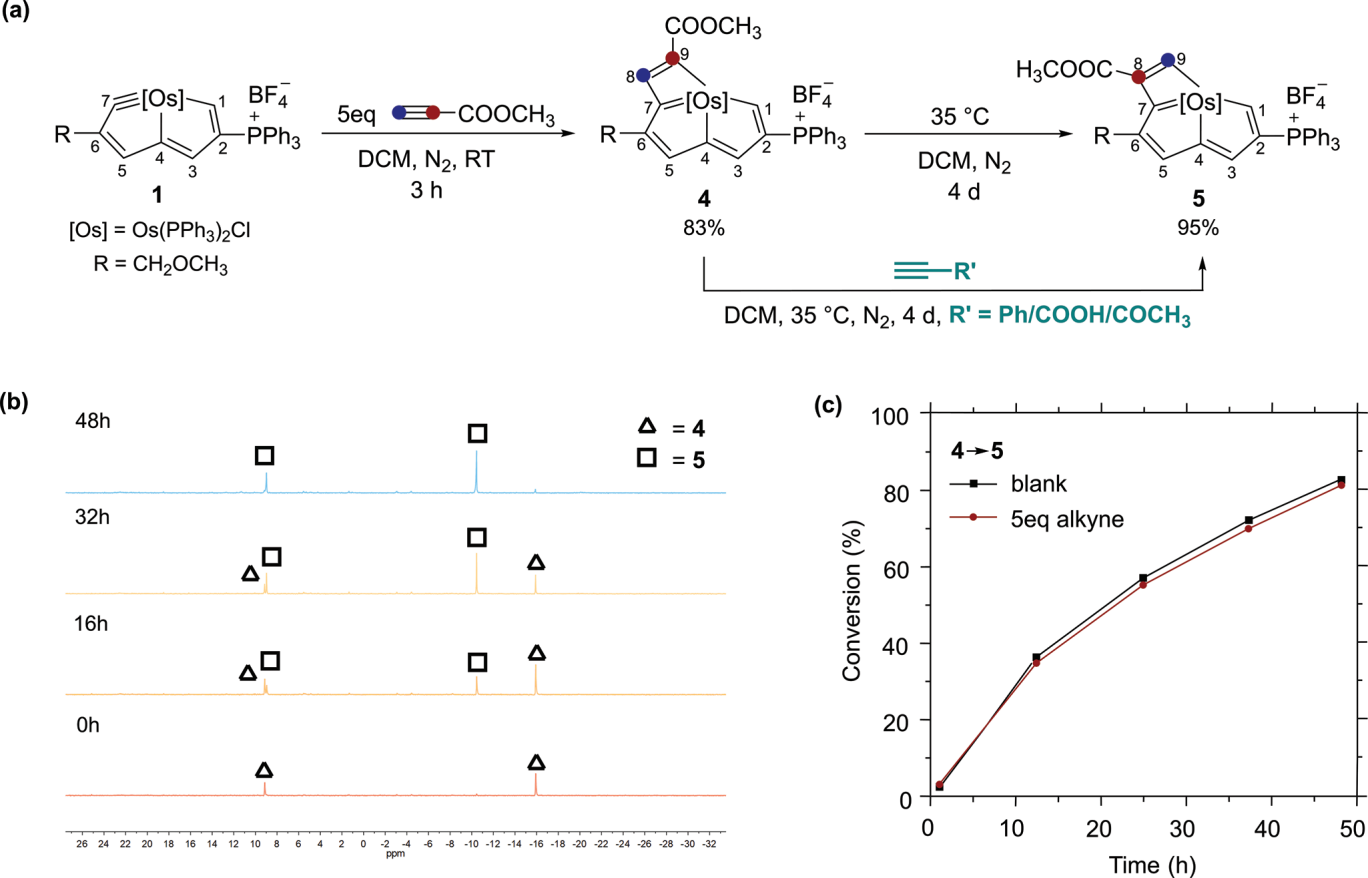
a) Treatment of complex 1 with methyl propiolate; b) In situ ^31^P{^1^H} NMR experiments of the conversion from complex 4 to 5 at 35 °C in CH_2_Cl_2_; c) Control experiments of the conversion from complex 4 to 5 (alkyne is methyl propiolate).

The reactions of **1** with phenylacetylene and propionic acid imply the terminal alkynes have two potential regioselectivities when the [2+2] cycloaddition occurs. In each reaction, however, only one isomer was detected. We wondered whether there were systems where two regioselectivities could be discovered simultaneously because this would provide a direct platform for investigating the isomerization between MCBD derivatives, which might be helpful for mechanistic studies. Following this idea, more terminal alkynes were treated with complex **1**. When methyl propiolate (5 eq.) was selected as the reactant, complex **4** was generated in the first 3 h at room temperature. To our delight, as time went on when the temperature rises to 35 °C, complex **4** gradually isomerized to complex **5**, which could be isolated in 95% yield after 4 days (**Figure** **3**a).

The two ^31^P{^1^H} signals of complex **4** appear at 9.87 (C*P*Ph_3_) and −15.21 ppm (Os*P*Ph_3_), and the ^1^H signal of H8 is at 7.84 ppm, all comparable to those of complex **3**, indicating the ester group links with its C_α_ (C9). Similarly, the characteristic resonance frequencies of complex **5** in its ^31^P{^1^H} (9.71 ppm for C*P*Ph_3_ and −9.71 ppm for Os*P*Ph_3_), and ^1^H NMR (11.09 ppm for H9) spectra point out it has a similar structure to complex **2**, with the ester group at the C_β_ (C8) position.

The transformation from **4** to **5** could be easily monitored by in situ ^31^P{^1^H} NMR spectroscopy. As exhibited in Figure 3b, complex **4** was almost completely converted into **5** after 48 h, and no intermediate could be detected. To investigate whether the conversion is intermolecular or intramolecular, several control experiments were carried out. Initially, excess of some alkynes (5 eq.), including phenylacetylene, propionic acid and 3‐butyn‐2‐one, was added into a solution of **4** at 35 °C in CH_2_Cl_2_, respectively, and besides complex **5**, no other products were detected (Figure 3a). Subsequently, extra methyl propiolate (5 eq.) was treated with **4** under similar conditions, and no significant change in conversion rate was observed (Figure 3c). These results indicate the transformation is intramolecular rather than intermolecular, experimentally confirming that intramolecular isomerization can occur on MCBDs.

**Figure 4:**
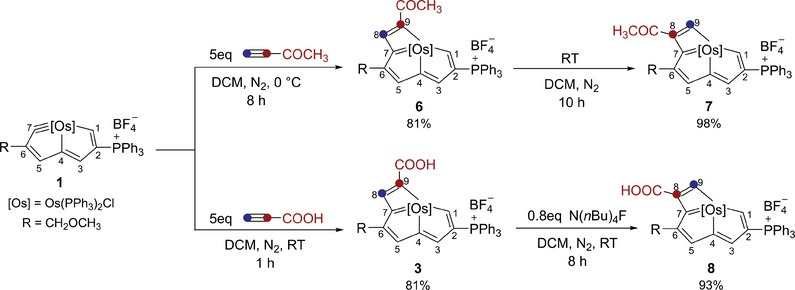
Synthesis of complexes 6 and 3 and their conversion to complexes 7 and 8.

Encouraged by the above results, another alkyne, 3‐butyn‐2‐one, was tested as well, leading to the formation of complex **6**. However, this complex could not be purified at room temperature, because it would partially spontaneously transform into complex **7** under these conditions. Thereafter, the reaction mixture was cooled down to 0 °C, and complex **6** was successfully isolated in 81% yield. When the temperature rose to room temperature, its isomer **7** was afforded in 98% yield (**Figure** **4**).

Both complexes 4 and 6 could be transformed into their thermodynamic isomers 5 and 7, each containing an α‐H in the C9 atom, which prompted us to consider the possibility of converting complex 3 into its isomer. As mentioned previously, there is an intramolecular hydrogen bond between the carboxyl group and the chloride in complex 3, therefore, a hydrogen‐bond‐breaking reagent tetrabutyl ammonium fluoride was added to the solution of 3, and as expected, complex 8 was successfully generated (Figure 4).

To get a better understanding of the mechanism for the intramolecular isomerization on MCBDs, density functional theory (DFT) calculations were conducted. The relative Gibbs free energies of two osmacyclobutadiene‐fused osmapentalene isomers were first calculated, and the results indicate that complexes with a hydrogen at C9 (C_α_) atom are more stable than their isomers with a hydrogen at C8 (C_β_), which is in accordance with the experimental results (Figure S59, Supporting Information).

**Figure 5:**
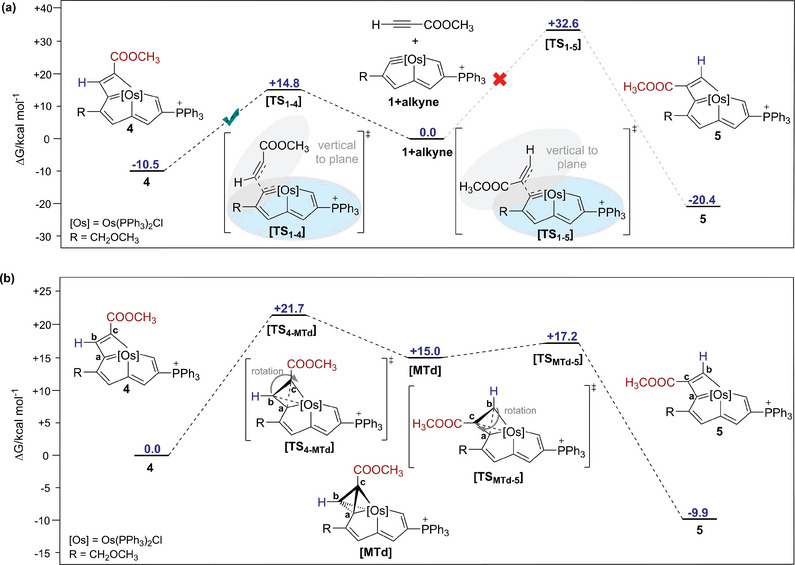
a) Energy profiles for the [2+2] cycloaddition reaction of compound 1 with methyl propiolate (unit: kcal mol^−1^); b) Energy profiles for the conversion reaction of compound 4 to 5 (unit: kcal mol^−1^).

The [2 + 2] cycloaddition reaction paths of compound 1 with methyl propiolate were then investigated, and the free‐energy profile is described in **Figure** **5**a. The terminal sp‐carbon of methyl propiolate approaches the carbyne carbon in a direction perpendicular to the osmapentalyne plane, and the barrier is 14.8 kcal mol^−1^. In contrast, if the internal sp‐carbon attacked the osmapentalyne, although the final product 5 was 9.9 kcal mol^−1^ lower in energy than that for 4, the barrier would be 32.6 kcal mol^−1^, which not only excludes this route but also indicates that the transformation of 4 to 5 is impossible to be an intermolecular process initiated from alkyne dissociation followed by [2 + 2] cycloaddition under the reaction conditions. In addition, other possible [2 + 2] cycloaddition routes were also calculated, but all failed to explain the mechanism (Figure S61, Supporting Information).

An intramolecular mechanism of **4** to **5** was then under consideration. As indicated in Figure 5b, an **[MTd]** intermediate is formed during the reaction, with the energy of the transition state **[TS_4‐MTd_]** as 21.7 kcal mol^−1^, through the change of the Os═C_a_ and C_b_═C_c_ double bonds to single bonds, and the formation of the Os─C_b_ and C_a_─C_c_ bonds. Subsequently, the Os─C_c_ and C_a_─C_b_ single bonds are broken, and meanwhile the Os─C_a_ and C_b_─C_c_ single bonds change to double bonds again, experiencing the transition state **[TS_MTd‐5_]** with the energy barrier of 2.2 kcal mol^−1^. The calculation results are in line with the experimental results, further confirming the intramolecular conversion from **4** to **5** and suggesting an MTd intermediate.

## 3 Conclusion

In summary, through the treatment of an osmapentalyne with alkynes, a series of kinetic and thermodynamic osmacyclobutadiene‐fused osmapentalenes with different regioselectivities have been isolated, and the studies of the conversion from the former to the latter provide experimental evidence that one MCBD can rearrange to another in an intramolecular way. DFT calculations are consistent with the experimental information and suggest an MTd intermediate is involved. These results indicate the aromatic metallapentalene structure can provide a platform for the separation and identification of unstable intermediates.

## Conflict of Interest

The authors declare no conflict of interest.

## Supporting information

Supporting Information

## Data Availability

The data that support the findings of this study are available from the corresponding author upon reasonable request.
