# Supplementary material for: Metallacyclobutadienes: Intramolecular Rearrangement from Kinetic to Thermodynamic Isomers
Source: Adv Sci (Weinh). 2024 Aug 5;11(38):2403940. doi: 10.1002/advs.202403940 (PMC11481178; doi:10.1002/advs.202403940)
Supplement: Supplementary file 1 — Supporting Information [file ADVS-11-2403940-s001.pdf]

ADVANCED SCIENCE
Open Access

Supporting Information

for *Adv. Sci.*, DOI 10.1002/advs.202403940

Metallacyclobutadienes: Intramolecular Rearrangement from Kinetic to Thermodynamic Isomers

Yuanting Cai, Yuhui Hua, Zhengyu Lu, Jiangxi Chen, Dafa Chen and Haiping Xia**

Supporting Information for

Metallacyclobutadienes: Intramolecular Rearrangement from Kinetic to Termodynamic Isomers

Yuanting Cai,[a,†] Yuhui Hua,[a,b,†] Zhengyu Lu,[b,†] Jiangxi Chen,[c] Dafa Chen,[b,*] and Haiping Xia[a,b,*]

[a] College of Chemistry and Chemical Engineering, Xiamen University, Xiamen 361005, China

[b] Shenzhen Grubbs Institute and Department of Chemistry, Southern University of Science and Technology, Shenzhen 518005, China

[c] Department of Materials Science and Engineering, College of Materials, Xiamen University, Xiamen 361005, China

[†] These authors contributed equally to this work.
*Corresponding Authors: xiahp@sustech.edu.cn; chendf@sustech.edu.cn.

Table of Contents

1. General information **2**

2. Experimental procedures **3**

3. NMR spectra and high-resolution mass spectra (HRMS) **12**

4. Crystallographic data **39**

5. Control experiments of the conversion from complex 4 to 5 **45**

6. Computational data **49**

7. References **53**

1. General information

All syntheses were carried out under an inert atmosphere (nitrogen or argon) using standard Schlenk techniques unless otherwise stated. Solvents were distilled under nitrogen from sodium/benzophenone (hexane and diethyl ether) or calcium hydride (dichloromethane) prior to use. The compound **1** were synthesized according to the published literatures.[1,2] The other reagents and solvents were used as purchased from commercial sources without further purification. Column chromatography was performed on silica gel (200–300 mesh) in air. NMR spectra was collected on the Brucker AV-500 (500 MHz) or Brucker AV-600 spectrometer (600 MHz). ^{1}H and ^{13}C{^{1}H} NMR chemical shifts (δ) are relative to tetramethylsilane, and ^{31}P{^{1}H} NMR chemical shifts are relative to 85% H_3PO_4. Two-dimensional and one-dimensional NMR spectra are abbreviated as HSQC (heteronuclear single quantum coherence), HMBC (heteronuclear multiple bond coherence). The absolute values of the coupling constants are given in hertz (Hz). Multiplicities are abbreviated as s (singlet), d (doublet), t (triplet), q (quartet), m (multiplet) and br (broad). Elemental analysis (EA) data were collected on a Vario EL III elemental analyzer. The high-resolution mass spectra (HRMS) experiments were performed on a Bruker En Apex Ultra 7.0T FT-MS.

2. Experimental procedures

Synthesis and characterization of (S2).

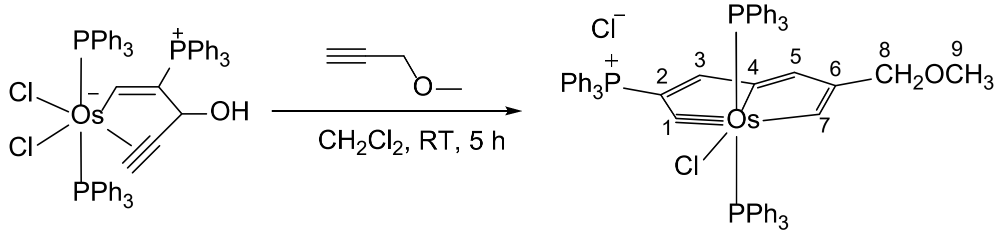

S1

Yield = 62%

S2

To a solution of compound **S1** (1 g 0.89 mmol) in 25 mL dichloromethane, methyl propargyl ether (0.2 mL, 2.86 mmol) was added. The mixture was stirred at RT for 5 h to give a yellow solution. The solution was evaporated under vacuum to a volume of approximately 5 mL and then purified by column chromatography (neutral alumina, eluent: dichloromethane/methanol = 10:1) to give a yellow solution. The yellow solution was evaporated to dryness under vacuum to give the yellow solid. Yield, 652 mg, 62%.

^{1}H, ^{1}H-^{13}C HSQC and ^{1}H-^{13}C HMBC NMR (600.1 MHz, CD_2Cl_2): δ = 13.56 (d, $J_{P\text{-}H}$ = 2.16 Hz, 1H, H7), 8.89 (d, $J_{P\text{-}H}$ = 2.04 Hz, 1H, H5), 7.94 (d, $J_{P\text{-}H}$ = 1.56 Hz, 1H, H3), 4.11 (t, $J_{H\text{-}H}$ = 2.05 Hz, 2H, H8), 2.77 (s, 3H, H9), 7.78–7.04 (45H, other aromatic protons) ppm. **^{31}P{^{1}H} NMR (242.9 MHz, CD_2Cl_2)**: δ = 3.24 (d, $J_{P\text{-}P}$ = 4.63 Hz, Os*P*Ph3), 6.05 (d, $J_{P\text{-}P}$ = 4.79 Hz, C*P*Ph3) ppm. **^{13}C{^{1}H} NMR, ^{1}H-^{13}C HSQC and ^{1}H-^{13}C HMBC (150.9 MHz, CD_2Cl_2)**: δ = 332.1 (dd, $J_{P\text{-}C}$ = 13.8 Hz, $J_{P\text{-}C}$ = 14.1 Hz, C1), 240.0 (d, $J_{P\text{-}C}$ = 10.5 Hz, C7), 183.4 (d, $J_{P\text{-}C}$ = 22.8 Hz, C4), 164.5 (s, C6), 155.2 (s, C5), 152.9 (d, $J_{P\text{-}C}$ = 16.1 Hz, C3), 119.7 (d, $J_{P\text{-}C}$ = 90.3 Hz, C2), 71.9 (s, C8), 57.4 (s, C9), 135.1–127.6 (other aromatic carbons) ppm. **Elemental analysis calcd (%) for $C_{63}H_{53}Cl_2OOsP_3$**: C 64.12, H 4.53; found: C 64.35, H 4.66.

Synthesis and characterization of (1).

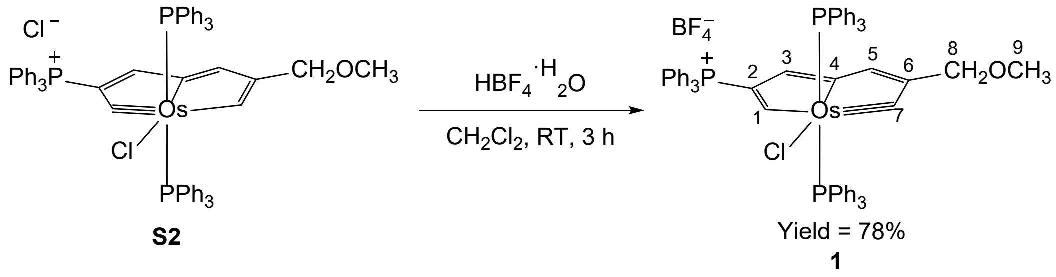

To a solution of compound **S2** (200 mg 0.17 mmol) in 10 mL dichloromethane, $HBF_4 \cdot H_2O$ solution (85 μL, 0.50 mmol) was added. The mixture was stirred at RT for 3 h to give a reddish-brown solution. The solution was evaporated under vacuum to a volume of approximately 2 mL, and then diethyl ether (20 mL) was added to the solution. The yellow precipitate was collected by filtration, washed with diethyl ether (2 × 5 mL) and dryness under vacuum. Yield, 156 mg, 78%.

^{1}H, ^{1}H-^{13}C HSQC and ^{1}H-^{13}C HMBC NMR (600.1 MHz, CD_2Cl_2): δ = 13.03 (d, $J_{P\text{-}H}$ = 18.0 Hz, 1H, H1), 8.48 (s, 1H, H3), 7.73 (s, 1H, H5), 3.75 (s, 2H, H8), 2.80 (s, 3H, H9), 7.79–6.87 (45H, other aromatic protons) ppm. **^{31}P{^{1}H} NMR (242.9 MHz, CD_2Cl_2)**: δ = 6.29 (d, $J_{P\text{-}P}$ = 6.0 Hz, C*P*Ph3), 13.43 (s, Os*P*Ph3) ppm. **^{13}C{^{1}H} NMR, ^{1}H-^{13}C HSQC and ^{1}H-^{13}C HMBC (150.9 MHz, CD_2Cl_2)**: δ = 330.5 (td, $J_{P\text{-}C}$ = 14.4 Hz, $J_{P\text{-}C}$ = 4.9 Hz, C7), 219.5 (br, C1), 181.5 (d, $J_{P\text{-}C}$ = 18.9 Hz, C4), 162.0 (s, C6), 160.0 (s, C5), 141.9 (d, $J_{P\text{-}C}$ = 21.6 Hz, C3), 119.3 (d, $J_{P\text{-}C}$ = 88.7 Hz, C2), 65.8 (s, C8), 57.9 (s, C9), 135.6–127.7 (other aromatic carbons) ppm. **Elemental analysis calcd (%) for $C_{63}H_{53}BClF_4OOsP_3$**: C 61.44, H 4.34; found: C 61.52, H 4.21. **HRMS (ESI)**: *m/z* calcd for $[C_{63}H_{53}ClOOsP_3]^+$, 1145.2607; found, 1145.2623.

Synthesis and characterization of (2).

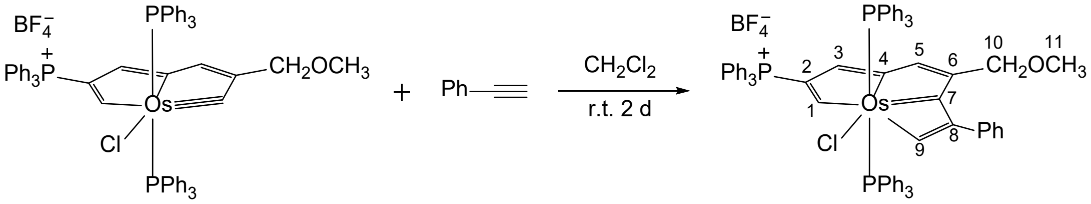

Phenylacetylene (93.3 μL, 0.85 mmol) was added to a solution of compound **1** (200 mg, 0.17 mmol) in 10 mL dichloromethane. The mixture was stirred at RT for 2 d to give a brown solution. The solution was concentrated to ca. 2 mL, and then loaded on silica gel column eluted by dichloromethane/acetone (1:1). The brown band was collected, and the solvent was evaporated to dryness under vacuum to give a brown solid. Yield, 159 mg, 73%.

^{1}H, ^{1}H-^{13}C HSQC and ^{1}H-^{13}C HMBC NMR (500.2 MHz, CD_2Cl_2): δ = 13.47 (d, J_{P-H} = 17.4 Hz, 1H, H1), 9.86 (s, 1H, H9), 7.93 (s, 1H, H5), 7.21 (s, 1H, H3), 3.28 (s, 2H, H10), 2.97 (s, 3H, H11), 7.87–6.32 (50H, other aromatic protons) ppm. **^{31}P{^{1}H} NMR (202.5 MHz, CD_2Cl_2)**: δ = 9.38 (s, C*P*Ph3), -9.84 (s, Os*P*Ph3) ppm. **^{13}C{^{1}H} NMR, ^{1}H-^{13}C HSQC and ^{1}H-^{13}C HMBC (125.8 MHz, CD_2Cl_2)**: δ = 245.9 (s, C1), 194.3 (dt, J_{P-C} = 24.9 Hz, J_{P-C} = 5.5 Hz, C4), 184.2 (t, J_{P-C} = 7.2 Hz, C7), 167.4 (t, J_{P-C} = 6.1 Hz, C8), 165.6 (s, C5), 163.4 (t, J_{P-C} = 10.7 Hz, C9), 160.9 (d, J_{P-C} = 23.6 Hz, C3), 155.6 (s, C6), 120.2 (d, J_{P-C} = 90.0 Hz, C2), 68.6 (s, C10), 58.6 (s, C11), 138.5–126.0 (other aromatic carbons) ppm. **Elemental analysis calcd (%) for $C_{71}H_{59}BClF_4OOsP_3$**: C 63.94, H 4.46; found: C 64.02, H 4.35. **HRMS (ESI)**: *m/z* calcd for $[C_{71}H_{59}ClOOsP_3]^{+}$, 1247.3073; found, 1247.3080.

Synthesis and characterization of (3).

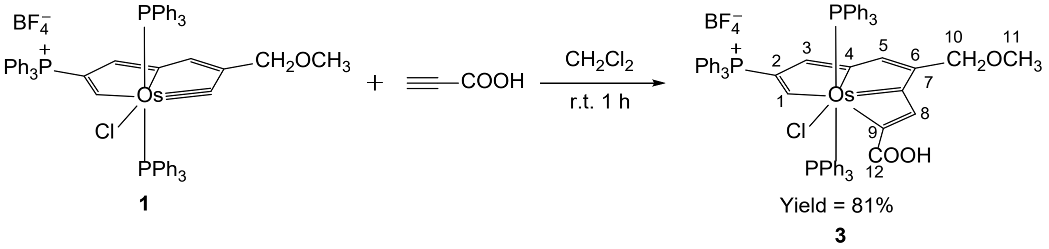

Propiolic acid (75.6 μL, 0.85 mmol) was added to a solution of compound **1** (200 mg, 0.17 mmol) in 10 mL dichloromethane. The mixture was stirred at RT for 1 h to give a brown solution. The solution was concentrated to ca. 2 mL, then diethyl ether (20 mL) was slowly added with stirring to produce a brown precipitate. The brown precipitate was collected by filtration, washed with diethyl ether (2 × 5 mL) and dryness under vacuum. Yield, 171 mg, 86%.

^{1}H, ^{1}H-^{13}C HSQC and ^{1}H-^{13}C HMBC NMR (600.1 MHz, CD_2Cl_2): δ = 13.61 (dd, $J_{P\text{-}H}$ = 15.5 Hz, $J_{P\text{-}H}$ = 3.2 Hz, 1H, H1), 10.31 (br, 1H, COOH), 8.11 (s, 1H, H8), 7.78 (t, $J_{P\text{-}H}$ = 7.5 Hz, 1H, H3), 7.60 (t, $J_{P\text{-}H}$ = 3.0 Hz, 1H, H5), 3.55 (s, 2H, H10), 2.96 (s, 3H, H11), 7.80–6.93 (45H, other aromatic protons) ppm. **^{31}P{^{1}H} NMR (242.9 MHz, CD_2Cl_2)**: δ = 10.38 (s, C*P*Ph$_3$), -14.14 (s, Os*P*Ph$_3$) ppm. **^{13}C{^{1}H} NMR, ^{1}H-^{13}C HSQC and ^{1}H-^{13}C HMBC (150.9 MHz, CD_2Cl_2)**: δ = 239.8 (s, C1), 197.3 (dt, $J_{P\text{-}C}$ = 23.6 Hz, $J_{P\text{-}C}$ = 5.8 Hz, C4), 189.7 (t, $J_{P\text{-}C}$ = 7.4 Hz, C7), 169.4 (s, C9), 164.7 (s, C8), 162.1 (t, $J_{P\text{-}C}$ = 5.5 Hz, C5), 160.8 (d, $J_{P\text{-}C}$ = 21.8 Hz, C3), 157.7 (s, C6), 119.0 (d, $J_{P\text{-}C}$ = 89.4 Hz, C2), 66.7 (s, C10), 58.5 (s, C11), 135.1–127.7 (other aromatic carbons) ppm. **Elemental analysis calcd (%) for $C_{66}H_{55}BClF_4O_3OsP_3$**: C 60.91, H 4.26; found: C 60.77, H 4.19. **HRMS (ESI)**: *m/z* calcd for $[C_{66}H_{55}ClO_3OsP_3]^+$, 1215.2662; found, 1215.2672.

Synthesis and characterization of (4).

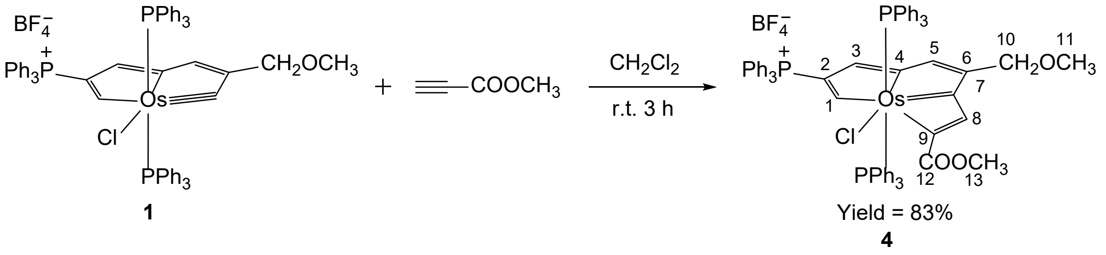

Methyl propionate (75.7 μL, 0.85 mmol) was added to a solution of compound **1** (200 mg, 0.17 mmol) in 10 mL dichloromethane. The mixture was stirred at RT for 3 h to give a brown solution. The solution was concentrated to ca. 2 mL, then diethyl ether (20 mL) was slowly added with stirring to produce a brown precipitate. After it was filtered, the brown solid was washed with diethyl ether (10 mL × 2) again and dried under vacuum. Yield, 190 mg, 83%.

^{1}H, ^{1}H-^{13}C HSQC and ^{1}H-^{13}C HMBC NMR (600.1 MHz, CD_2Cl_2): δ = 13.72 (dd, $J_{P\text{-}H}$ = 15.4 Hz, $J_{P\text{-}H}$ = 3.1 Hz, 1H, H1), 7.84 (s, 1H, H8), 7.76 (t, $J_{P\text{-}H}$ = 7.2 Hz, 1H, H3), 7.32 (t, $J_{P\text{-}H}$ = 3.7 Hz, 1H, H5), 3.40 (s, 2H, H10), 3.33 (s, 3H, H13), 2.99 (s, 3H, H11), 7.85–6.86 (45H, other aromatic protons) ppm. **^{31}P{^{1}H} NMR (242.9 MHz, CD_2Cl_2)**: δ = 9.87 (s, C*P*Ph3), -15.21 (s, Os*P*Ph3) ppm. **^{13}C{^{1}H}, ^{1}H-^{13}C HSQC and ^{1}H-^{13}C HMBC NMR (150.9 MHz, CD2Cl2)**: δ = 241.9 (s, C1), 196.7 (dt, $J_{P\text{-}C}$ = 25.0 Hz, $J_{P\text{-}C}$ = 5.9 Hz, C4), 189.1 (t, $J_{P\text{-}C}$ = 7.2 Hz, C7), 169.2 (s, C9), 162.5 (s, C8), 160.7 (d, $J_{P\text{-}C}$ = 3.9 Hz, C5), 160.1 (d, $J_{P\text{-}C}$ = 21.6 Hz, C3), 156.7 (s, C6), 139.6 (t, $J_{P\text{-}C}$ = 11.3 Hz, C12), 119.3 (d, $J_{P\text{-}C}$ = 89.9 Hz, C2), 66.9 (s, C10), 58.4 (s, C11), 51.0 (s, C13), 134.9–127.5 (other aromatic carbons) ppm. **Elemental analysis calcd (%) for $C_{67}H_{57}BClF_4O_3OsP_3$**: C 61.17, H 4.37; found: C 61.38, H 4.44.

Synthesis and characterization of (5).

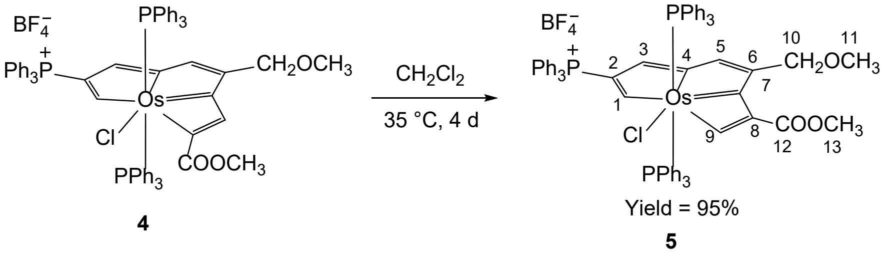

The compound **4** (200 mg, 0.17 mmol) was dissolved in 10 mL dichloromethane. The solution was stirred at 35 °C for 4d to give a brown solution. The solution was concentrated to ca. 2 mL, then diethyl ether (20 mL) was slowly added with stirring to produce a brown precipitate. After it was filtered, the brown solid was washed with diethyl ether (10 mL × 2) again and dried under vacuum. Yield, 190 mg, 95%.

^{1}H, ^{1}H-^{13}C HSQC and ^{1}H-^{13}C HMBC NMR (500.2 MHz, CD_2Cl_2): δ = 13.47 (d, $J_{P\text{-}H}$ = 17.1 Hz, 1H, H1), 11.09 (s, 1H, H9), 8.19 (s, 1H, H5), 7.40 (s, 1H, H3, confirmed by ^{1}H-^{13}C HSQC), 3.81 (s, 2H, H10), 3.44 (s, 3H, H13), 3.11 (s, 3H, H11), 7.86–6.97 (45H, other aromatic protons) ppm. **^{31}P{^{1}H} NMR (202.5 MHz, CD_2Cl_2)**: δ = 9.71 (s, C*P*Ph_3), -9.71 (s, Os*P*Ph_3) ppm. **^{13}C{^{1}H} NMR, ^{1}H-^{13}C HSQC and ^{1}H-^{13}C HMBC (125.8 MHz, CD_2Cl_2)**: δ = 245.1 (s, C1), 194.0 (dt, $J_{P\text{-}C}$ = 24.3 Hz, $J_{P\text{-}C}$ = 4.6 Hz, C4), 185.4 (t, $J_{P\text{-}C}$ = 7.4 Hz, C7), 183.1 (t, $J_{P\text{-}C}$ = 10.7 Hz, C9), 166.3 (s, C5), 161.5 (d, $J_{P\text{-}C}$ = 22.9 Hz, C3), 157.3 (s, C6), 157.0 (s, C12), 154.8 (t, $J_{P\text{-}C}$ = 5.4 Hz, C8), 119.8 (d, $J_{P\text{-}C}$ = 88.8 Hz, C2), 69.9 (s, C10), 58.6 (s, C11), 50.9 (s, C13), 135.3–128.1 (other aromatic carbons) ppm. **Elemental analysis calcd (%) for $C_{67}H_{57}BClF_4O_3OsP_3$**: C 61.17, H 4.37; found: C 61.41, H 4.50. **HRMS (ESI)**: *m/z* calcd for $[C_{67}H_{57}ClO_3OsP_3]^+$, 1229.2818; found, 1229.2828.

Synthesis and characterization of (6).

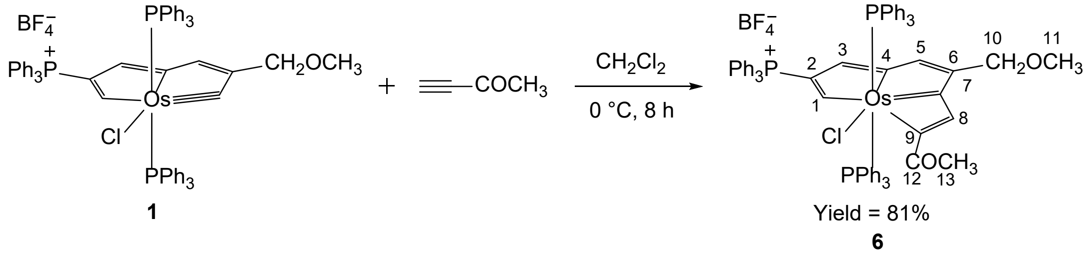

The compound **1** (200 mg, 0.17 mmol) was dissolved in 10 mL dichloromethane with the ice bath to cool down the temperature to 0 ºC. Then 3-butyn-2-one (75.7 µL, 0.85 mmol) was injected to the solution, and the mixture was stirred for 8h at 0 ºC to give a brown solution. The solution was concentrated to ca. 2 mL, then diethyl ether (20 mL) was slowly added with stirring to produce a brown precipitate. After it was filtered, the brown solid was washed with diethyl ether (10 mL × 2) again and dried under vacuum. Yield, 183 mg, 81%.

Attention: Because compound **6** is easy to transform into compound **7** at room temperature, when collecting the NMR data for compound **6**, there was approximate 10% compound **7** mixed in the spectrum. But it does not affect the identification of the NMR of compound **6**.

^{1}H, ^{1}H-^{13}C HSQC and ^{1}H-^{13}C HMBC NMR (600.1 MHz, CD_2Cl_2): δ = 13.73 (dd, $J_{P\text{-}H}$ = 16.0 Hz, $J_{P\text{-}H}$ = 3.7 Hz, 1H, H1), 7.91 (s, 1H, H5), 7.79 (s, 1H, H3), 7.43 (t, $J_{P\text{-}H}$ = 3.3 Hz, 1H, H8), 3.59 (s, 2H, H10), 3.09 (s, 3H, H11), 1.45 (s, 3H, H13), 7.88–7.00 (45H, other aromatic protons) ppm. **^{31}P{^{1}H} NMR (242.9 MHz, CD_2Cl_2)**: δ = 9.86 (s, C*P*Ph3), -16.22 (s, Os*P*Ph3) ppm. **^{13}C{^{1}H}, ^{1}H-^{13}C HSQC and ^{1}H-^{13}C HMBC NMR (150.9 MHz, CD2Cl2)**: δ = 241.6 (s, C1), 203.8 (s, C12), 197.2 (dt, $J_{P\text{-}C}$ = 23.1 Hz, $J_{P\text{-}C}$ = 4.6 Hz, C4), 189.6 (t, $J_{P\text{-}C}$ = 6.8 Hz, C7), 166.3 (s, C9), 162.4 (s, C5), 159.7 (d, $J_{P\text{-}C}$ = 22.7 Hz, C3), 157.9 (t, $J_{P\text{-}C}$ = 4.1 Hz, C8), 156.9 (s, C6), 119.3 (d, $J_{P\text{-}C}$ = 90.0 Hz, C2), 66.9 (s, C10), 58.5 (s, C11), 28.9 (s, C13), 135.2–127.5 (other aromatic carbons) ppm. **Elemental analysis calcd (%) for $C_{67}H_{57}BClF_4O_2OsP_3$**: C 61.92, H 4.42; found: C 62.06, H 4.57.

Synthesis and characterization of (7).

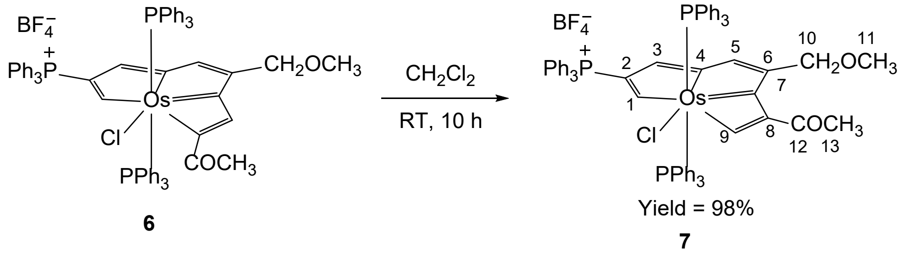

The compound **6** (200 mg, 0.17 mmol) was dissolved in 10 mL dichloromethane. The solution was stirred at RT for 10h to give a brown solution. The solution was concentrated to ca. 2 mL, then diethyl ether (20 mL) was slowly added with stirring to produce a brown precipitate. After it was filtered, the brown solid was washed with diethyl ether (10 mL × 2) again and dried under vacuum. Yield, 196 mg, 98%.

^{1}H, ^{1}H-^{13}C HSQC and ^{1}H-^{13}C HMBC NMR (600.1 MHz, CD_2Cl_2): δ = 13.61 (dd, $J_{P\text{-}H}$ = 17.2 Hz, $J_{P\text{-}H}$ = 4.0 Hz, 1H, H1), 11.01 (s, 1H, H9), 8.10 (s, 1H, H5), 7.38 (s, 1H, H3), 4.03 (s, 2H, H10), 3.19 (s, 3H, H11), 1.50 (s, 3H, H13), 8.10–7.06 (45H, other aromatic protons) ppm. **^{31}P{^{1}H} NMR (242.9 MHz, CD_2Cl_2)**: δ = 9.87 (s, C*P*Ph3), -9.58 (s, Os*P*Ph3) ppm. **^{13}C{^{1}H}, ^{1}H-^{13}C HSQC and ^{1}H-^{13}C HMBC NMR (150.9 MHz, CD_2Cl_2)**: δ = 243.2 (s, C1), 194.4 (dd, $J_{P\text{-}C}$ = 29.6 Hz, $J_{P\text{-}C}$ = 5.0 Hz, C4), 186.6 (s, C7), 185.9 (t, $J_{P\text{-}C}$ = 7.0 Hz, C12), 183.9 (t, $J_{P\text{-}C}$ = 11.1 Hz, C9), 166.2 (s, C5), 163.2 (t, $J_{P\text{-}C}$ = 4.6 Hz, C8), 160.7 (d, $J_{P\text{-}C}$ = 23.2 Hz, C3), 157.9 (s, C6), 119.3 (d, $J_{P\text{-}C}$ = 88.4 Hz, C2), 70.3 (s, C10), 58.2 (s, C11), 22.5 (s, C13), 135.2–127.7 (other aromatic carbons) ppm. **Elemental analysis calcd (%) for $C_{67}H_{57}BClF_4O_2OsP_3$**: C 61.92, H 4.42; found: C 61.73, H 4.61.

Synthesis and characterization of (8).

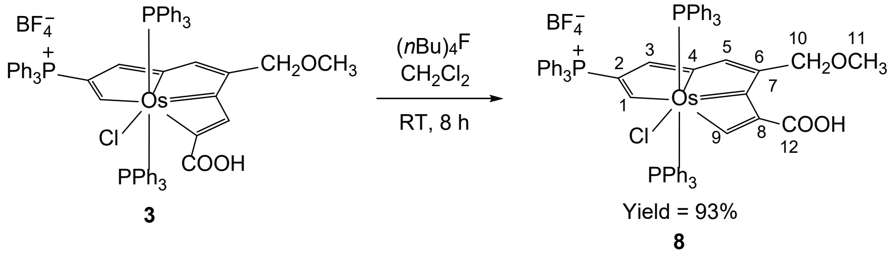

The THF solution of tetrabutylammonium fluoride (1 mol/L, 120 μL, 0.12 mmol) was added to a solution of compound **3** (200 mg, 0.15 mmol) in 10 mL dichloromethane. The mixture was stirred at RT for 8 h to give a brown solution. The solution was concentrated to ca. 2 mL, and then diethyl ether (20 mL) was slowly added with stirring to produce a brown precipitate. After the mixture was filtered, the brown solid was washed with diethyl ether (10 mL × 2) again and dried under vacuum. Yield, 186 mg, 93%.

^{1}H, ^{1}H-^{13}C HSQC and ^{1}H-^{13}C HMBC NMR (500.2 MHz, CD_2Cl_2): δ = 13.37 (d, $J_{P\text{-}H}$ = 17.1 Hz, 1H, H1), 11.16 (s, 1H, H9), 9.38 (br, 1H, H12), 8.02 (s, 1H, H5), 7.29 (s, 1H, H3, confirmed by ^{1}H-^{13}C HSQC), 3.76 (s, 2H, H10), 3.04 (s, 3H, H11), 7.81–6.92 (45H, other aromatic protons) ppm. **^{31}P{^{1}H} NMR** (202.5 MHz, CD_2Cl_2): δ = 9.32 (s, C*P*Ph3), -10.87 (s, Os*P*Ph3) ppm. **^{13}C, ^{1}H-^{13}C HSQC and ^{1}H-^{13}C HMBC NMR (125.8 MHz, CD_2Cl_2)**: δ = 245.2 (s, C1), 193.0 (dt, $J_{P\text{-}C}$ = 24.4 Hz, $J_{P\text{-}C}$ = 6.0 Hz, C4), 187.6 (t, $J_{P\text{-}C}$ = 7.4 Hz, C7), 180.5 (t, $J_{P\text{-}C}$ = 10.7 Hz, C9), 165.8 (s, C5), 160.0 (d, $J_{P\text{-}C}$ = 24.1 Hz, C3), 158.2 (s, C6), 157.7 (s, C8), 157.5 (s, C12), 120.1 (d, $J_{P\text{-}C}$ = 88.7 Hz, C2), 70.3 (s, C10), 58.4 (s, C11), 135.1–128.1 (other aromatic carbons) ppm. **Elemental analysis calcd (%) for $C_{66}H_{55}Cl_2O_3OsP_3$**: C 63.41, H 4.43; found: C 63.46, H 4.22. **HRMS (ESI)**: *m/z* calcd for $[C_{66}H_{55}ClO_3OsP_3]^+$, 1215.2662; found, 1215.2680.

3. NMR spectra and high-resolution mass spectra (HRMS)

Table S1. Selected NMR details for complexes **1**, **2**, **3** and **8.**

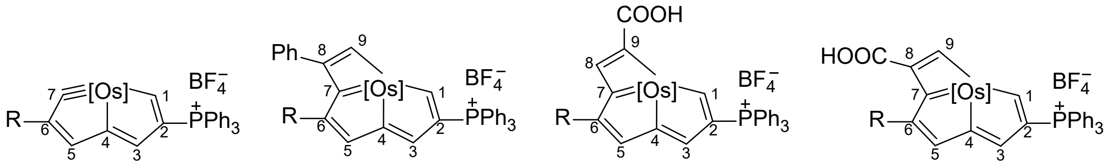

1 **2** **3** **8**

^{1}H NMR/ppm	Complex 1	Complex 2	Complex 3	Complex 8
H1	13.03<br>d, J_{P-H} = 18.0	13.47<br>d, J_{P-H} = 17.4	13.61<br>dd, J_{P-H} = 15.5<br>J_{P-H} = 3.2	13.37<br>d, J_{P-H} = 17.1
H3	8.48<br>s	7.21<br>s	7.78<br>t, J_{P-H} = 7.5	7.29<br>s
H5	7.73<br>s	7.93<br>s	7.60<br>t, J_{P-H} = 3.0	8.02<br>s
H8			8.11<br>s	
H9		9.86<br>s		11.16<br>s
$^{13}C\{^{1}H\}$ NMR/ppm	Complex 1	Complex 2	Complex 3	Complex 8
C1	219.5<br>br	245.9<br>s	239.8<br>s	245.2<br>s
C2	119.3<br>d, J_{P-C} = 88.7	120.2<br>d, J_{P-C} = 90.0	119.0<br>d, J_{P-C} = 89.4	120.1<br>d, J_{P-C} = 88.7
C3	141.9<br>d, J_{P-C} = 21.6	160.9<br>d, J_{P-C} = 23.6	160.8<br>d, J_{P-C} = 21.8	160.0<br>d, J_{P-C} = 24.1
C4	181.5<br>d, J_{P-C} = 18.9	194.3<br>dt, J_{P-C} = 24.9<br>J_{P-C} = 5.5	197.3<br>dt, J_{P-C} = 23.6<br>J_{P-C} = 5.8	193.0<br>dt, J_{P-C} = 24.4<br>J_{P-C} = 6.0
C5	160.0<br>s	165.6<br>s	162.1<br>t, J_{P-C} = 5.5	165.8<br>s
C6	162.0<br>s	155.6<br>s	157.7<br>s	158.2<br>s
C7	330.5<br>td, J_{P-C} = 14.4<br>J_{P-C} = 4.9	184.2<br>t, J_{P-C} = 7.2	189.7<br>t, J_{P-C} = 7.4	187.6<br>t, J_{P-C} = 7.4
C8		167.4<br>t, J_{P-C} = 6.1	164.7<br>s	157.7<br>s
C9		163.4<br>t, J_{P-C} = 10.7	169.4<br>s	180.5<br>t, J_{P-C} = 10.7

Table S2. Selected NMR details for complexes **4**, **5**, **6** and **7.**

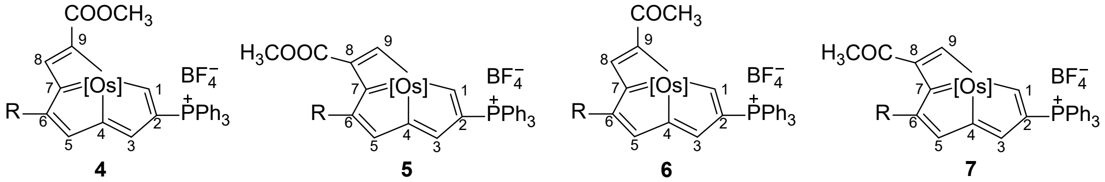

^{1}H NMR/ppm	Complex 4	Complex 5	Complex 6	Complex 7
H1	13.72<br>dd, $J_{P\text{-}H}$ = 15.4<br>$J_{P\text{-}H}$ = 3.1	13.47<br>d, $J_{P\text{-}H}$ = 17.1	13.73<br>dd, $J_{P\text{-}H}$ = 16.0<br>$J_{P\text{-}H}$ = 3.7	13.61<br>dd, $J_{P\text{-}H}$ = 17.2<br>$J_{P\text{-}H}$ = 4.0
H3	7.76<br>t, $J_{P\text{-}H}$ = 7.2	7.40<br>s	7.79<br>s	7.38<br>s
H5	7.32<br>t, $J_{P\text{-}H}$ = 3.7	8.19<br>s	7.91<br>s	8.10<br>s
H8	7.84<br>s		7.43<br>t, $J_{P\text{-}H}$ = 3.3	
H9		11.09<br>s		11.01<br>s
^{13}C{^{1}H} NMR/ppm	**Complex 4**	**Complex 5**	**Complex 6**	**Complex 7**
C1	241.9<br>s	245.1<br>s	241.6<br>s	243.2<br>s
C2	119.3<br>d, $J_{P\text{-}C}$ = 89.9	119.8<br>d, $J_{P\text{-}C}$ = 88.8	119.3<br>d, $J_{P\text{-}C}$ = 90.0	119.3<br>d, $J_{P\text{-}C}$ = 88.4
C3	160.1<br>d, $J_{P\text{-}C}$ = 21.6	161.5<br>d, $J_{P\text{-}C}$ = 22.9	159.7<br>d, $J_{P\text{-}C}$ = 22.7	160.7<br>d, $J_{P\text{-}C}$ = 23.2
C4	196.7<br>dt, $J_{P\text{-}C}$ = 25.0<br>$J_{P\text{-}C}$ = 5.9	194.0<br>dt, $J_{P\text{-}C}$ = 24.3<br>$J_{P\text{-}C}$ = 4.6	197.2<br>dt, $J_{P\text{-}C}$ = 23.1<br>$J_{P\text{-}C}$ = 4.6	194.4<br>dd, $J_{P\text{-}C}$ = 29.6<br>$J_{P\text{-}C}$ = 5.0
C5	160.7<br>d, $J_{P\text{-}C}$ = 3.9	166.3<br>s	162.4<br>s	166.2<br>s
C6	156.7<br>s	157.3<br>s	156.9<br>s	157.9<br>s
C7	189.1<br>t, $J_{P\text{-}C}$ = 7.2	185.4<br>t, $J_{P\text{-}C}$ = 7.4	189.6<br>t, $J_{P\text{-}C}$ = 6.8	186.6<br>s
C8	162.5<br>s	154.8<br>t, $J_{P\text{-}C}$ = 5.4	157.9<br>t, $J_{P\text{-}C}$ = 6.8	163.2<br>t, $J_{P\text{-}C}$ = 4.6
C9	169.2<br>s	183.1<br>t, $J_{P\text{-}C}$ = 10.7	166.3<br>s	183.9<br>t, $J_{P\text{-}C}$ = 11.1

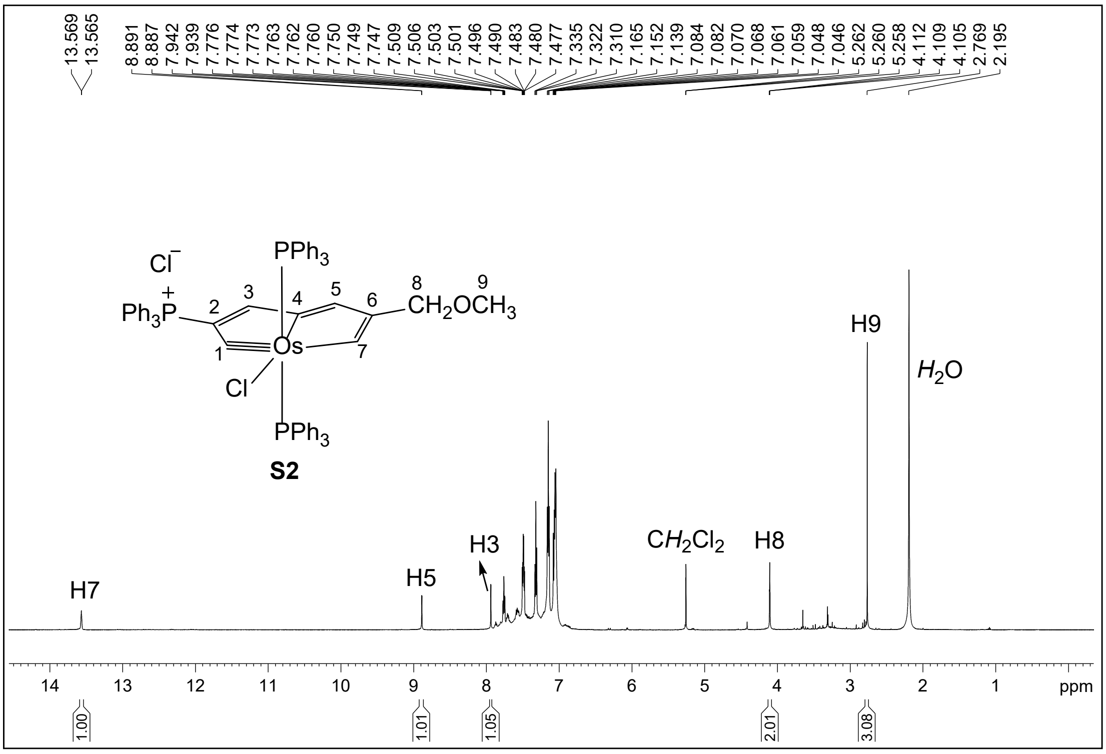

Figure S1 The ^{1}H NMR (600.1 MHz, CD_2Cl_2) spectrum for complex **S2**.

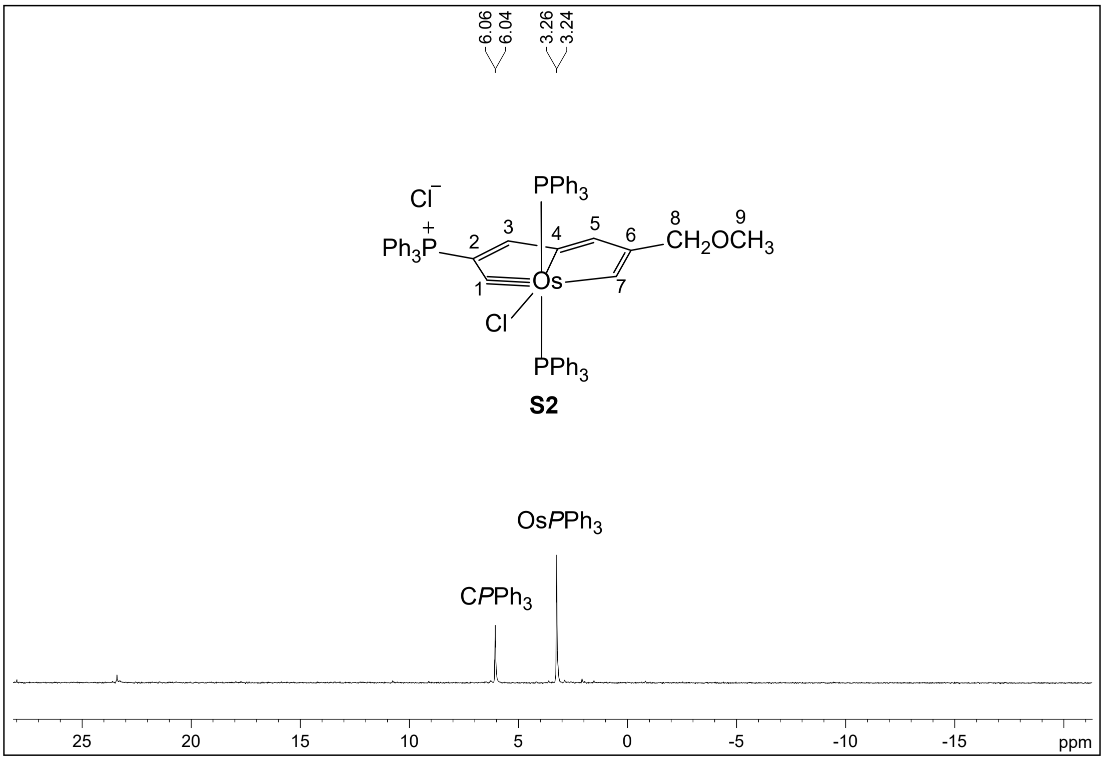

Figure S2 The $^{31}P\{^1H\}$ NMR (242.9 MHz, CD_2Cl_2) spectrum for complex **S2**.

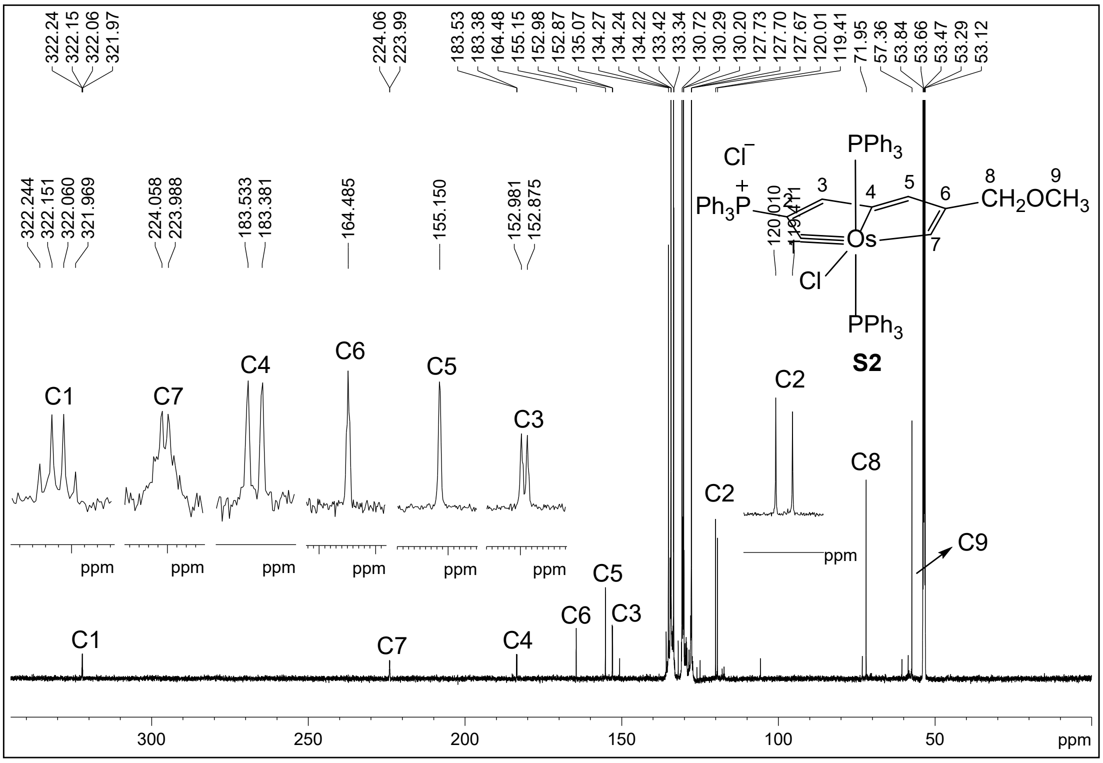

Figure S3 The $^{13}C\{^{1}H\}$ NMR (150.9 MHz, CD_2Cl_2) spectrum for complex **S2**.

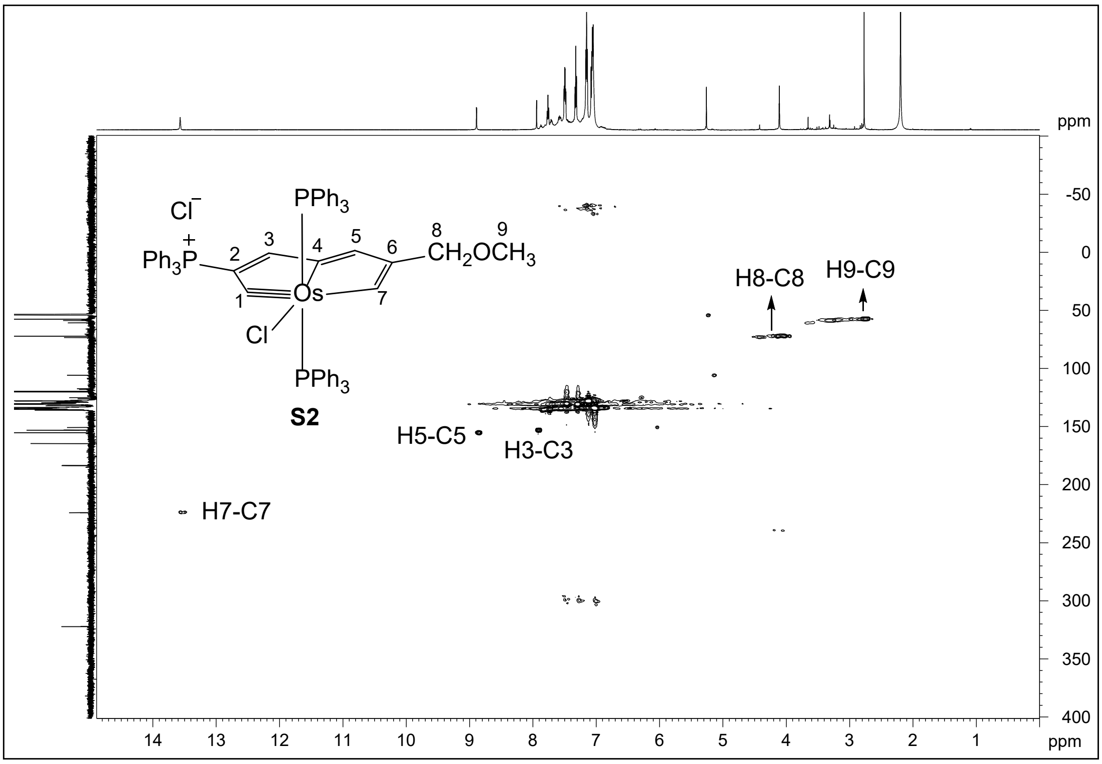

Figure S4 The two-dimensional ^{1}H-^{13}C HSQC spectrum for complex **S2** in CD_2Cl_2.

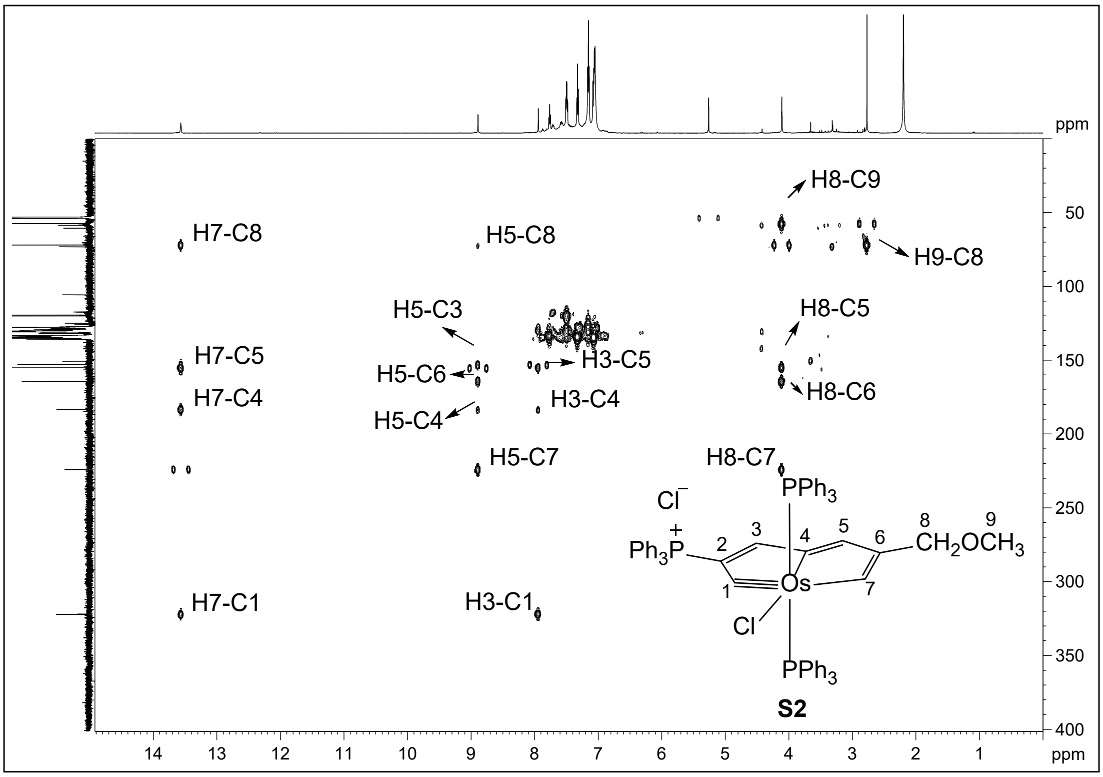

Figure S5 The two-dimensional ^{1}H-^{13}C HMBC spectrum for complex **S2** in CD_2Cl_2.

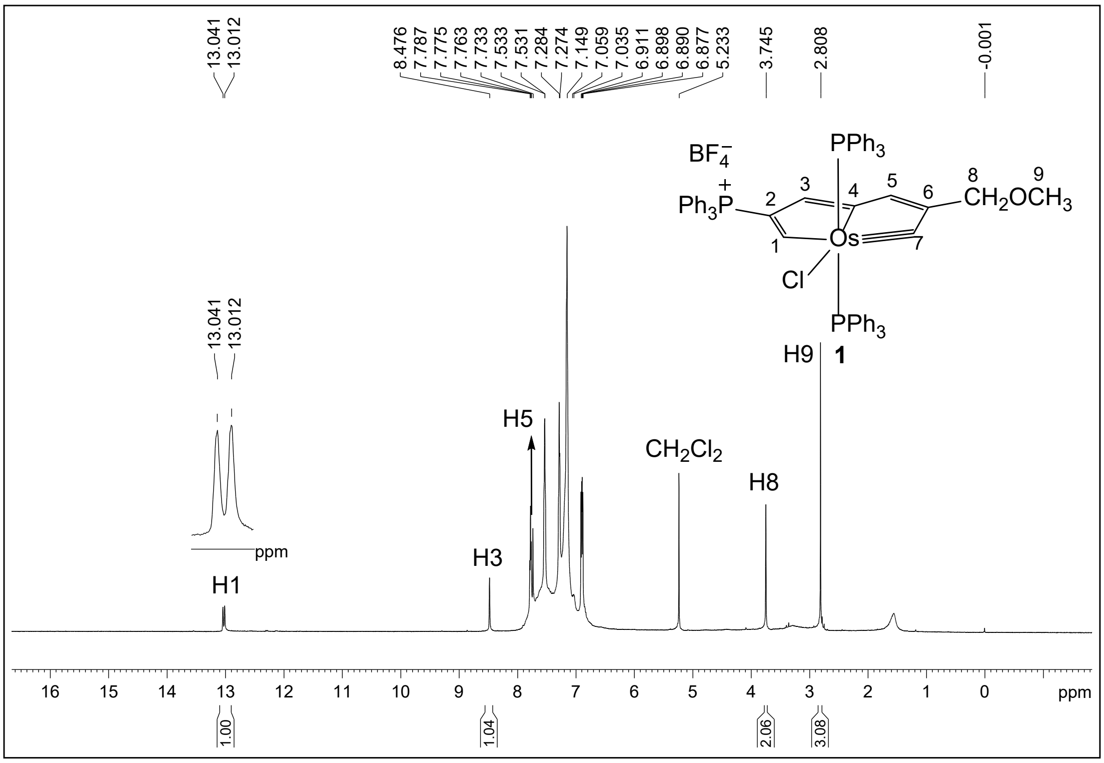

Figure S6 The ^{1}H NMR (600.1 MHz, CD_2Cl_2) spectrum for complex **1**.

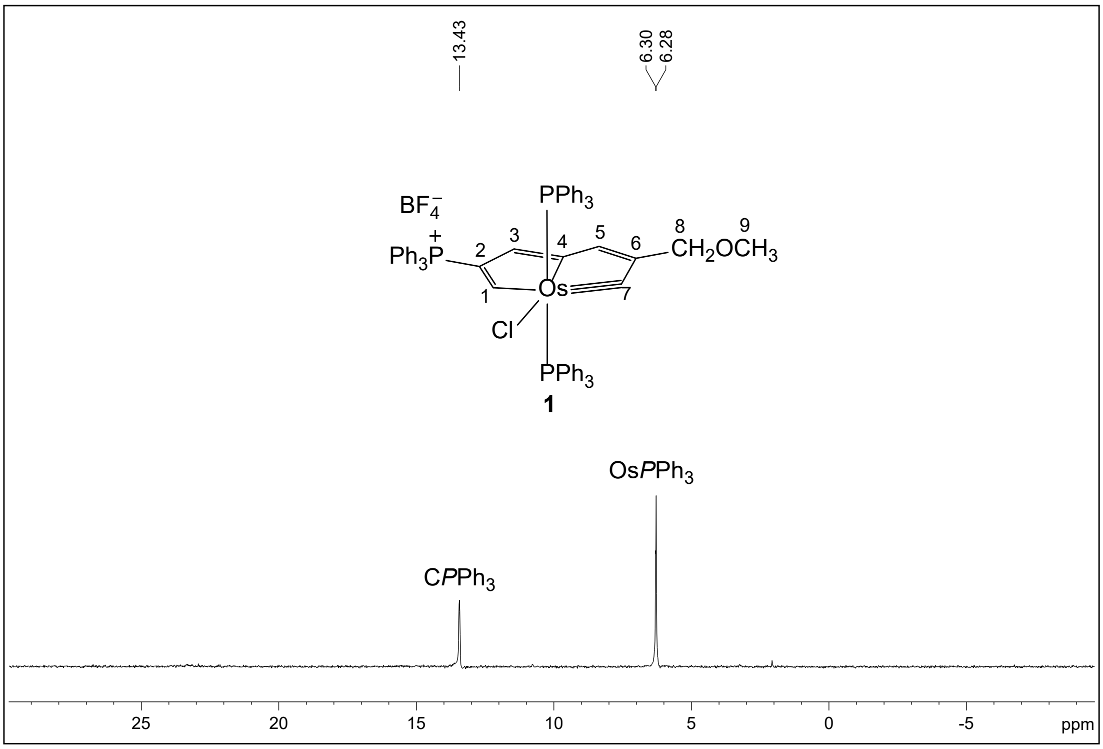

Figure S7 The $^{31}P\{^1H\}$ NMR (242.9 MHz, CD_2Cl_2) spectrum for complex **1**.

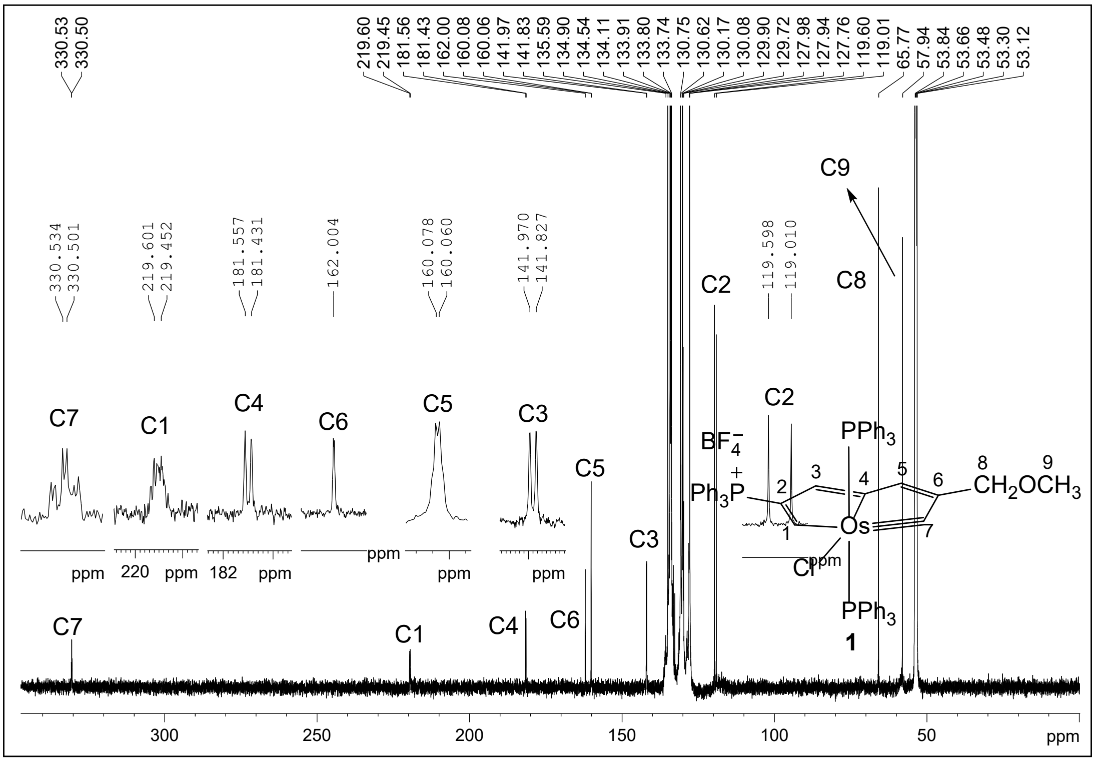

Figure S8 The $^{13}C\{^1H\}$ NMR (150.9 MHz, CD_2Cl_2) spectrum for complex **1**.

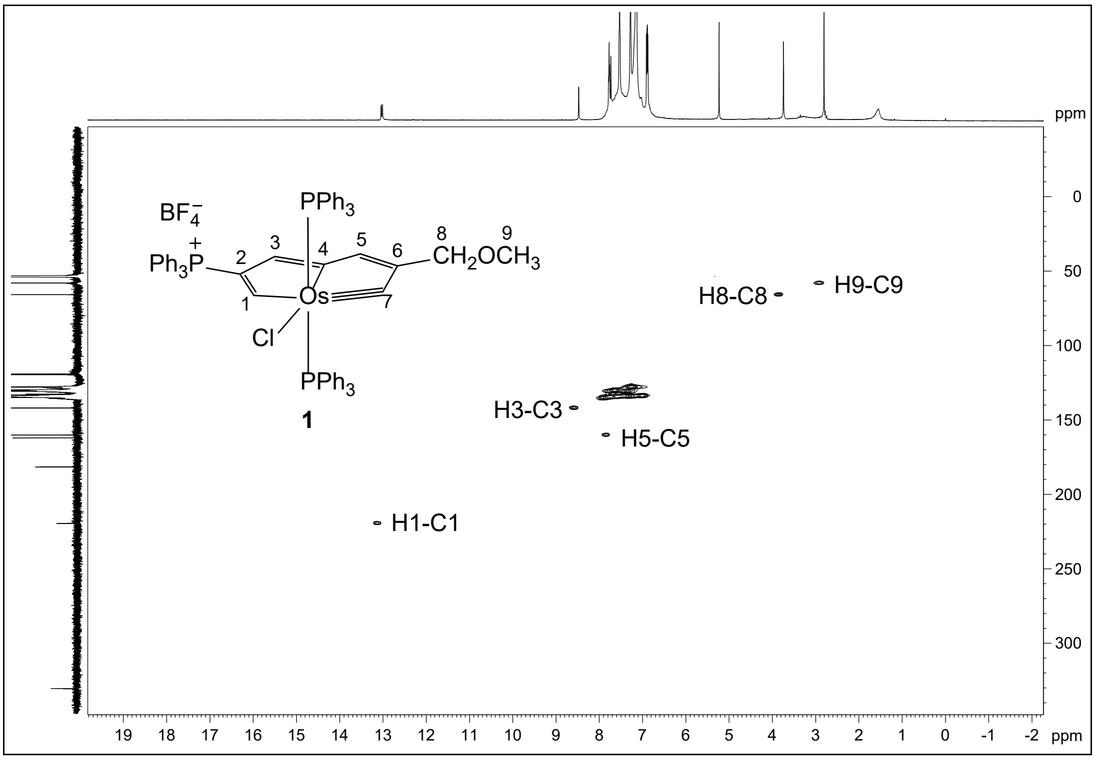

Figure S9 The two-dimensional ^{1}H-^{13}C HSQC spectrum for complex **1** in CD_2Cl_2.

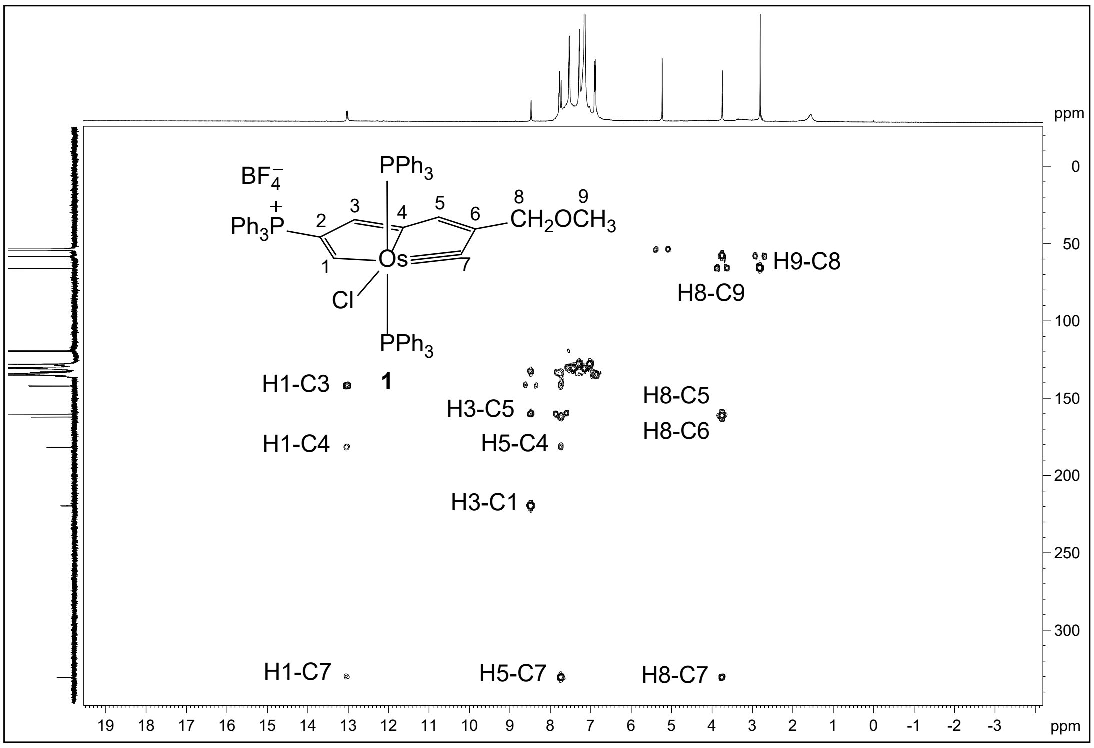

Figure S10 The two-dimensional ^{1}H-^{13}C HMBC spectrum for complex **1** in CD_2Cl_2.

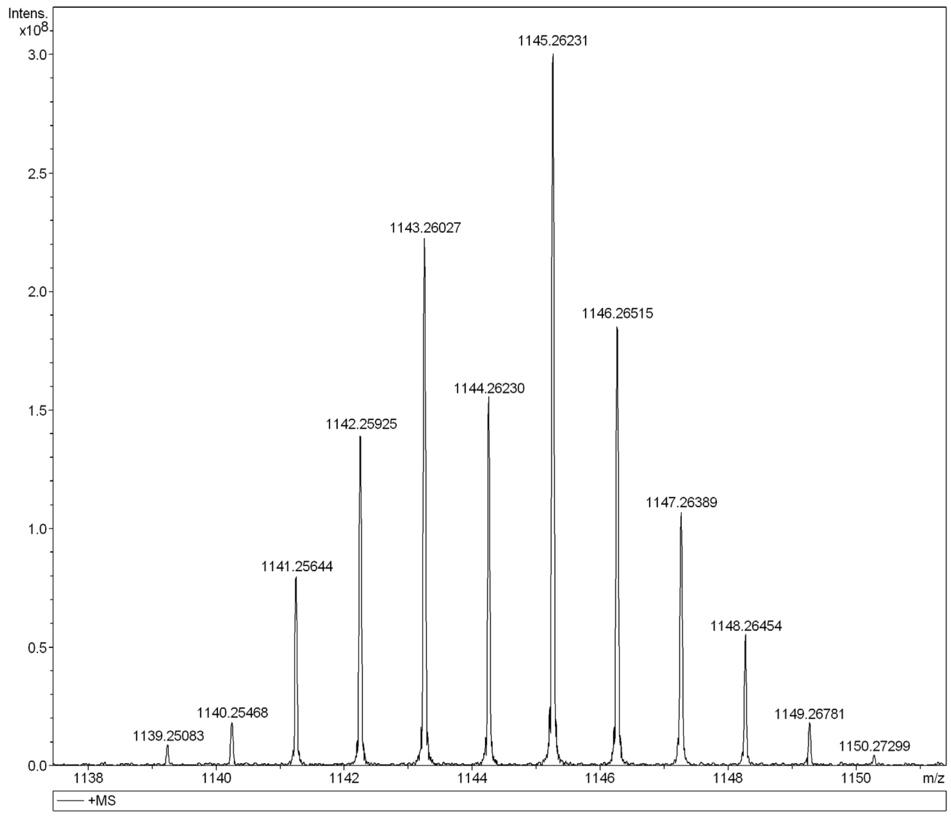

Figure S11 The positive-ion ESI-MS spectrum of [**1**]$^+$ measured in dichloromethane.

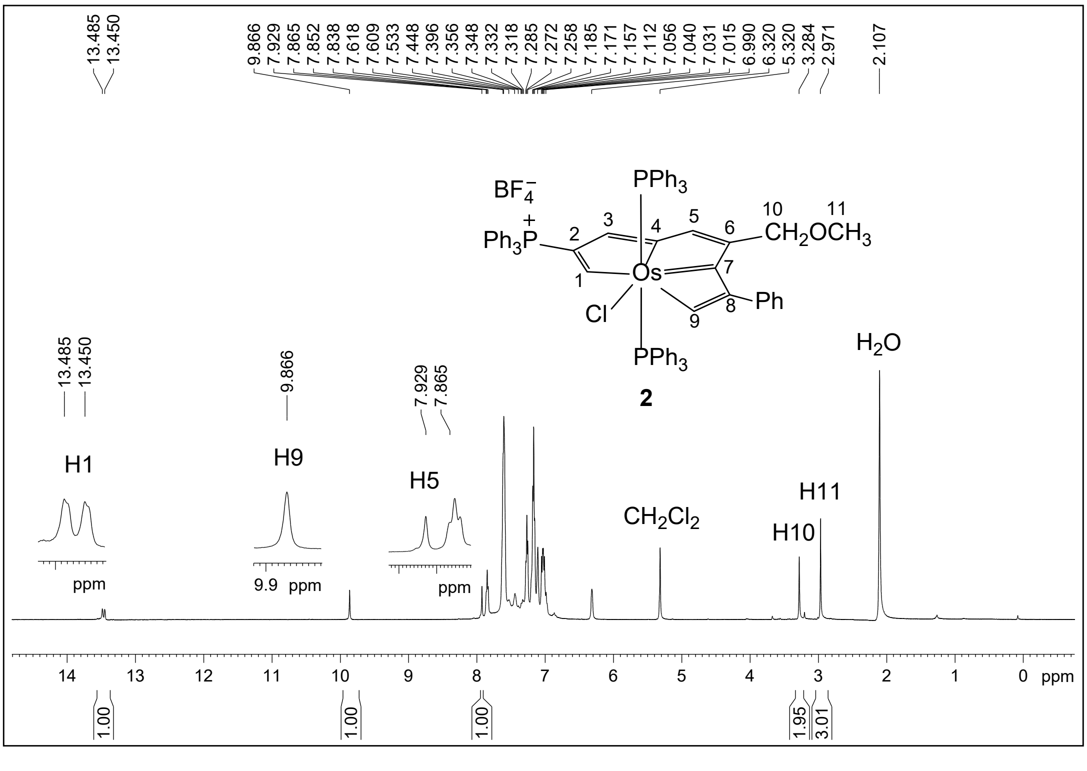

Figure S12 The ^{1}H NMR (500.2 MHz, CD_2Cl_2) spectrum for complex **2**.

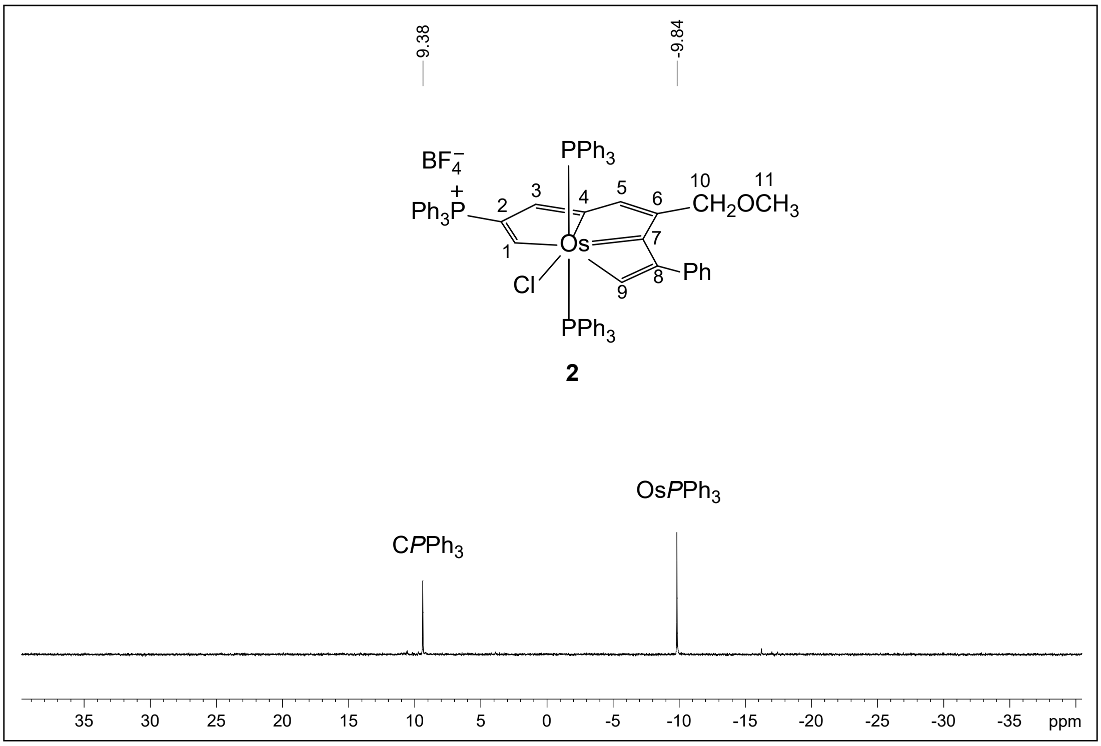

Figure S13 The $^{31}P\{^1H\}$ NMR (202.5 MHz, CD_2Cl_2) spectrum for complex **2**.

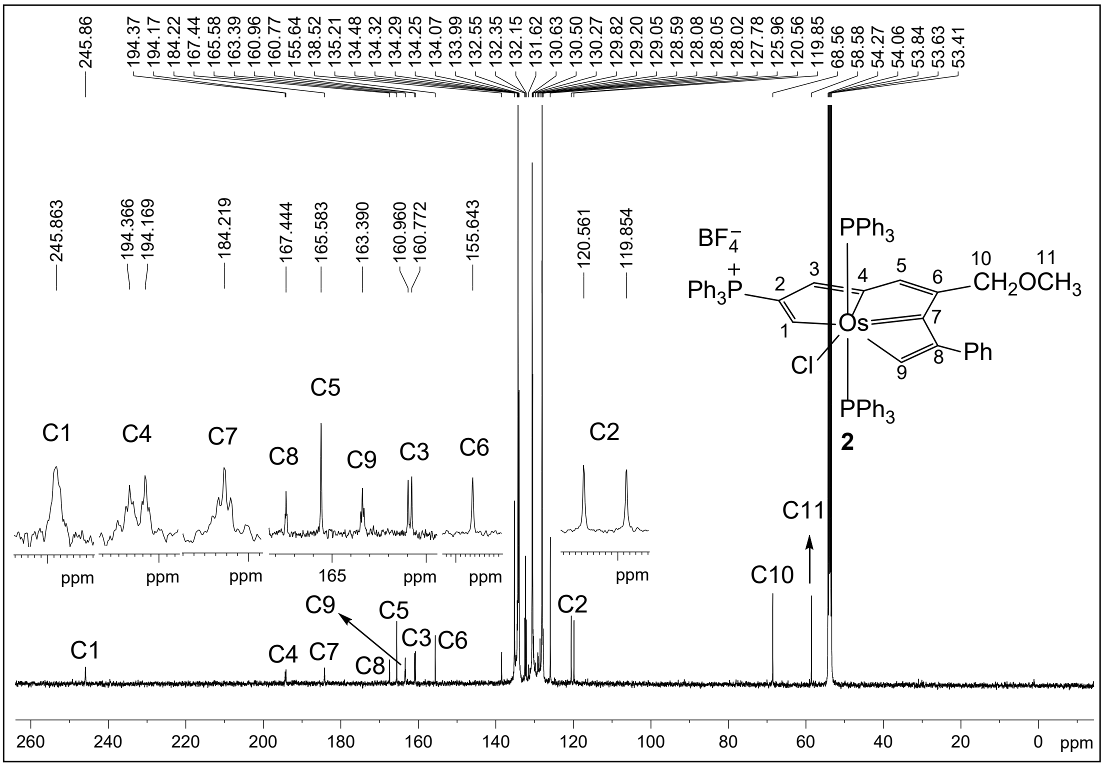

Figure S14 The $^{13}C\{^1H\}$ NMR (125.8 MHz, CD_2Cl_2) spectrum for complex **2**.

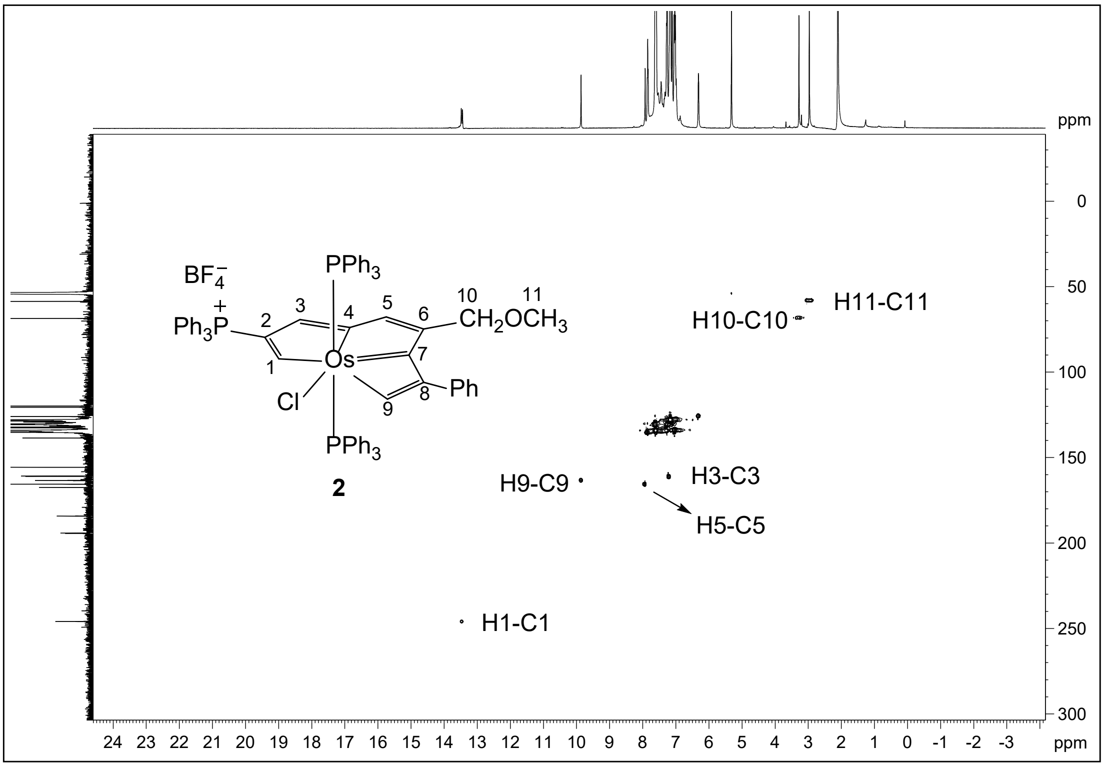

Figure S15 The two-dimensional ^{1}H-^{13}C HSQC spectrum for complex **2** in CD_2Cl_2.

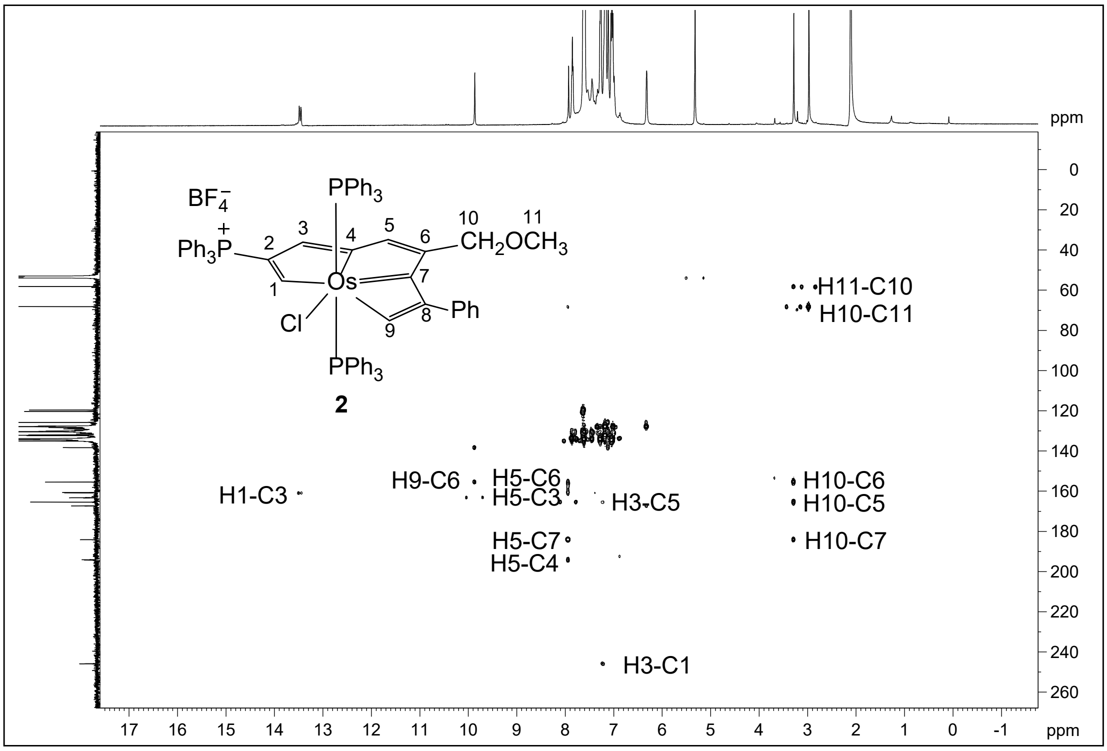

Figure S16 The two-dimensional ^{1}H-^{13}C HMBC spectrum for complex **2** in CD_2Cl_2.

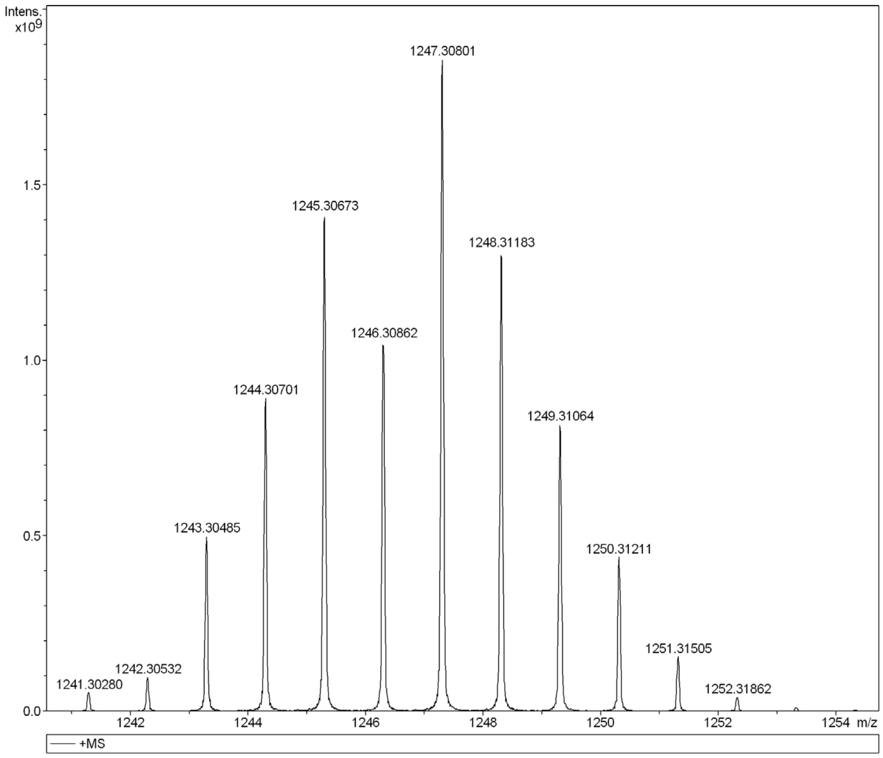

Figure S17 The positive-ion ESI-MS spectrum of [**2**]$^+$ measured in dichloromethane.

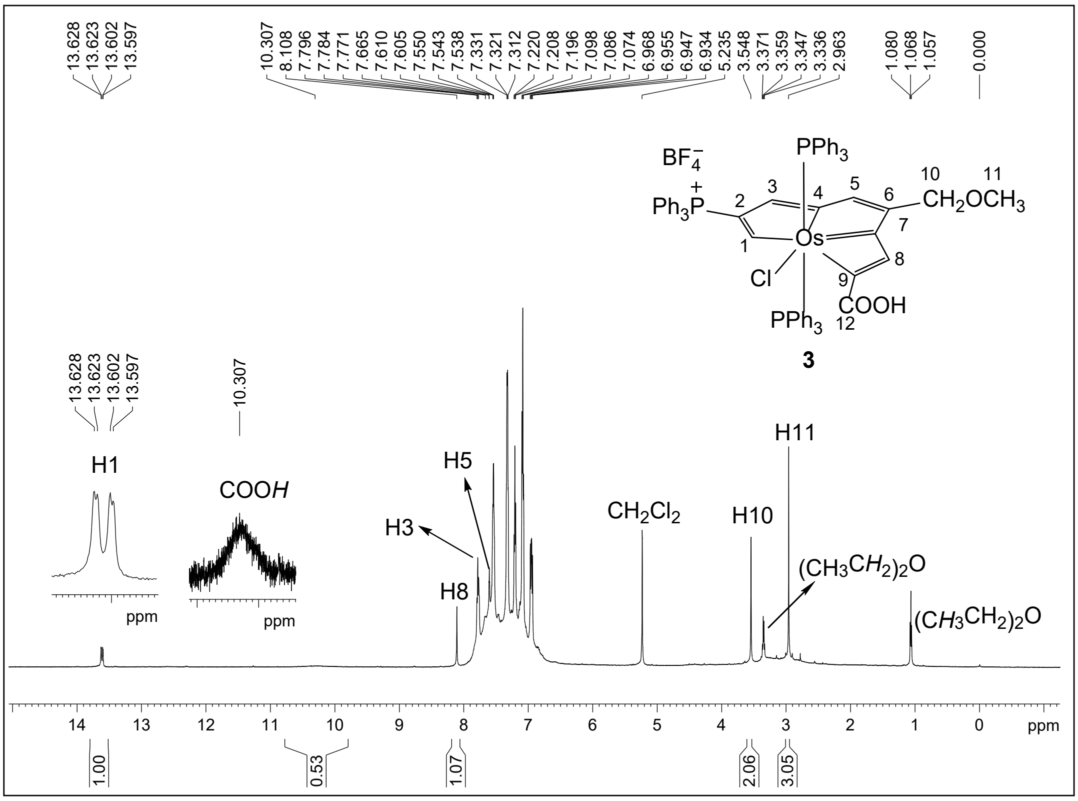

Figure S18 The ^{1}H NMR (600.1 MHz, CD_2Cl_2) spectrum for complex **3**.

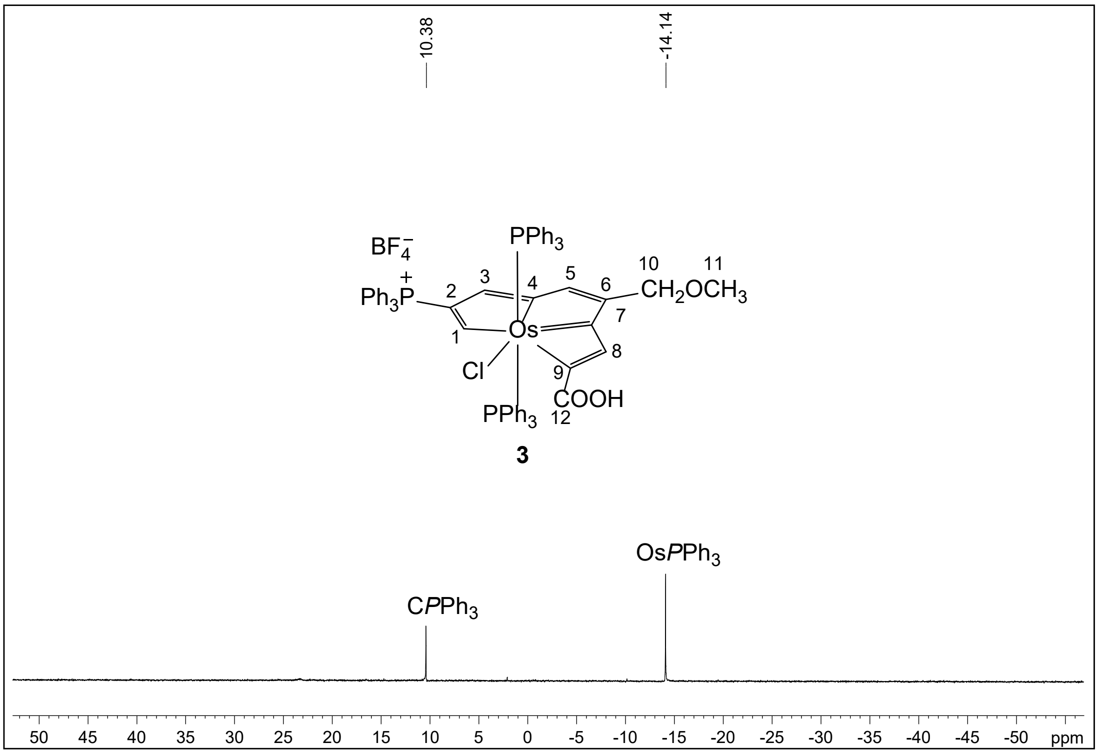

Figure S19 The $^{31}P\{^{1}H\}$ NMR (242.9 MHz, CD_2Cl_2) spectrum for complex **3**.

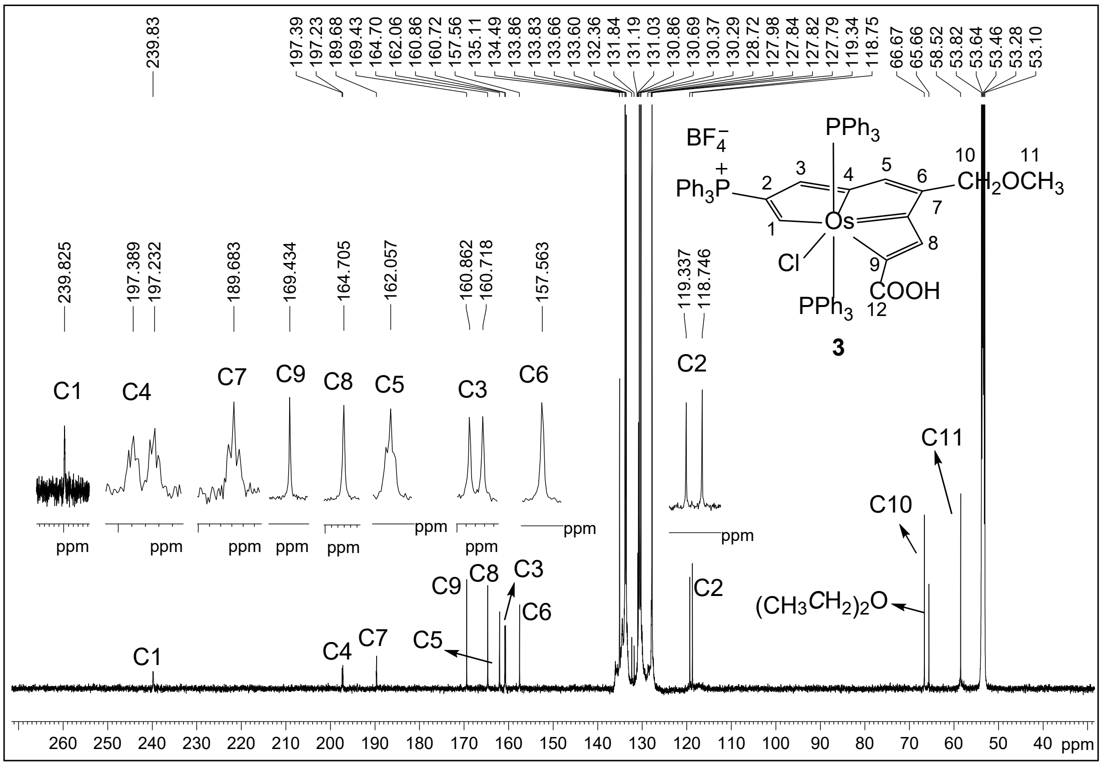

Figure S20 The $^{13}C\{^{1}H\}$ NMR (150.9 MHz, CD_2Cl_2) spectrum for complex **3**.

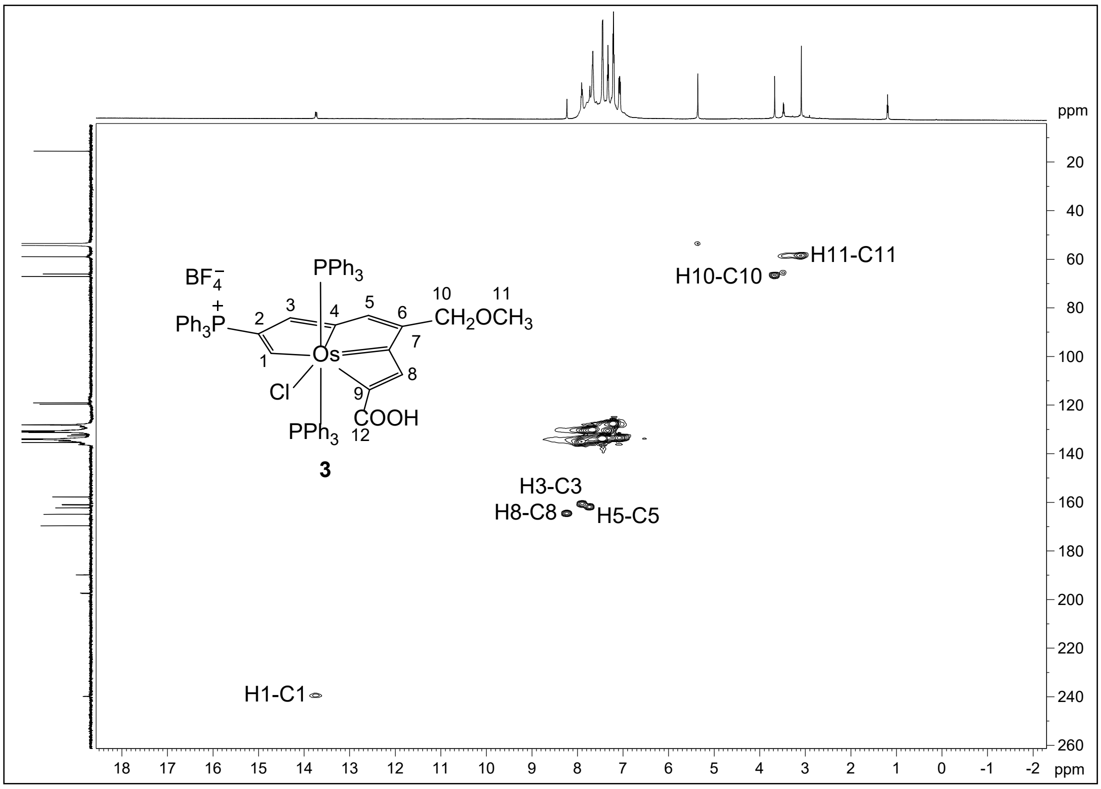

Figure S21 The two-dimensional ^{1}H-^{13}C HSQC spectrum for complex **3** in CD_2Cl_2.

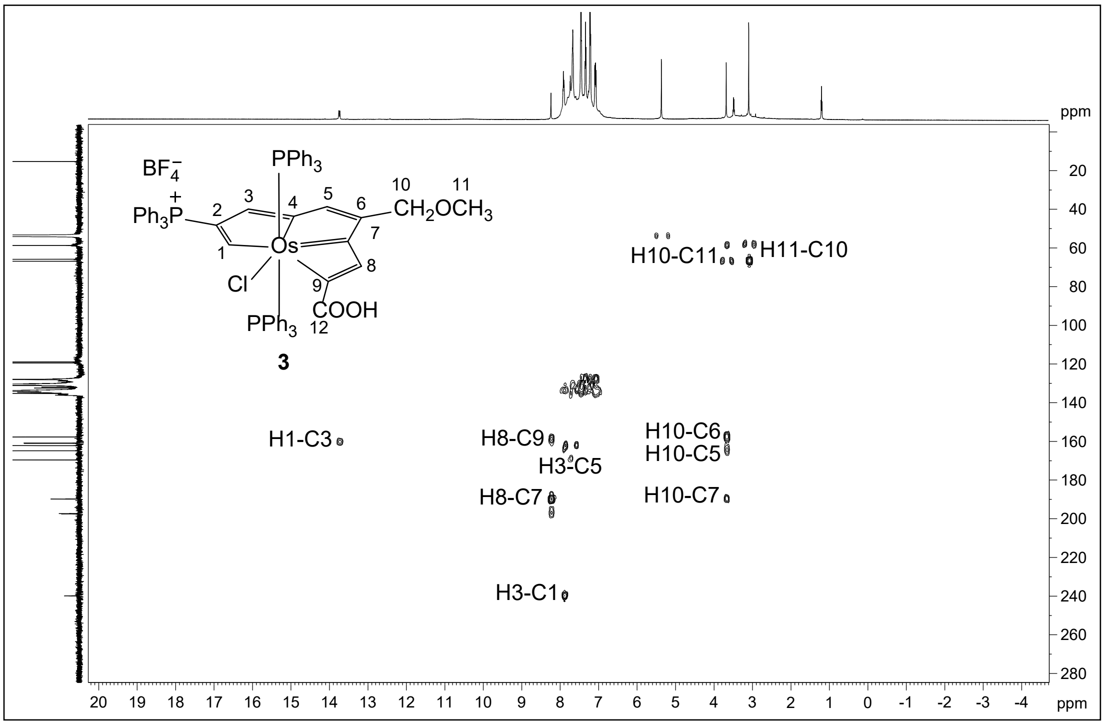

Figure S22 The two-dimensional ^{1}H-^{13}C HMBC spectrum for complex **3** in CD_2Cl_2.

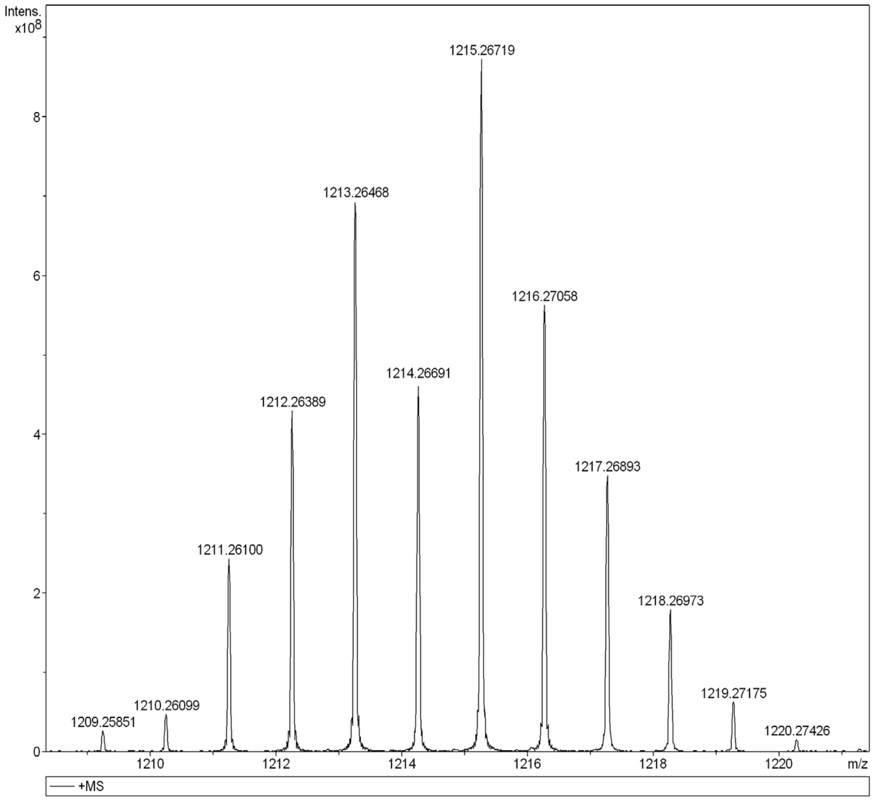

Figure S23 The positive-ion ESI-MS spectrum of $[\mathbf{3}]^+$ measured in dichloromethane.

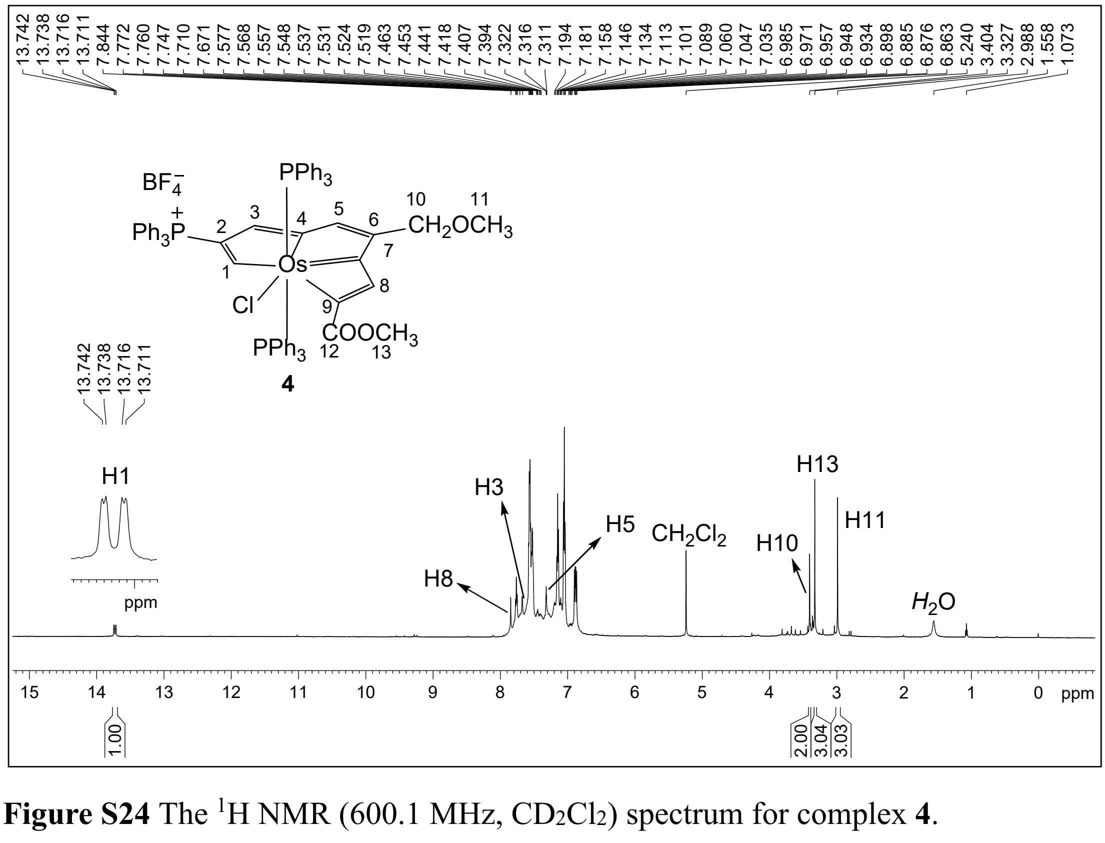

Figure S24 The ^{1}H NMR (600.1 MHz, CD_2Cl_2) spectrum for complex **4**.

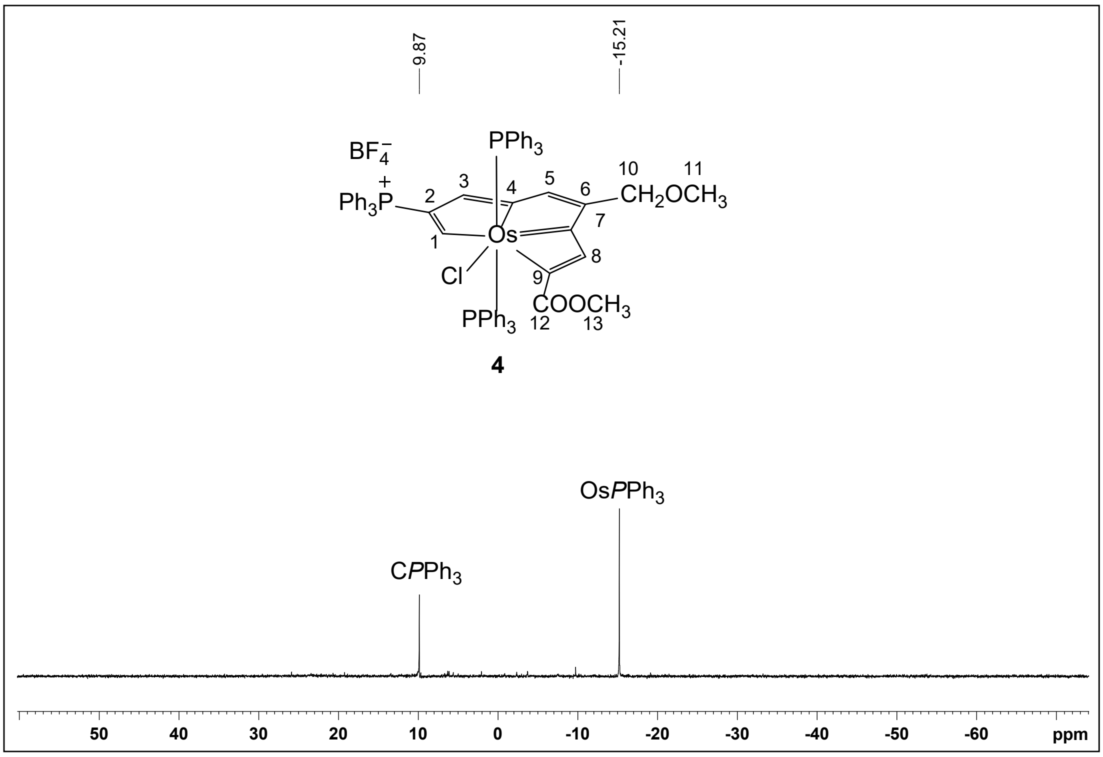

Figure S25 The ${}^{31}P\{{}^{1}H\}$ NMR (242.9 MHz, CD_2Cl_2) spectrum for complex **4**.

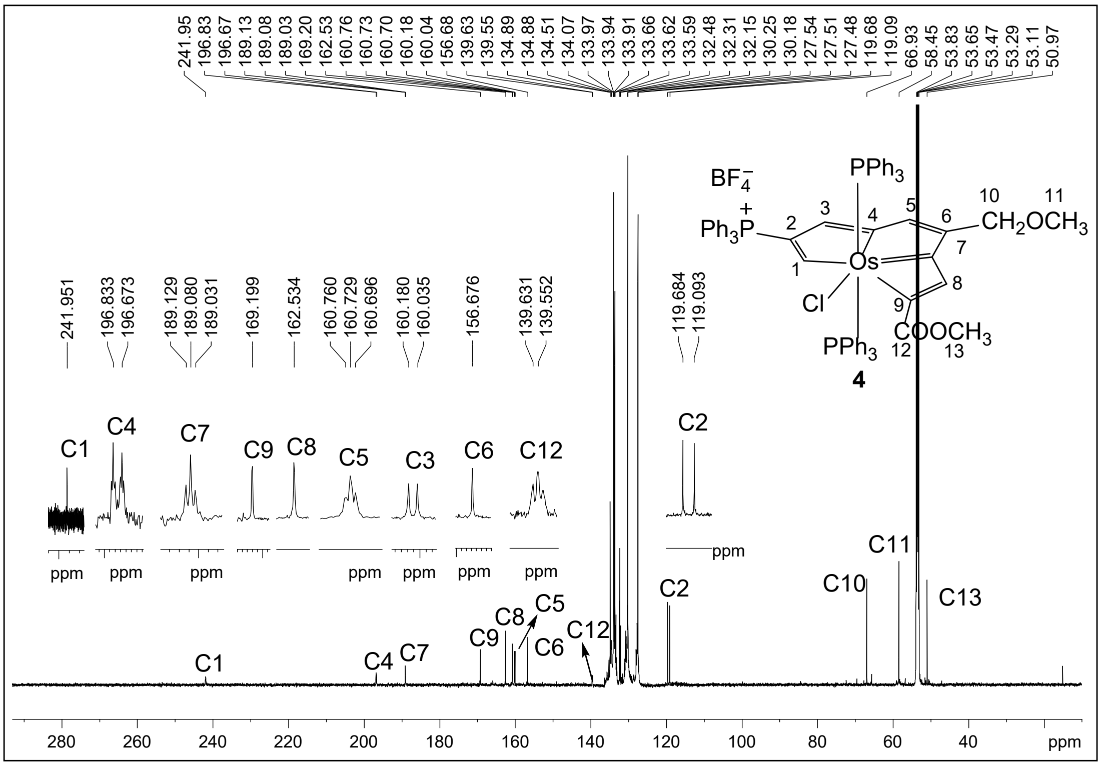

Figure S26 The ${}^{13}C\{{}^{1}H\}$ NMR (150.9 MHz, CD_2Cl_2) spectrum for complex **4**.

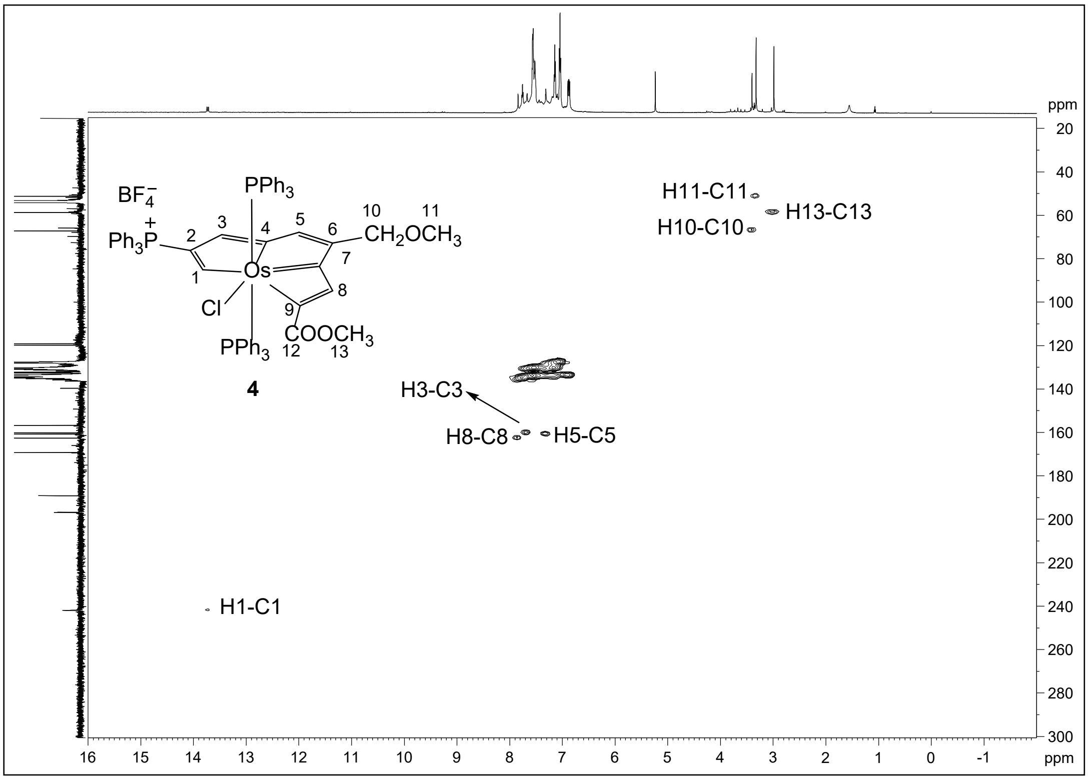

Figure S27 The two-dimensional ^{1}H-^{13}C HSQC spectrum for complex **4** in CD_2Cl_2.

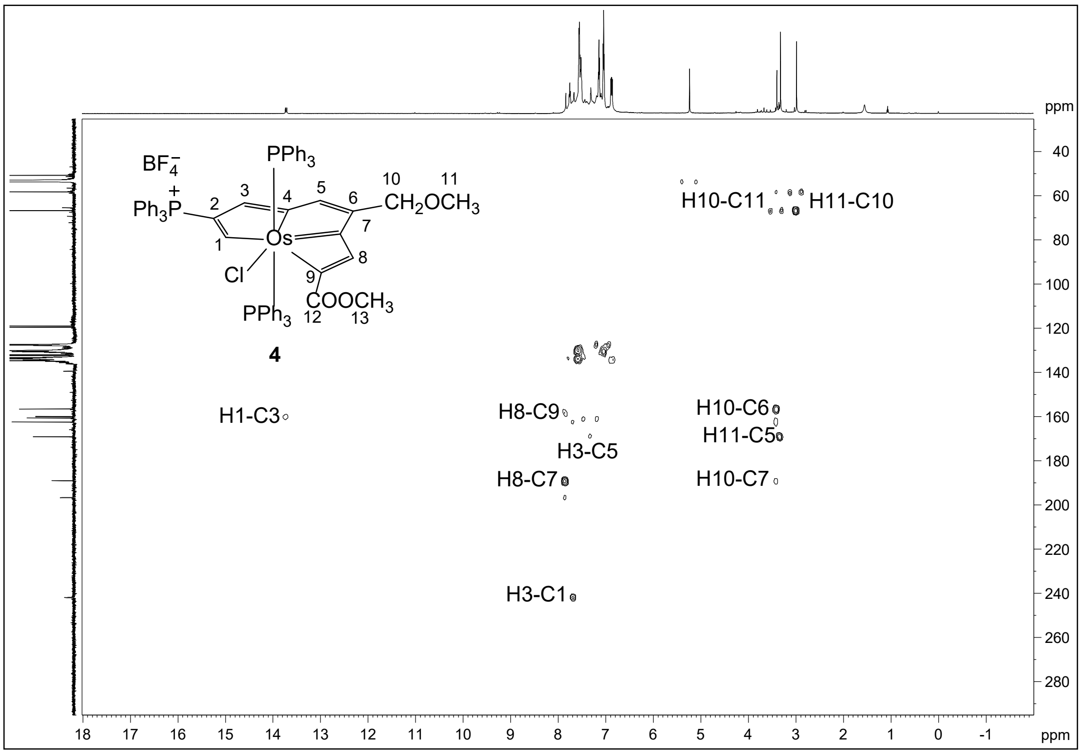

Figure S28 The two-dimensional ^{1}H-^{13}C HMBC spectrum for complex **4** in CD_2Cl_2.

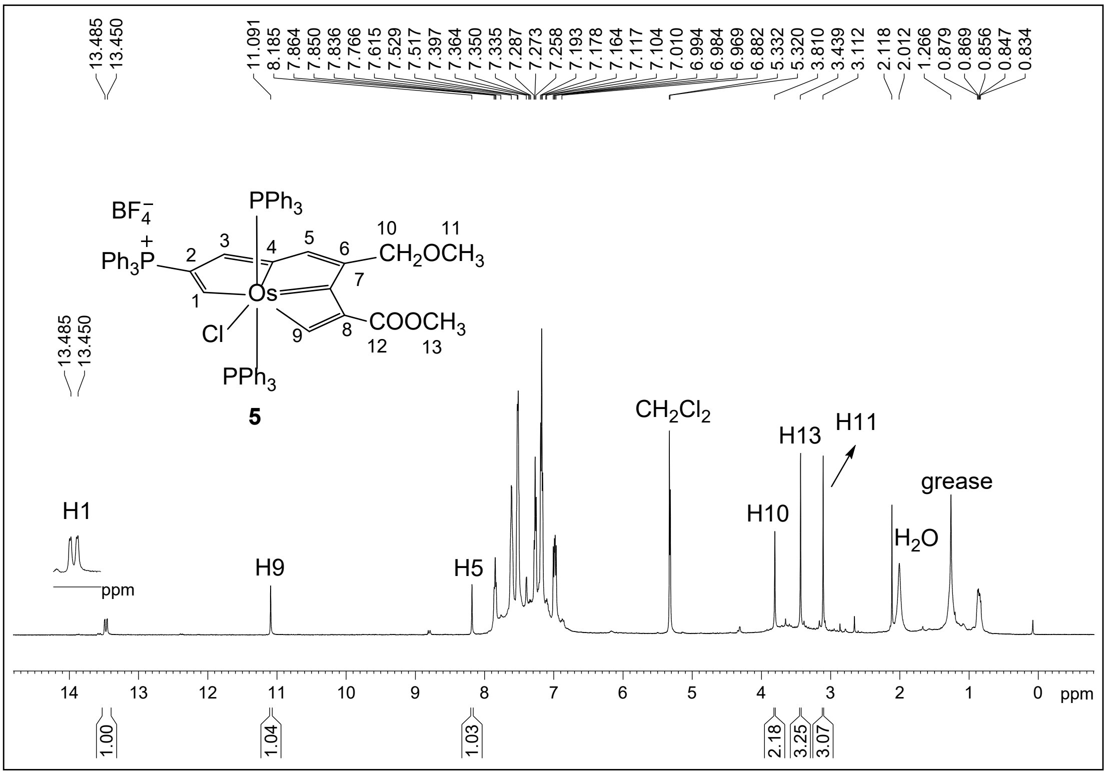

Figure S29 The ^{1}H NMR (500.2 MHz, CD_2Cl_2) spectrum for complex **5**.

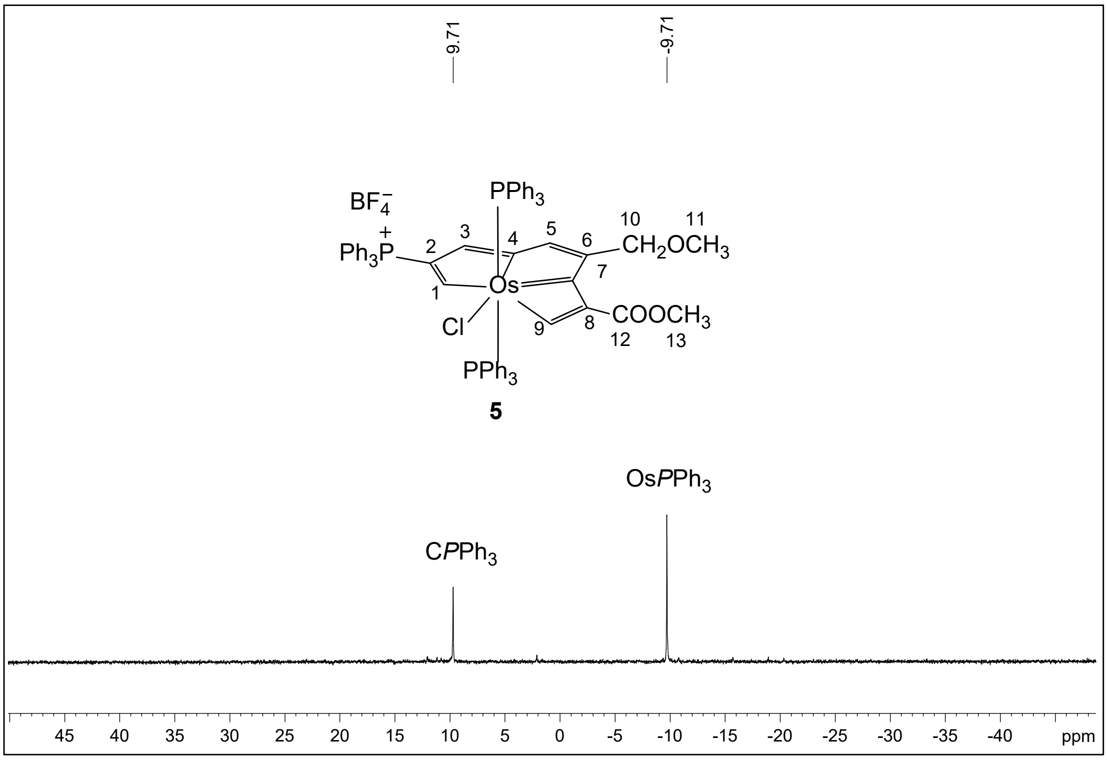

Figure S30 The ^{31}P{^{1}H} NMR (202.5 MHz, CD_2Cl_2) spectrum for complex **5**.

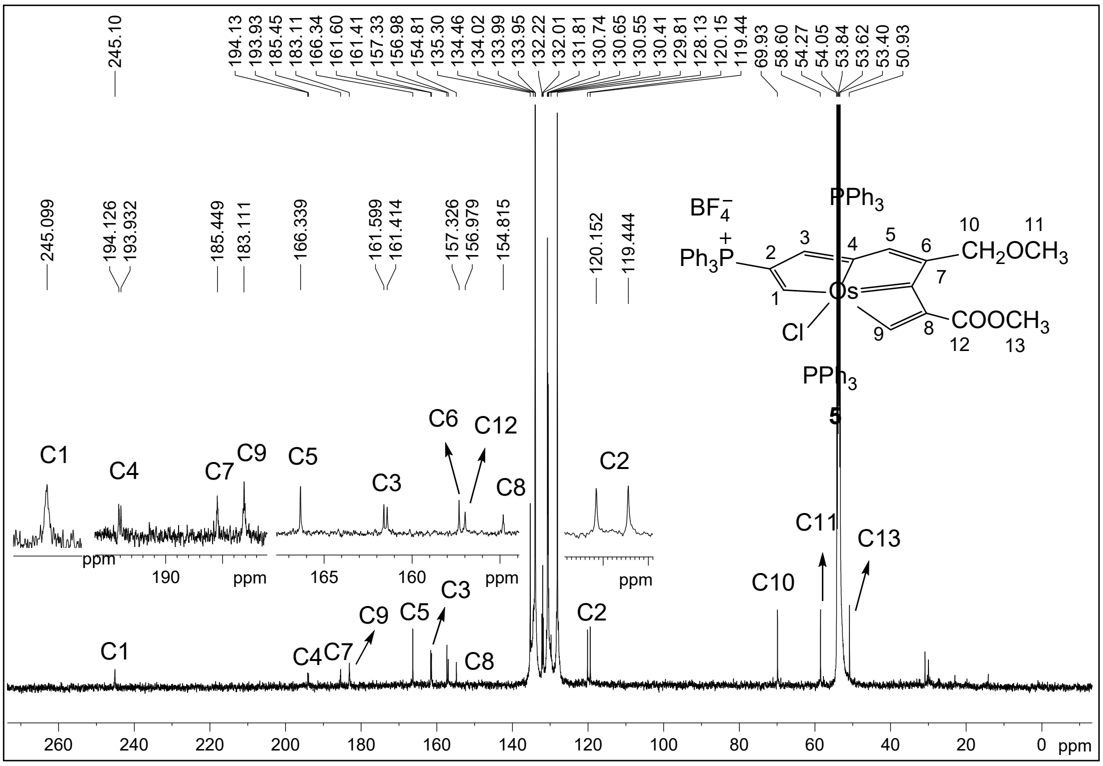

Figure S31 The $^{13}C\{^1H\}$ NMR (125.8 MHz, CD_2Cl_2) spectrum for complex **5**.

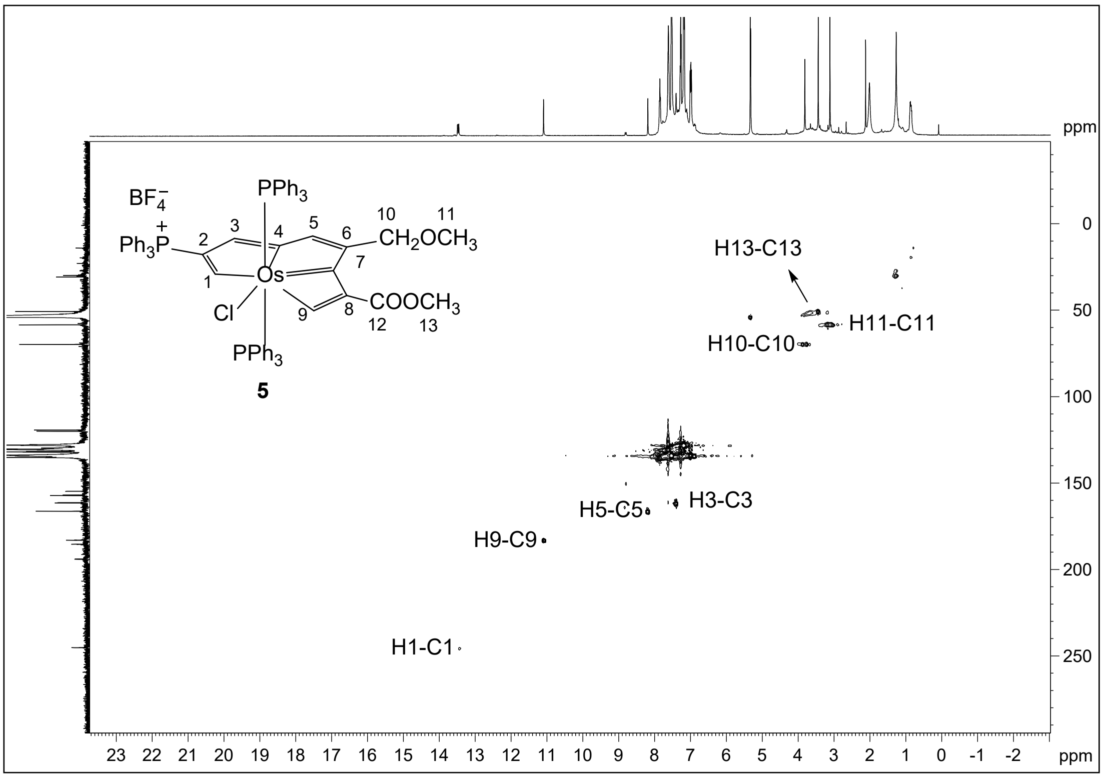

Figure S32 The two-dimensional ^1H-^{13}C HSQC spectrum for complex **5** in CD_2Cl_2.

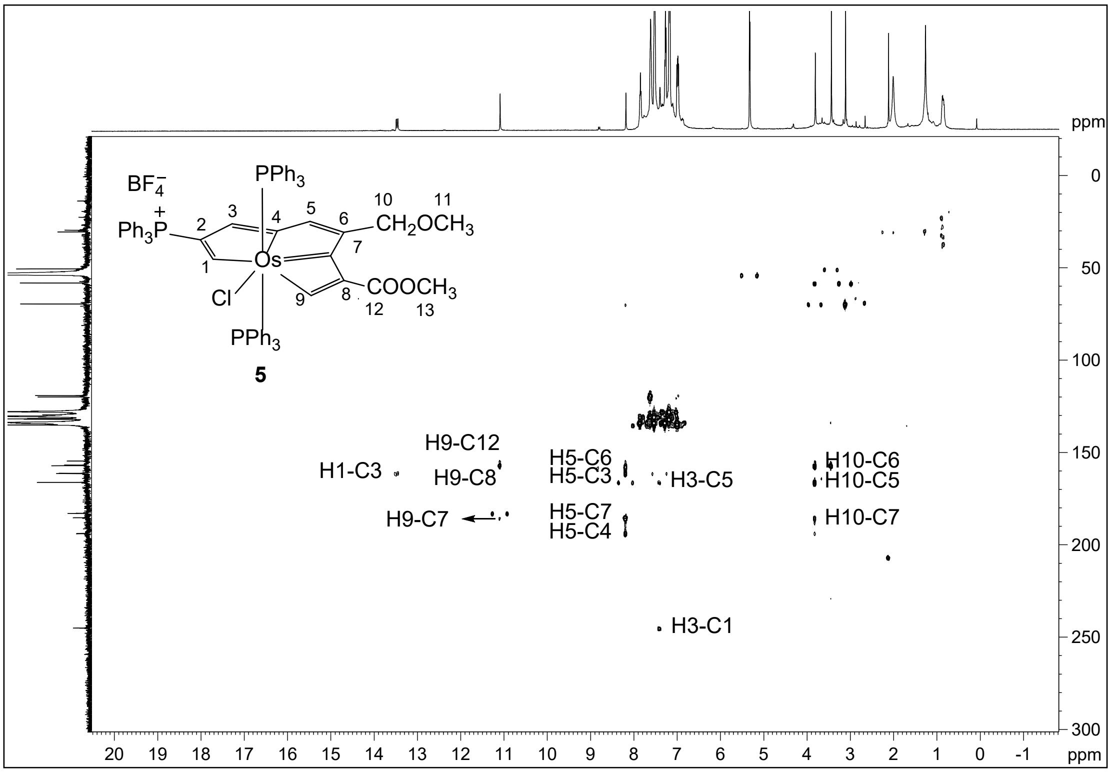

Figure S33 The two-dimensional ^{1}H-^{13}C HMBC spectrum for complex **5** in CD_2Cl_2.

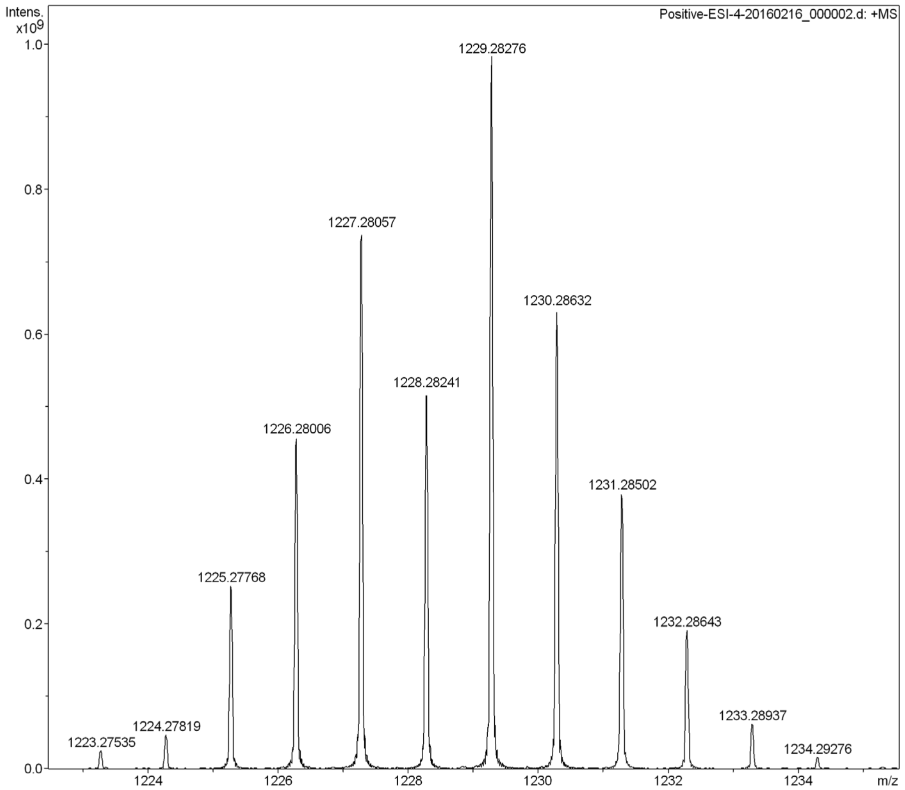

Figure S34 The positive-ion ESI-MS spectrum of $[\mathbf{5}]^+$ measured in dichloromethane.

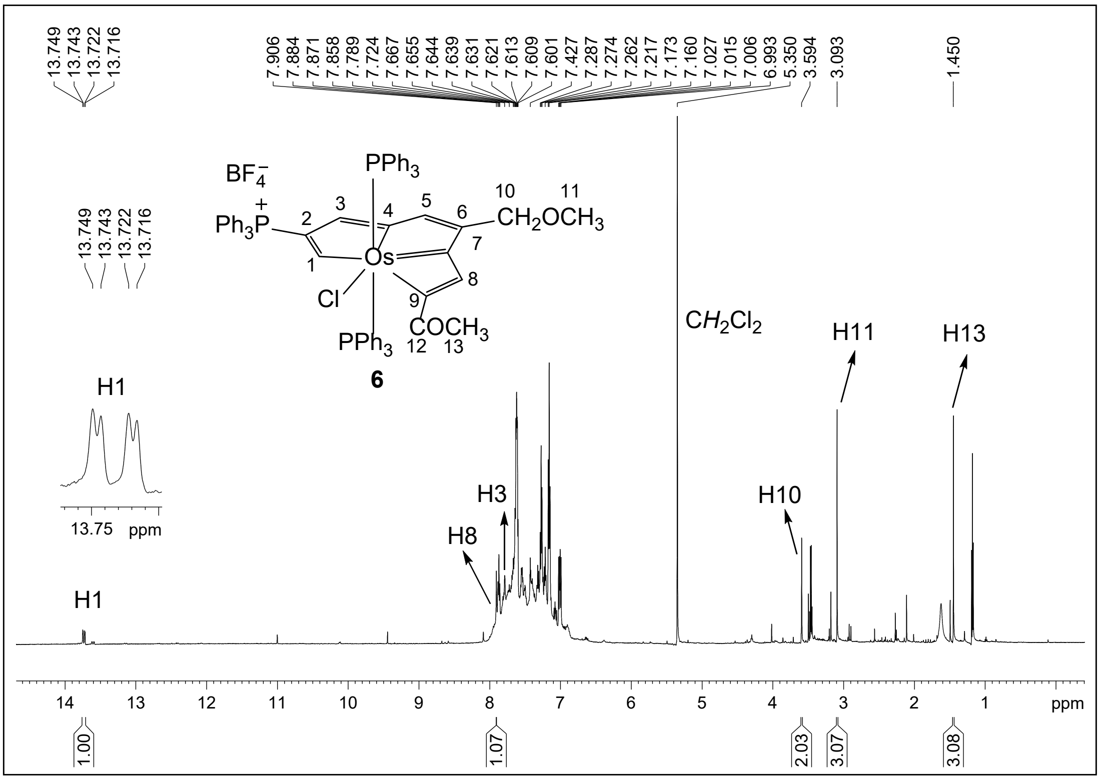

Figure S35 The ^{1}H NMR (600.1 MHz, CD_2Cl_2) spectrum for complex **6**.

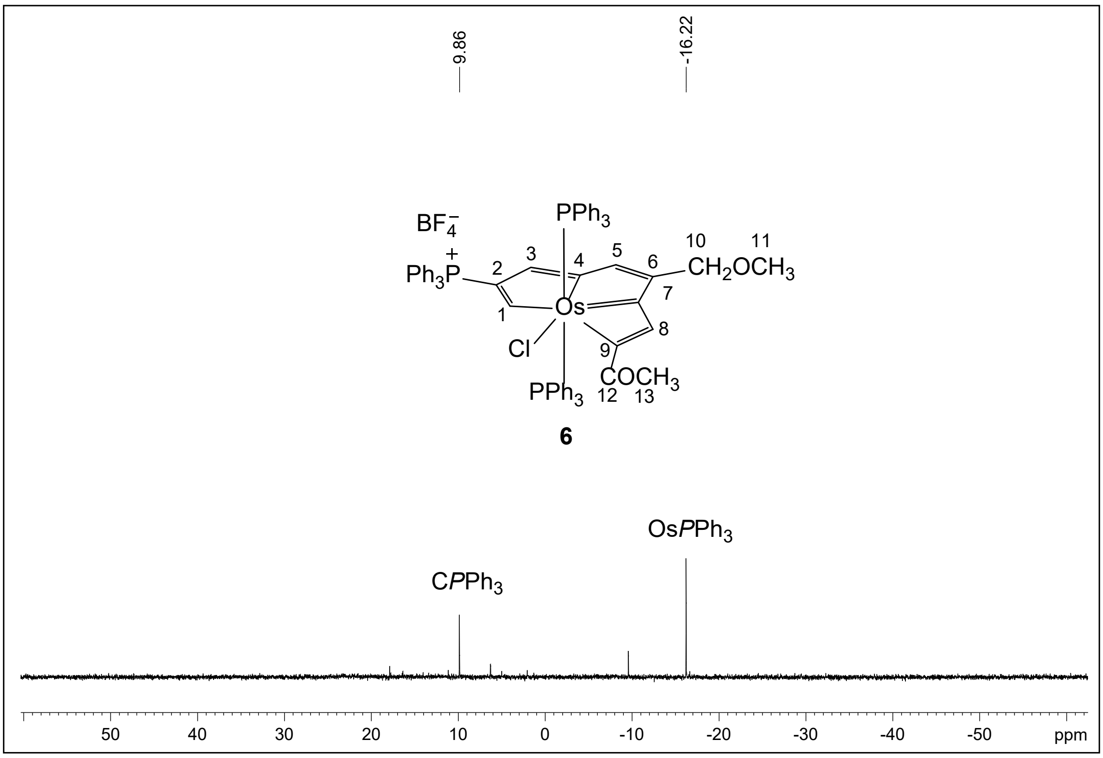

Figure S36 The $^{31}P\{^{1}H\}$ NMR (242.9 MHz, CD_2Cl_2) spectrum for complex **6**.

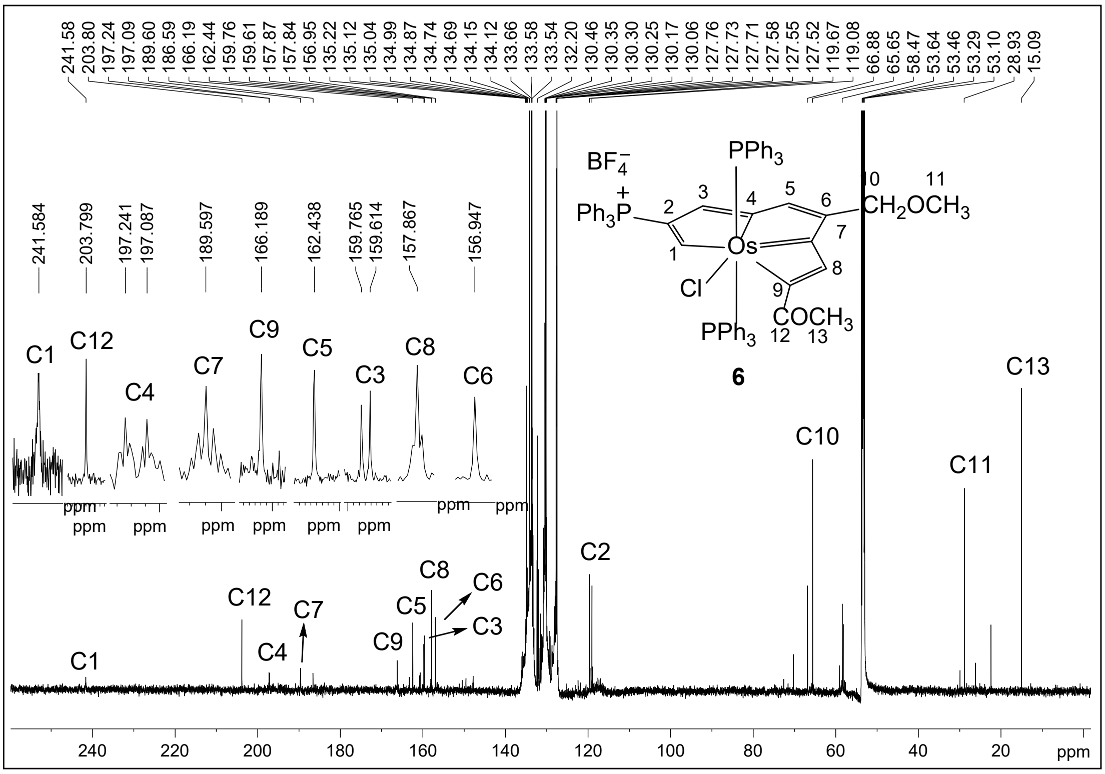

Figure S37 The $^{13}C\{^{1}H\}$ NMR (150.9 MHz, CD_2Cl_2) spectrum for complex **6**.

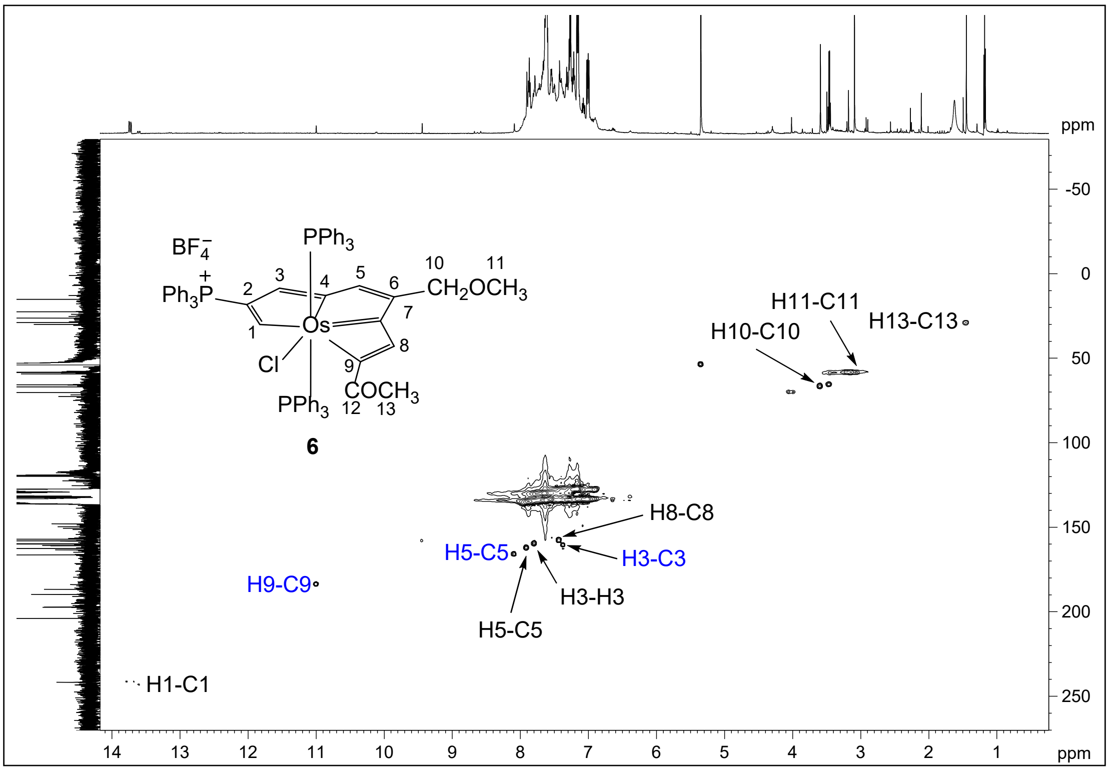

Figure S38 The two-dimensional ^{1}H-^{13}C HSQC spectrum for complex **6** in CD_2Cl_2.

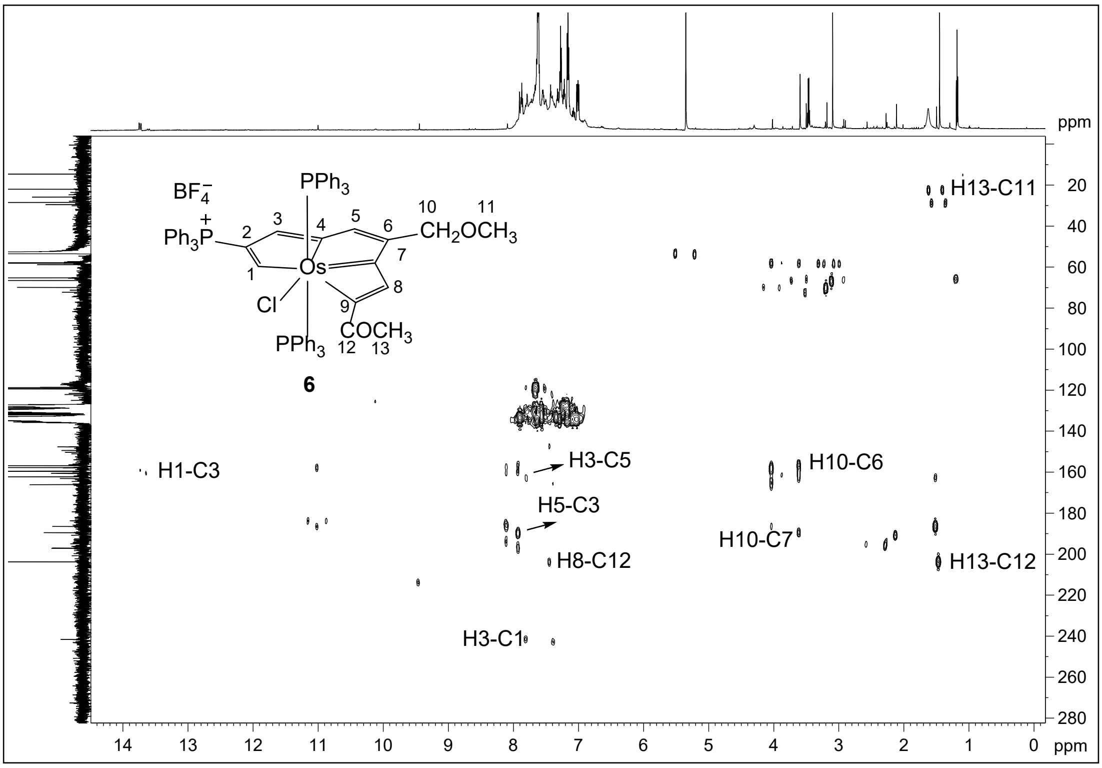

Figure S39 The two-dimensional ^{1}H-^{13}C HMBC spectrum for complex **6** in CD_2Cl_2.

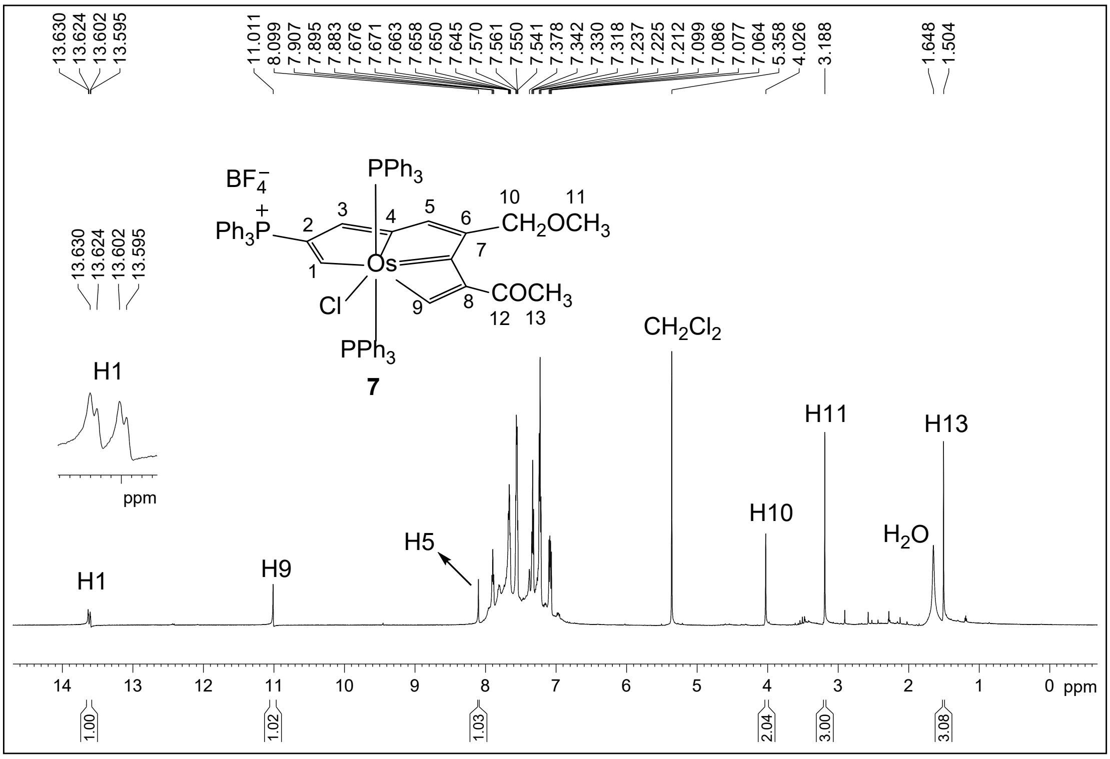

Figure S40 The ^{1}H NMR (600.1 MHz, CD_2Cl_2) spectrum for complex **7**.

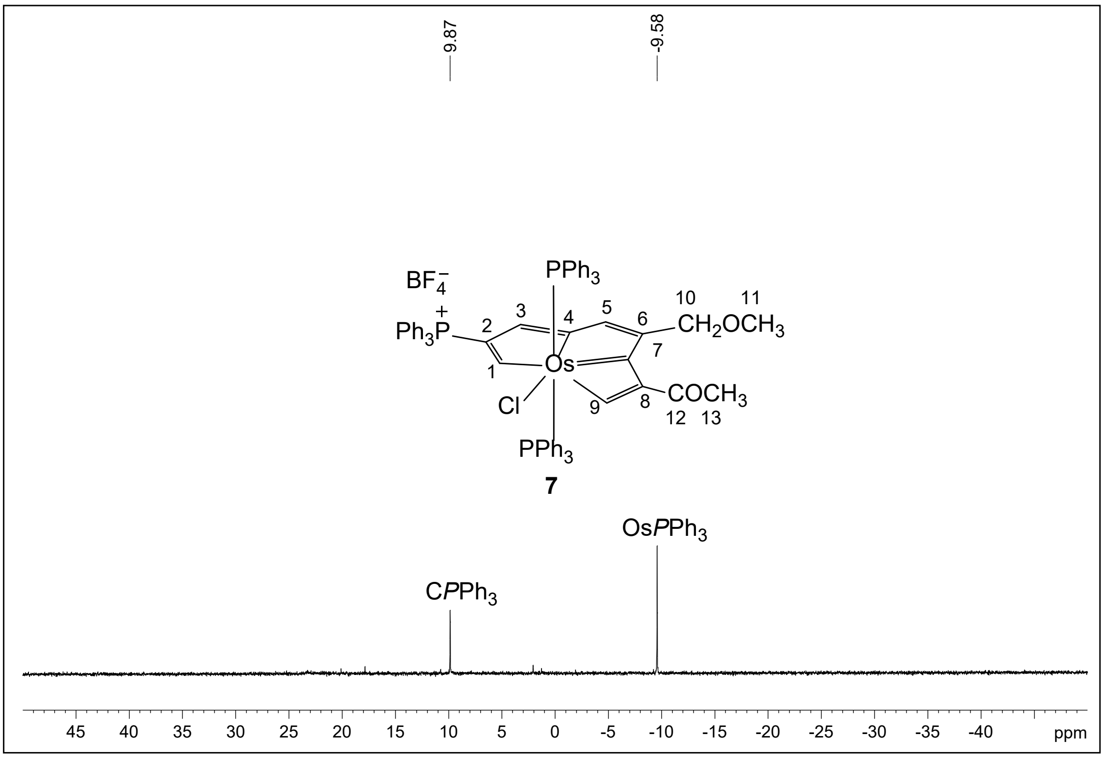

Figure S41 The $^{31}P\{^1H\}$ NMR (242.9 MHz, CD_2Cl_2) spectrum for complex **7**.

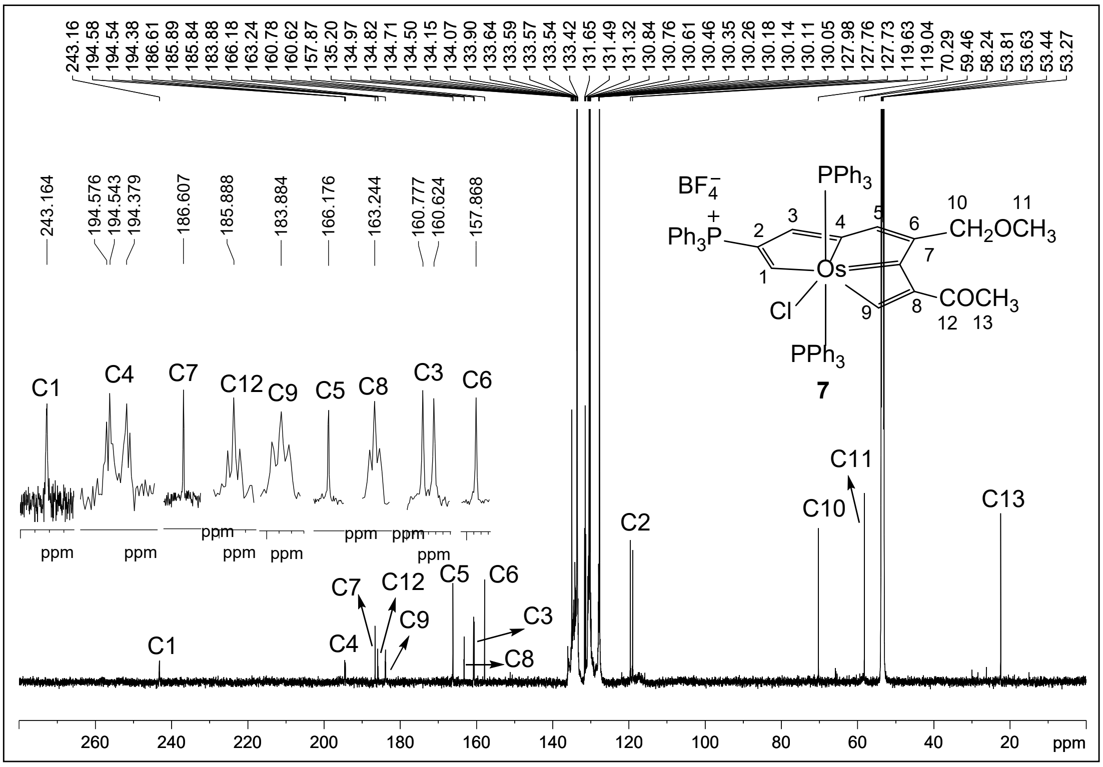

Figure S42 The $^{13}C\{^1H\}$ NMR (150.9 MHz, CD_2Cl_2) spectrum for complex **7**.

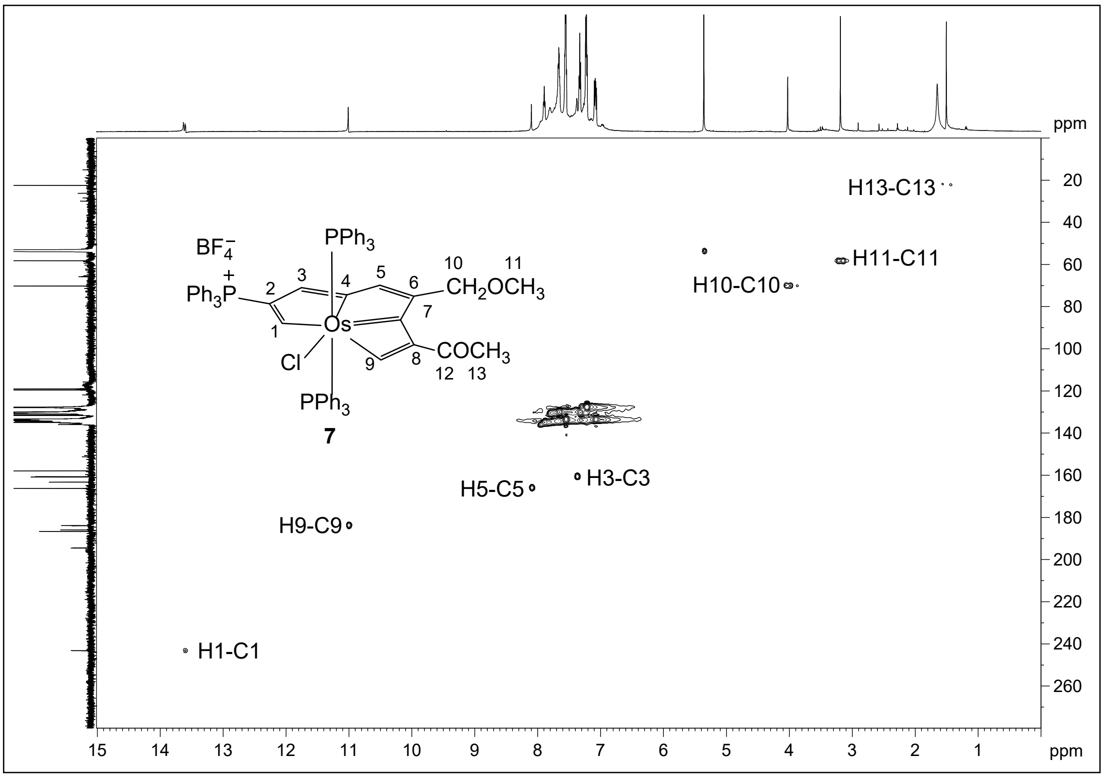

Figure S43 The two-dimensional ^{1}H-^{13}C HSQC spectrum for complex **7** in CD_2Cl_2.

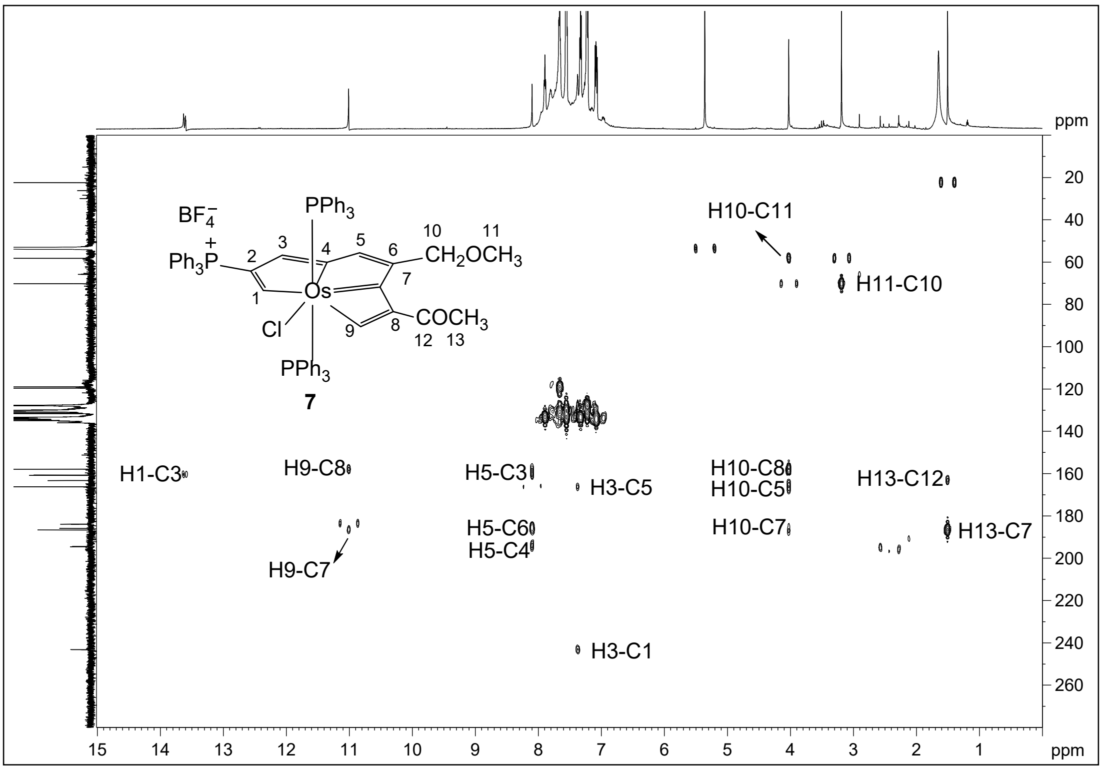

Figure S44 The two-dimensional ^{1}H-^{13}C HMBC spectrum for complex **7** in CD_2Cl_2.

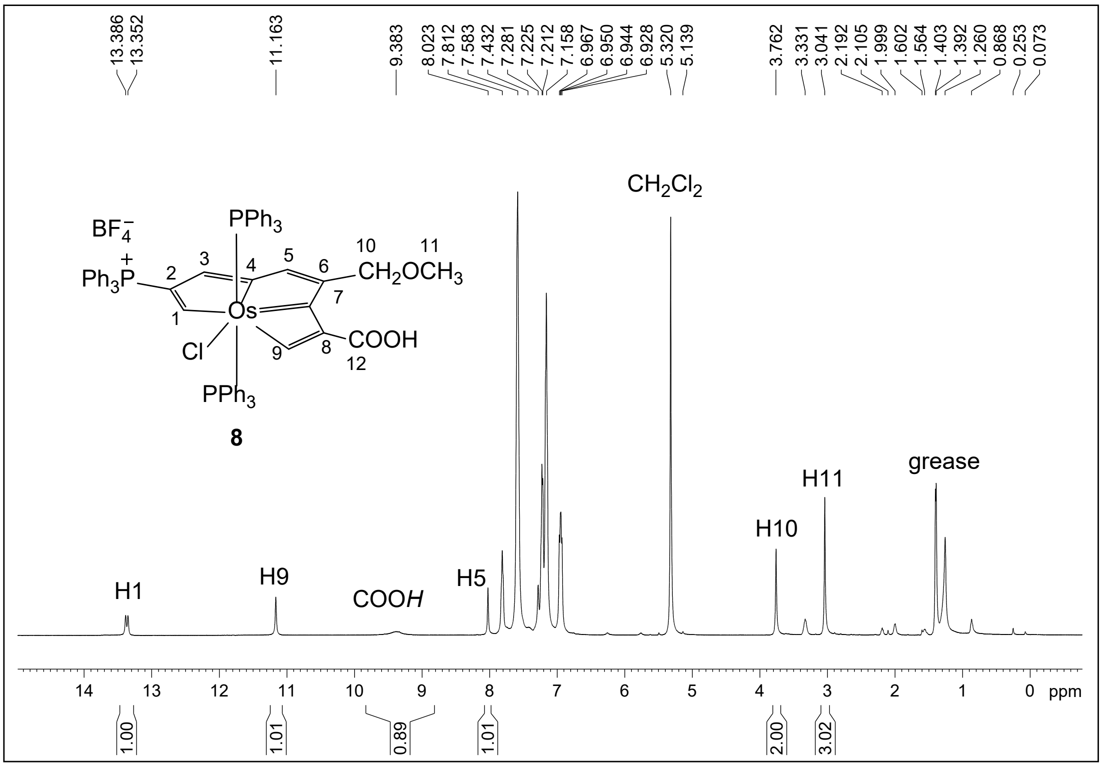

Figure S45 The ^{1}H NMR (500.2 MHz, CD_2Cl_2) spectrum for complex **8**.

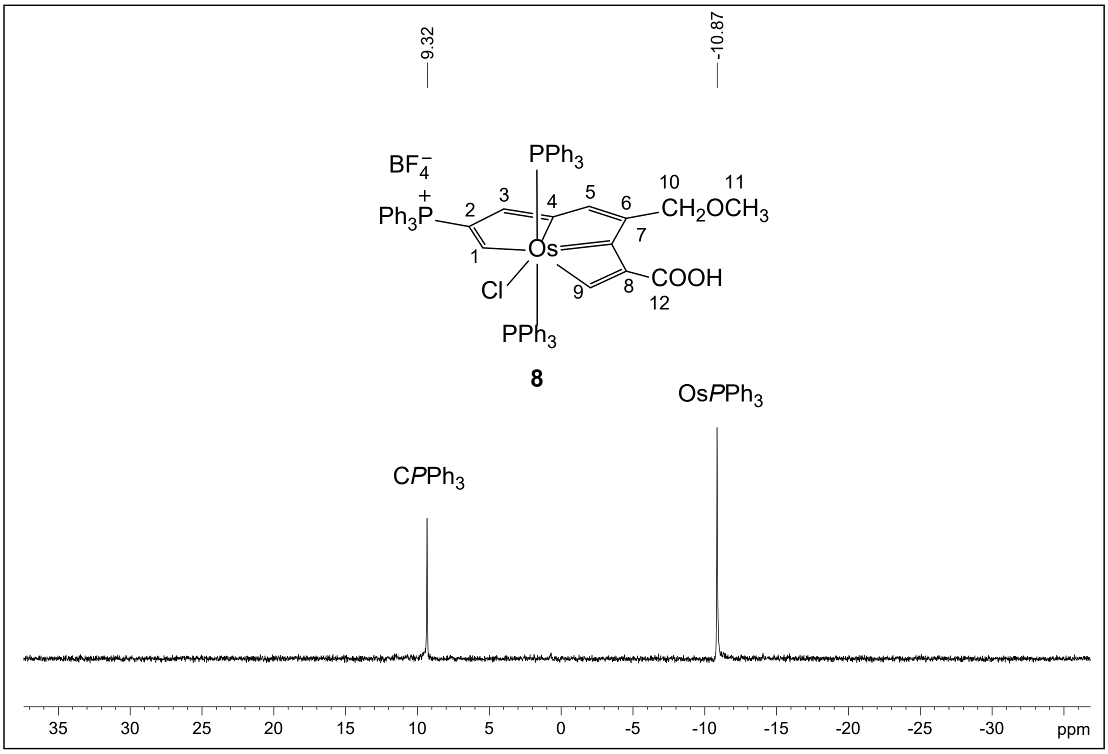

Figure S46 The $^{31}P\{^{1}H\}$ NMR (202.5 MHz, CD_2Cl_2) spectrum for complex **8**.

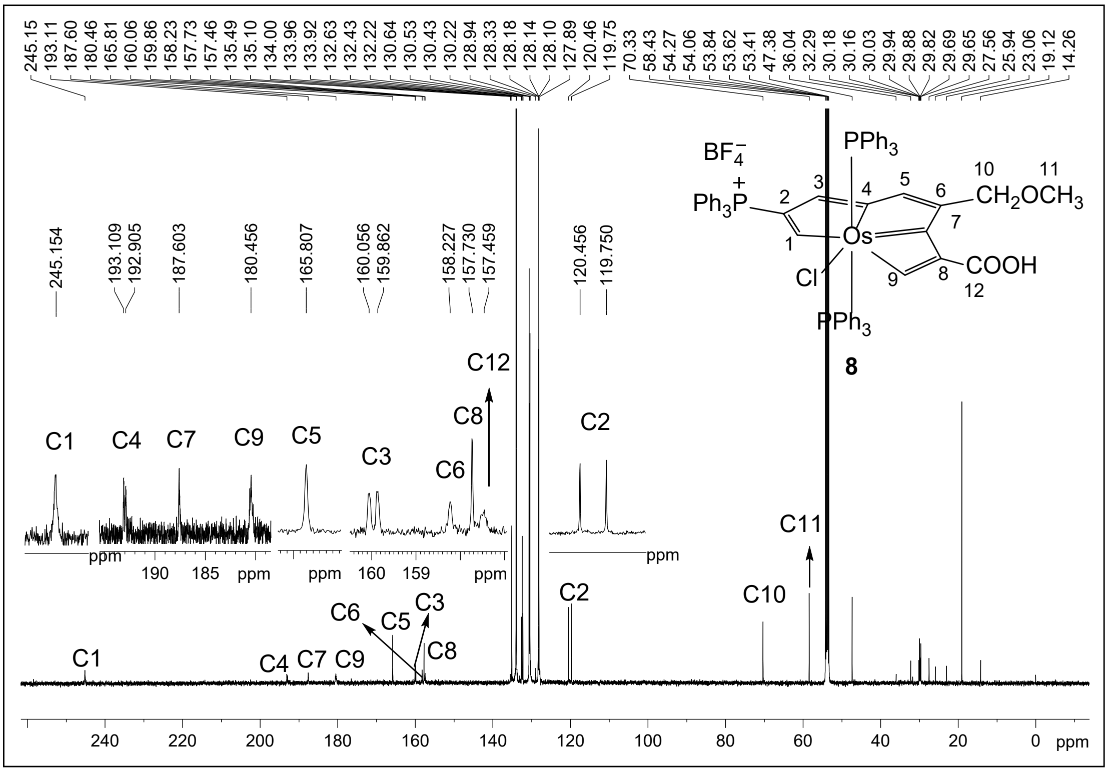

Figure S47 The $^{13}C\{^1H\}$ NMR (125.8 MHz, CD_2Cl_2) spectrum for complex **8**.

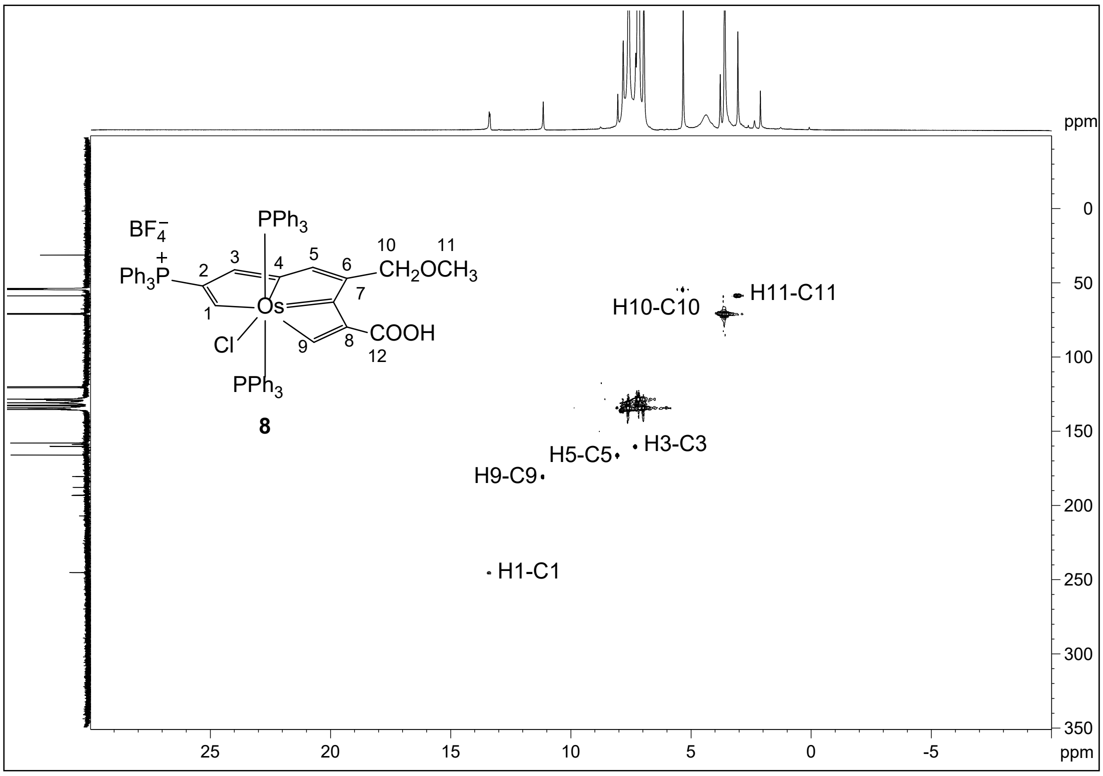

Figure S48 The two-dimensional ^1H-^{13}C HSQC spectrum for complex **8** in CD_2Cl_2.

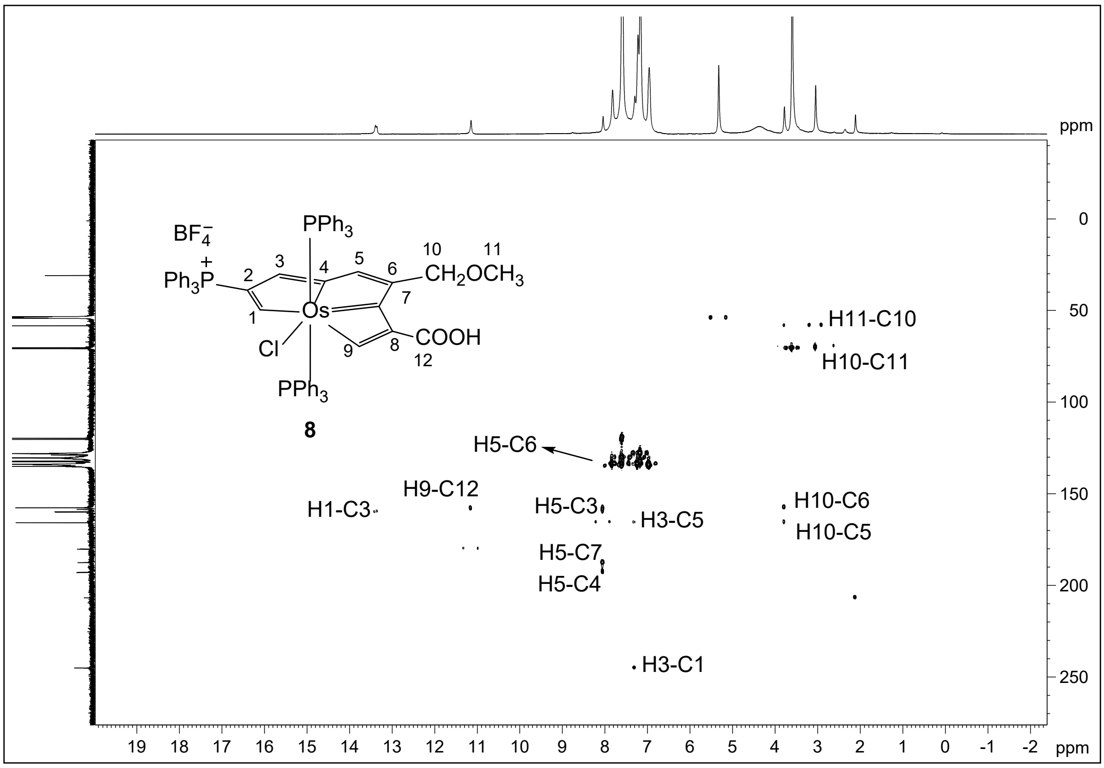

Figure S49 The two-dimensional ^{1}H-^{13}C HMBC spectrum for complex **8** in CD_2Cl_2.

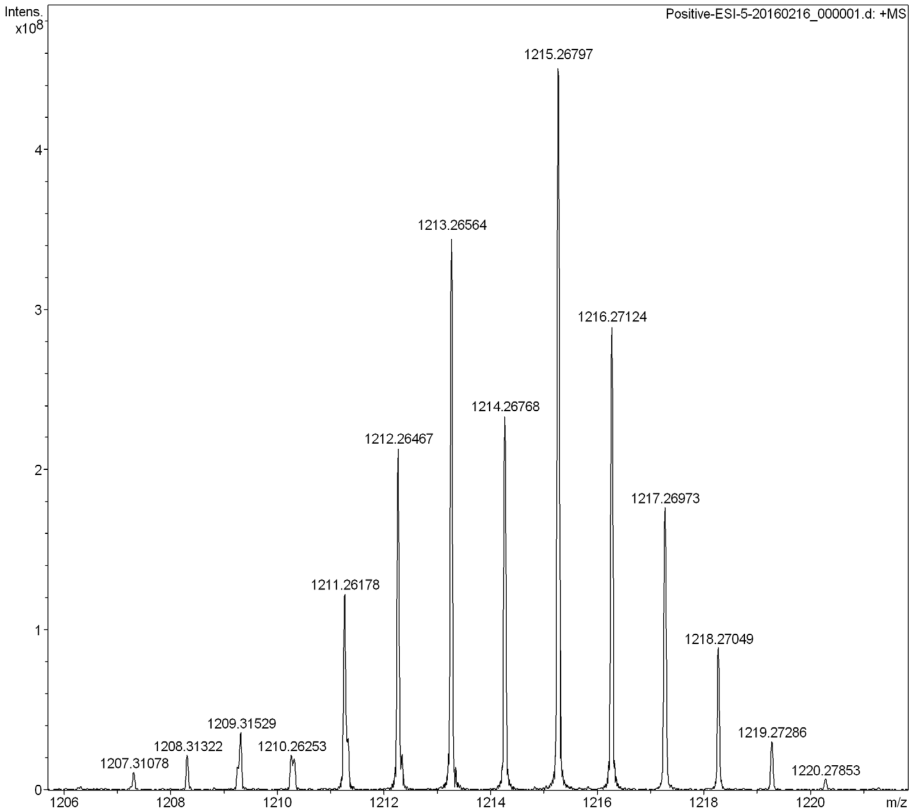

Figure S50 The positive-ion ESI-MS spectrum of [**8**]$^+$ measured in dichloromethane.

4. Crystallographic data

All single crystals suitable for X-ray diffraction were grown from dichloromethane solution layered with hexane. All single-crystal X-ray diffraction data were collected on a Rigaku XtaLAB Synergy, Dualflex, HyPix diffractometer with mirror-monochromated Cu Kα radiation (λ = 1.54184 Å). With Olex2,[3] the structures of **1**, **2**, **3** and **7** were solved with ShelXT[4] structure solution program using Direct methods and refined with the ShelXL[5] refinement package using Least Squares minimization. All non-hydrogen atoms were refined anisotropically. Hydrogen atoms were placed at idealized positions and assumed the riding model. Some of the solvent molecules and phenyl groups were disordered and refined with suitable restrains. Deposition Numbers 2203790 (for **1**), 2203791 (for **2**), 2203792 (for **3**), 2203793 (for **7**) contain the supplementary crystallographic data for this paper. These data are provided free of charge by the joint Cambridge Crystallographic Data Centre and Fachinformationszentrum Karlsruhe Access Structures service.

Table S3. Crystallographic details for complex **1**, **2**, **3** and **7**.

	**1**	**2**	**3**	**7**
Empirical formula	$C_{64}H_{55}BCl_3F_4OOsP_3$	$C_{71}H_{59}BClF_4OOsP_3$	$C_{66}H_{55}BClF_4O_3OsP_3$	$C_{69}H_{61}BCl_5F_4O_2OsP_3$
Formula weight	1316.35	1333.55	1301.47	1469.34
Temperature/K	100.0(3)	100.00(10)	99.9(5)	100.00(10)
Radiation	CuKα (1.54184)	CuKα (1.54184)	CuKα (1.54184)	CuKα (1.54184)
Crystal system	triclinic	triclinic	triclinic	orthorhombic
Space group	P-1	P-1	P-1	$P2_12_12_1$
a/Å	13.8621(3)	10.0898(2)	10.34490(10)	10.51270(10)
b/Å	15.1429(3)	13.4689(2)	12.07610(10)	15.31820(10)
c/Å	15.5533(2)	23.9566(3)	23.56430(10)	39.1267(4)
α/°	74.747(2)	78.3520(10)	94.5980(10)	90
β/°	77.875(2)	88.7400(10)	97.6210(10)	90
γ/°	70.274(2)	74.380(2)	106.2660(10)	90
V/Å^3	2938.44(11)	3069.05(9)	2779.63(4)	6300.79(10)
Z	2	2	2	4
d_{calc}/g cm^{-3}	1.488	1.443	1.555	1.549
μ/mm^{-1}	6.567	5.516	6.101	6.966
F(000)	1320.0	1344.0	1308.0	2952.0
Reflections collected	76047	28254	74112	60677
Independent reflections	9971	9819	11030	10715
Data/restraints/parameters	9971/6/695	9819/0/740	11030/0/714	10715/36/768
Goodness-of-fit on F_2	1.063	1.079	1.054	1.190
Final R indexes [I>=2σ(I)]	R_1 = 0.0513, wR_2 = 0.1455	R_1 = 0.0356, wR_2 = 0.0924	R_1 = 0.0304, wR_2 = 0.0769	R_1 = 0.0304, wR_2 = 0.0726
peak/hole/e Å^{-3}	4.04/-1.62	1.26/-1.67	1.37/-1.53	0.95/-0.91

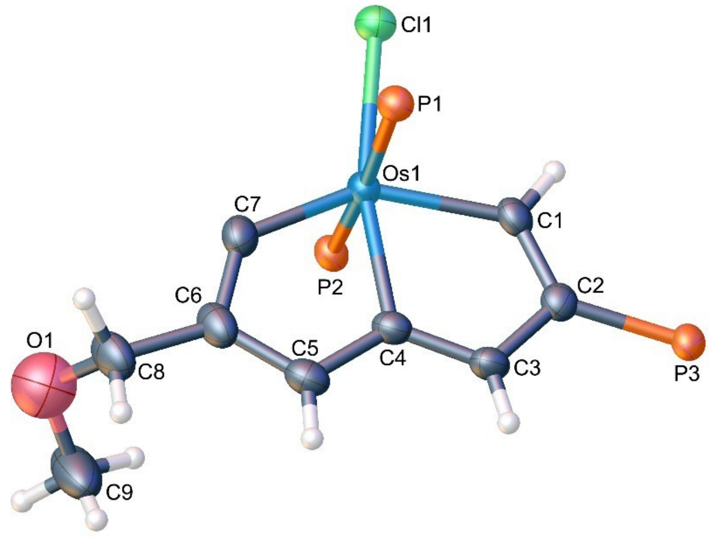

Figure S51 X-ray molecular structure for the cation of complex **1** drawn with 50% probability level. The phenyl groups in PPh_3 groups are omitted for clarity. Selected bond lengths [Å] and angles [°]: Os1-C1 2.034(5), Os1-C4 2.101(5), Os1-C7 1.870(6), C1-C2 1.368(8), C2-C3 1.428(8), C3-C4 1.364(8), C4-C5 1.428(8), C5-C6 1.372(9), C6-C7 1.410(9), Cl1-Os1 2.4338(13), C2-P3 1.793(6), Os1-C1-C2 120.9(4), C1-C2-C3 113.0(5), C2-C3-C4 113.3(5), C3-C4-Os1 118.4(4), C4-Os1-C1 72.4(2), Os1-C4-C5 117.2(4), C4-C5-C6 113.0(5), C5-C6-C7 107.2(5), C6-C7-Os1 130.5(5), C7-Os1-C4 72.2(2).

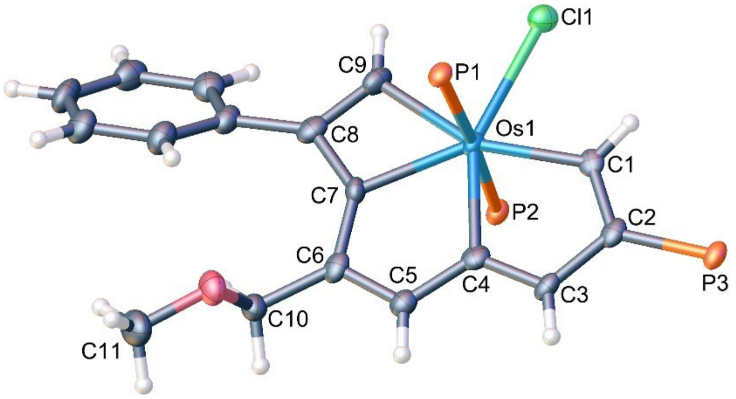

Figure S52 X-ray molecular structure for the cation of complex **2** drawn with 50% probability level. The phenyl groups in PPh_3 groups are omitted for clarity. Selected bond lengths [Å] and angles [°]: Os1-C1 2.070(4), Os1-C4 2.099(4), Os1-C7 2.086(4), Os1-C9 2.143(4), C1-C2 1.377(5), C2-C3 1.415(6), C3-C4 1.377(5), C4-C5 1.405(6), C5-C6 1.361(5), C6-C7 1.404(6), C7-C8 1.440(5), C8-C9 1.355(6), Os1-Cl1 2.4907(9), C2-P3 1.781(4), Os1-C1-C2 121.5(3), C1-C2-C3 112.3(3), C2-C3-C4 113.3(3), C3-C4-Os1 119.8(3), C4-Os1-C1 73.07(15), Os1-C4-C5 119.2(3), C4-C5-C6 115.6(3), C5-C6-C7 110.6(4), C6-C7-Os1 122.5(3), C7-Os1-C4 72.13(15), Os1-C7-C8 102.2(3), C7-C8-C9 96.3(3), C8-C9-Os1 102.5(3), C9-Os1-C7 58.98(15).

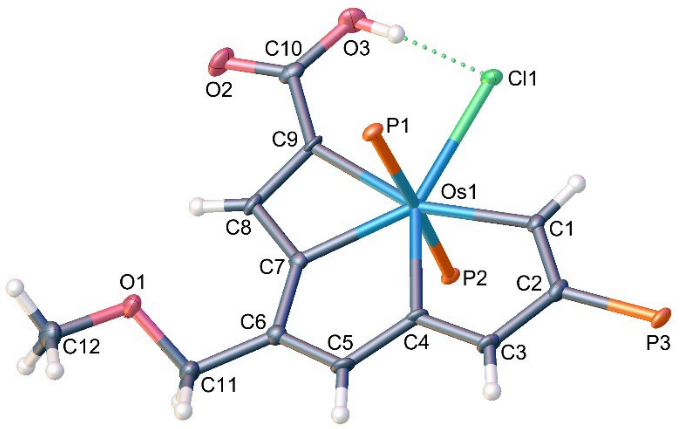

Figure S53 X-ray molecular structure for the cation of complex **3** drawn with 50% probability level. The phenyl groups in PPh_3 groups are omitted for clarity. Selected bond lengths [Å] and angles [°]: Os1-C1 2.076(3), Os1-C4 2.111(2), Os1-C7 2.063(3), Os1-C9 2.232(3), C1-C2 1.381(4), C2-C3 1.416(4), C3-C4 1.387(4), C4-C5 1.410(4), C5-C6 1.366(4), C6-C7 1.400(4), C7-C8 1.421(4), C8-C9 1.346(4), Os1-Cl1 2.4971(6), C2-P3 1.782(3), Os1-C1-C2 122.6(2), C1-C2-C3 111.9(2), C2-C3-C4 112.7(2), C3-C4-Os1 120.54(19), C4-Os1-C1 72.19(10), Os1-C4-C5 119.90(19), C4-C5-C6 114.2(2), C5-C6-C7 110.2(2), C6-C7-Os1 124.4(2), C7-Os1-C4 71.19(10), Os1-C7-C8 102.84(17), C7-C8-C9 100.4(2), C8-C9-Os1 97.51(17), C9-Os1-C7 59.22(10).

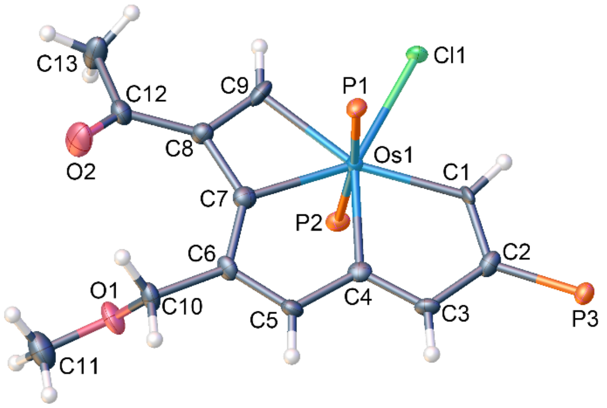

Figure S54 X-ray molecular structure for the cation of complex **7** drawn with 50% probability level. The phenyl groups in PPh_3 groups are omitted for clarity. Selected bond lengths [Å] and angles [°]: Os1-C1 2.089(6), Os1-C4 2.096(7), Os1-C7 2.089(7), Os1-C9 2.143(6), C1-C2 1.365(10), C2-C3 1.424(10), C3-C4 1.363(10), C4-C5 1.426(9), C5-C6 1.367(10), C6-C7 1.411(10), C7-C8 1.429(10), C8-C9 1.340(11), Os1-Cl1 2.4970(14), C2-P3 1.782(7), Os1-C1-C2 122.5(5), C1-C2-C3 111.2(6), C2-C3-C4 113.2(6), C3-C4-Os1 121.2(5), C4-Os1-C1 71.8(3), Os1-C4-C5 119.3(5), C4-C5-C6 115.3(6), C5-C6-C7 110.2(6), C6-C7-Os1 123.0(5), C7-Os1-C4 72.2(3), Os1-C7-C8 101.4(5), C7-C8-C9 97.5(6), C8-C9-Os1 102.1(5), C9-Os1-C7 58.9(3).

5. Control experiments of the conversion from complex 4 to 5

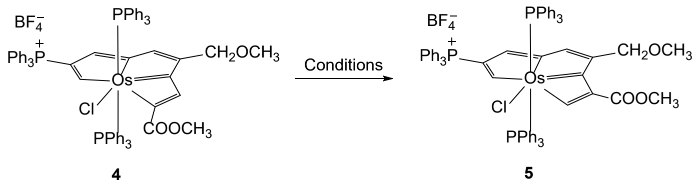

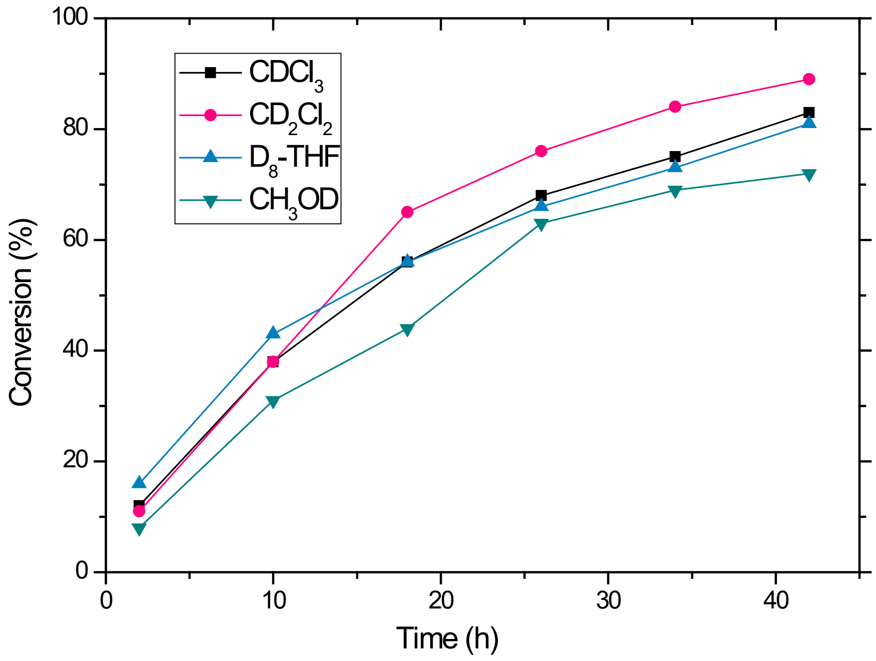

Figure S55 Control experiments of solvents, other condition is at 35 ºC under nitrogen.

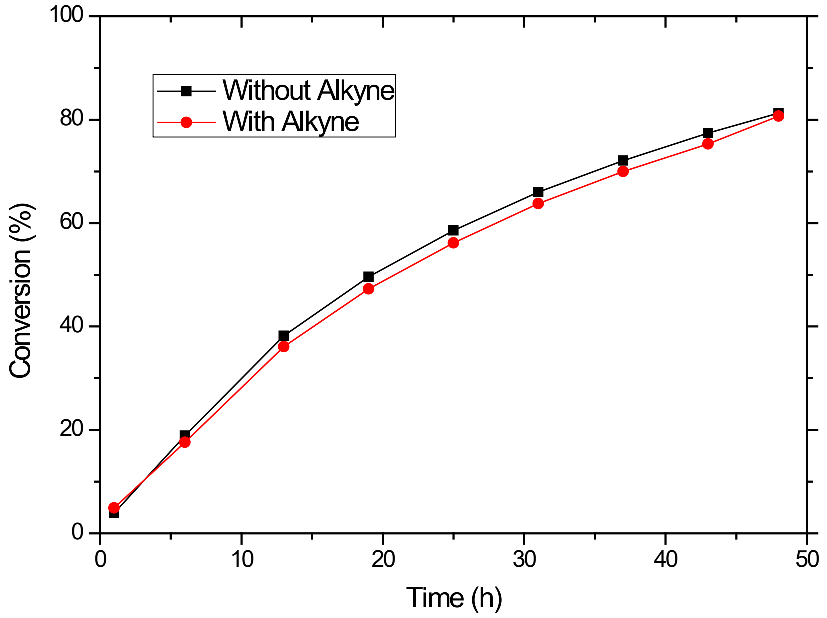

Figure S56 Control experiments of alkyne, other condition is in dichloromethane at 35 ºC under nitrogen.

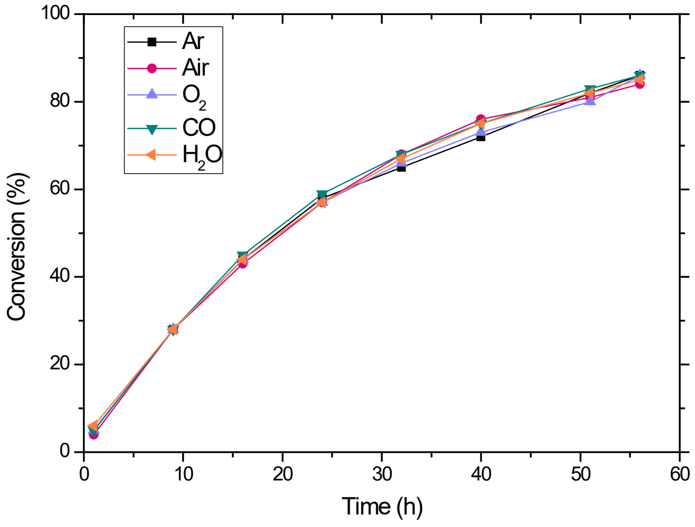

Figure S57 Control experiments of atmosphere, other condition is in dichloromethane at 35 ºC under nitrogen.

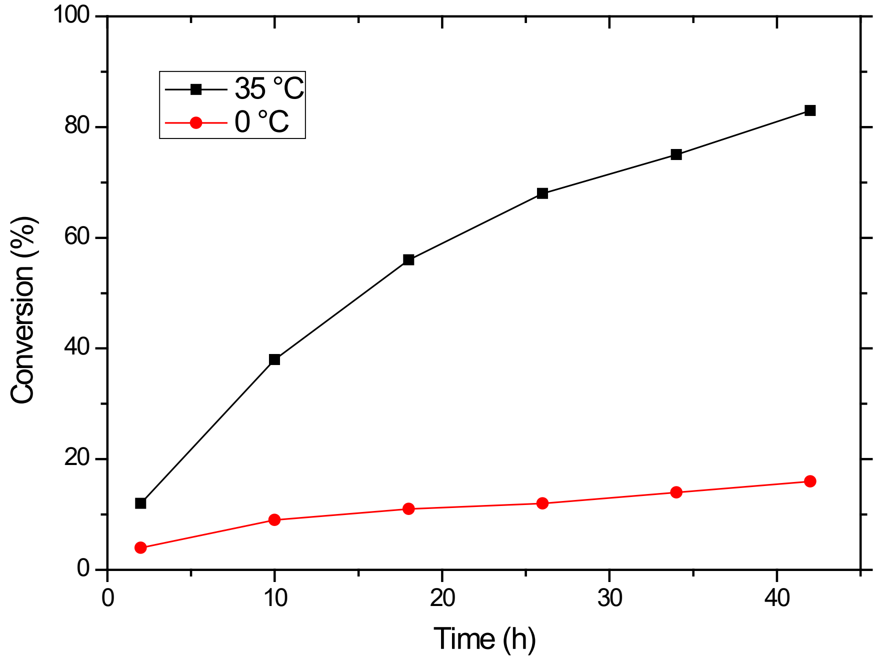

Figure S58 Control experiments of temperature, other condition is in dichloromethane under nitrogen.

6. Computational data

All the calculations were performed with the Gaussian 09 software package.[6] The M06L-D3/6-31G* level[7] of density functional theory was used to optimize all the structures studied in this work. In the calculations, the effective core potentials (ECPs) of Hay and Wadt with SSD were used to describe Os atom,[8] whereas the standard 6-31G* basis set was used for P, Cl, C, O, N, and H atom. The single point energies were then calculated with the level of B3LYP-D3(BJ)/Def2-TZVP with the solvent model of SMD (dichloromethane).[9]

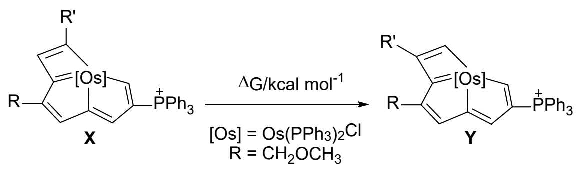

	ΔG/kcal mol^{-1}
**R' = COOH**	-1.9
**R' = COOCH$_3$**	-9.9
**R' = Ph**	-2.8

Figure S59 The free-energy profiles of two osmacyclobutadiene-fused osmapentalene isomers with two reversed regioselectivities.

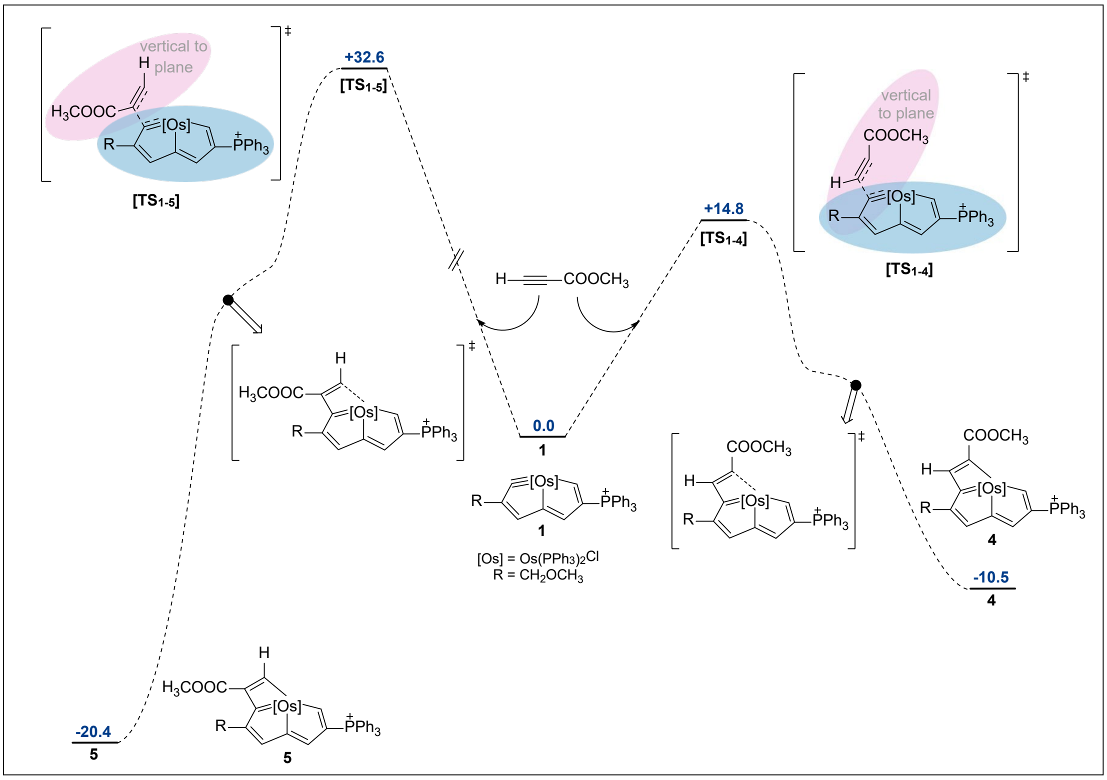

Figure S60 Free-energy profiles of proposed [2+2] cycloaddition reaction pathways of compound **1** and methyl propiolate, unit: kacl/mol.

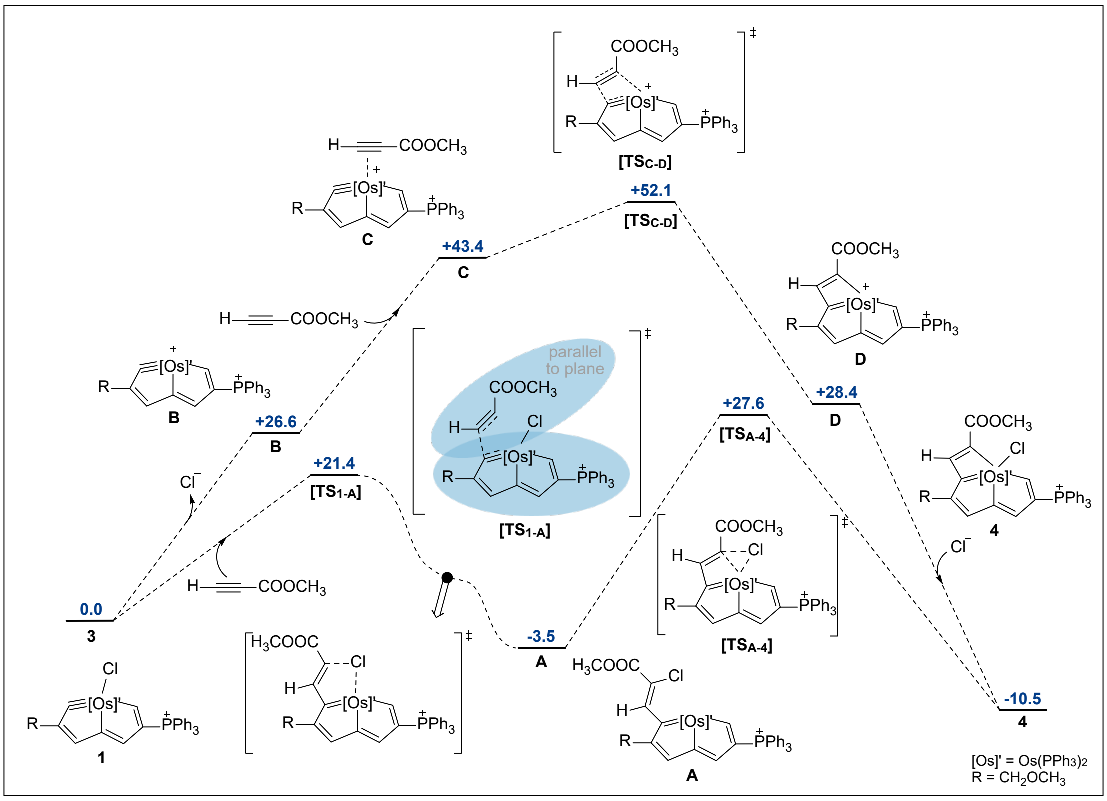

Figure S61 Free-energy profiles of the other possible [2+2] cycloaddition reaction pathways of compound **1** and methyl propiolate, unit: kacl/mol.

7. References

[1] Xia, H.; He, G.; Zhang, H.; Wen, T. B.; Sung, H. H. Y.; Williams, I. D.; Jia, G. Osmabenzenes from the Reactions of HC≡CCH(OH)C≡CH with $OsX_2(PPh_3)_2$ (X = Cl, Br). *J. Am. Chem. Soc.* **2004**, *126*, 6862-6863.

[2] Zhu, C.; Li, S.; Luo, M.; Zhou, X.; Niu, Y.; Lin, M.; Zhu, J.; Cao, Z.; Lu, X.; Wen, T.; Xie, Z.; Schleyer. P. v. R.; Xia, H. Stabilization of Anti-aromatic and Strained Five-membered Rings with a Transition Metal. *Nat. Chem.* **2013**, *5*, 698-703.

[3] Dolomanov, O. V.; Bourhis, L. J.; Gildea, R. J.; Howard, J. A. K.; Puschmann, H. *Olex2*: a Complete Structure Solution, Refinement and Analysis Program. *J. Appl. Cryst.* **2009**, *42*, 339-341.

[4] Sheldrick, G. M. *SHELXT* – Integrated Space-group and Crystal-structure Determination. *Acta. Cryst. Sect. A* **2015**, *71*, 3-8.

[5] Sheldrick, G. M. Crystal Structure Refinement with *SHELXL*. *Acta. Cryst. Sect. C* **2015**, *71*, 3-8.

[6] Frisch, M. J.; Trucks, G. W.; Schlegel, H. B.; Scuseria, G. E.; Robb, M. A.; Cheeseman, J. R.; Scalmani, G.; Barone, V.; Mennucci, B.; Petersson, G. A.; Nakatsuji, H.; Caricato, M.; Li, X.; Hratchian, H. P.; Izmaylov, A. F.; Bloino, J.; Zheng, G.; Sonnenberg, J. L.; Hada, M.; Ehara, M.; Toyota, K.; Fukuda, R.; Hasegawa, J.; Ishida, M.; Nakajima, T.; Honda, Y.; Kitao, O.; Nakai, H.; Vreven, T.; Montgomery, J. A.; Peralta, Jr., J. E.; Ogliaro, F.; Bearpark, M.; Heyd, J. J.; Brothers, E.; Kudin, K. N.; Staroverov, V. N.; Keith, T.; Kobayashi, R.; Normand, J.; Raghavachari, K.; Rendell, A.; Burant, J. C.; Iyengar, S. S.; Tomasi, J.; Cossi, M.; Rega, N.; Millam, J. M.; Klene, M.; Knox, J. E.; Cross, J. B.; Bakken, V.; Adamo, C.; Jaramillo, J.; Gomperts, R.; Stratmann, R. E.; Yazyev, O.; Austin, A. J.; Cammi, R.; Pomelli, C.; Ochterski, J. W.; Martin, R. L.; Morokuma, K.; Zakrzewski, V. G.; Voth, G. A.; Salvador, P.; Dannenberg, J. J.; Dapprich, S.; Daniels, A. D.; Farkas, O.; Foresman, J. B.; Ortiz, J. V.; Cioslowski, J.; Fox, D. J. *Gaussian 09, Revision D.01*, (Gaussian, Inc., Wallingford CT, 2013).

[7] Zhao, Y.; Truhlar, D. G.; A New Local Density Functional for Main-Group Thermochemistry, Transition Metal Bonding, Thermochemical Kinetics, and Noncovalent Interactions. *J. Chem. Phys.* **2006**, *125*, 194101.

[8] Peng, C.; Ayala, P. Y.; Schlegel, H. B.; Frisch, M. J. Using Redundant Internal Coordinates to Optimize Equilibrium Geometries and Transition States. *J. Comput. Chem.* **1996**, *17*, 49-56.

[9] Marenich, A. V.; Cramer, C. J.; Truhlar, D. G. Universal Solvation Model Based on Solute Electron Density and on a Continuum Model of the Solvent Defined by the Bulk Dielectrics Constant and Atomic Surface Tensions. *J. Phys. Chem. B* **2009**, *113*, 6378-6396.
